# Nano-enabled pancreas cancer immunotherapy using immunogenic cell death and reversing immunosuppression

**DOI:** 10.1038/s41467-017-01651-9

**Published:** 2017-11-27

**Authors:** Jianqin Lu, Xiangsheng Liu, Yu-Pei Liao, Felix Salazar, Bingbing Sun, Wen Jiang, Chong Hyun Chang, Jinhong Jiang, Xiang Wang, Anna M. Wu, Huan Meng, Andre E. Nel

**Affiliations:** 1 Division of NanoMedicine, Department of Medicine, David Geffen School of Medicine, University of California, Los Angeles, CA 90095 USA; 2 Center for Environmental Implications of Nanotechnology, California NanoSystems Institute, University of California, Los Angeles, CA 90095 USA; 3 California NanoSystems Institute, University of California, Los Angeles, CA 90095 USA; 4 Department of Molecular and Medical Pharmacology Crump Institute for Molecular Imaging, David Geffen School of Medicine, Los Angeles, CA 90095 USA; 5 Jonsson Comprehensive Cancer Center, University of California, Los Angeles, CA 90095 USA

## Abstract

While chemotherapy delivery by nanocarriers has modestly improved the survival prospects of pancreatic ductal adenocarcinoma (PDAC), additional engagement of the immune response could be game changing. We demonstrate a nano-enabled approach for accomplishing robust anti-PDAC immunity in syngeneic mice through the induction of immunogenic cell death (ICD) as well as interfering in the immunosuppressive indoleamine 2,3-dioxygenase (IDO) pathway. This is accomplished by conjugating the IDO inhibitor, indoximod (IND), to a phospholipid that allows prodrug self-assembly into nanovesicles or incorporation into a lipid bilayer that encapsulates mesoporous silica nanoparticles (MSNP). The porous MSNP interior allows contemporaneous delivery of the ICD-inducing chemotherapeutic agent, oxaliplatin (OX). The nanovesicles plus free OX or OX/IND-MSNP induce effective innate and adaptive anti-PDAC immunity when used in a vaccination approach, direct tumor injection or intravenous biodistribution to an orthotopic PDAC site. Significant tumor reduction or eradication is accomplishable by recruiting cytotoxic T lymphocytes, concomitant with downregulation of Foxp3^+^ T cells.

## Abstract (web summary)

Pancreatic cancer remains difficult to treat mainly due to the drug delivery challenges posed by a strong stromal component. Here the authors develop nanocarriers that improve drug delivery efficiency and engage the host immune system against the tumor resulting in reduction of tumor growth and metastasis.

## Introduction

Pancreatic ductal adenocarcinoma (PDAC) is an almost uniformly fatal disease with a 5-year survival outcome of less than 6%^1^. In spite of its dismal prognosis, the introduction of commercial nanocarriers that deliver paclitaxel (PTX) or irinotecan has had some survival impact^2, 3^. While PTX delivery by an albumin-nanocarrier suppresses the tumor stroma to increase gemcitabine uptake, the delivery of irinotecan by a liposomal carrier improves pharmacokinetics (PK). Moreover, our own studies using mesoporous silica nanoparticles (MSNP) have shown in a robust orthotopic PDAC animal model that it is possible to introduce smart-design features for improving irinotecan loading, efficacy and safety, or deliver a synergistic, ratiometric-designed combination of PTX and gemcitabine^4, 5^.

In addition to improved tumor cell killing, we envisage the use of nanocarriers to deliver chemotherapy in support of PDAC immunotherapy. One possible approach is to use  chemotherapy to induce immunogenic cell death (ICD). Doxorubicin (DOX) is the classical example of inducing an ICD response, which is characterized by apoptotic cell death, accompanied by the expression of calreticulin (CRT) on dying tumor cell surfaces^6^. CRT provides an “eat-me” signal for dendritic cell (DC) uptake^6, 7^. The subsequent release of ATP and a non-histone chromatin protein, high-mobility group box 1 (HMGB-1), from the tumor cells provide adjuvant stimuli to the antigen presenting DC^7^. This cell biological sequence is dependent on the ability of select chemotherapeutic agents, physical stimuli (e.g., irradiation) and cytotoxic viruses to trigger a combination of apoptotic cell death, endoplasmic reticulum stress and autophagy^8–12^.

**Fig. 1:**
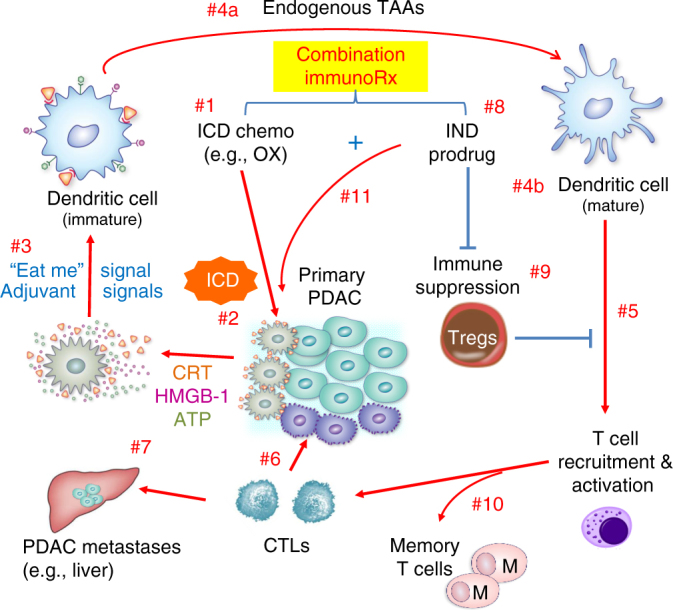
Schematic to illustrate how dual delivery of OX and IND may impact the anti-PDAC immune response. We hypothesized that nano-enabled co-delivery of a chemotherapeutic agent, which provides an ICD stimulus, and IND, which interferes in the IDO pathway, may combine to trigger a robust PDAC immune response. OX (#1) induces an ICD response (#2) in which CRT expression on the dying tumor cell surfaces provides an “eat-me” signal for DC uptake, as well as the release of HMGB-1 and ATP that deliver adjuvant stimuli to DC (#3). Following uptake of the dying tumor cells by DC, their maturation and cross-presentation of endogenous tumor-associated antigens (TAAs) (#4), the recruitment and activation of CD8^+^ T cells (#5) will lead to granulysin and perforin mediated killing of primary (#6) and metastatic cancer cells (#7). The concomitant delivery of IND-PL (#8) interferes in the IDO metabolic pathway, which can lead to strengthening the ICD effect by interfering in Treg development and overcome other immunomodulatory effects (#9). The ICD pathway also allows the activation of helper and memory T cells, which prevent disease recurrence (#10). Following proof-of-prinipal testing of this scheme, we also discovered that IND syngergistically enhances the ICD effect, providing more than just an additive outcome  (#11)

Oxaliplatin (OX), one of the four components in the FOLFIRINOX chemotherapy regimen used in PDAC, can also induce an ICD response in various cancer cells, including pancreatic cancer cells^13^. We hypothesized that encapsulated OX delivery to the PDAC site may allow us to induce a regional ICD effect. We also posited that the immunogenic effects of OX could be enhanced if we reverse the immunosuppressive effects of the regionally overexpressed metabolic enzyme, indoleamine 2,3-dioxygenase 1 (IDO1), at the PDAC site. IDO1 controls an immune surveillance pathway in the tumor microenvironment (TME) by catalyzing a rate-limiting step in the kynurenine pathway^14–17^. By converting L-tryptophan (Trp) to L-kynurenine (Kyn), IDO1 restricts Trp availability in tumor cells and innate immune cells; this triggers effector pathways that interfere in the development of cytotoxic T cells, while inducing Tregs^18, 19^. These immunosuppressive effects can be rescued by 1-methyl-D-tryptophan (a.k.a. indoximod, IND)^20, 21^, a small molecule inhibitor that is poorly retained at the tumor site^22, 23^. We argued that a change in the PK of this drug could be an additional benefit of a nano-enabled approach^24^. Figure 1 illustrates our conceptual thinking of using a dual delivery system for OX plus IND to develop an effective immunotherapy approach for PDAC, premised on an ICD stimulus plus interference in the IDO pathway.

We report the design of soft and hard nanocarrier platforms for delivery of OX plus a lipid-conjugated IND prodrug. We demonstrate the feasibility of achieving tumor regression or eradication of a Kras-derived PDAC model, using a vaccination approach, local tumor injection or systemic administration. The employment of a nano-enabled platform was critical for improving the PK of drug delivery, intratumoral drug concentrations, and duration of action. In addition to providing immunostimulation, the IND prodrug had the unexpected benefit of synergistically enhancing the ICD response, with boosting of innate and adaptive anti-PDAC immunity.

## Results

### Oxaliplatin induces an ICD response in PDAC

**Fig. 2:**
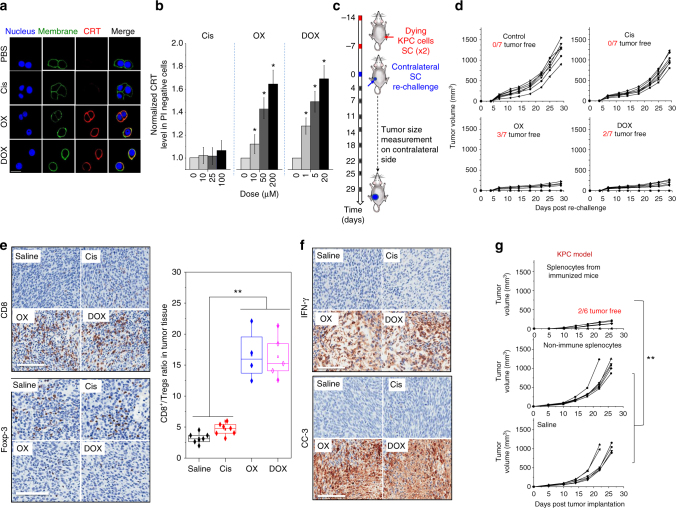
Oxaliplatin-induced ICD provides a successful anti-PDAC vaccination approach. **a** Confocal microscopy showing the induction of the ICD marker, CRT, in  KPC cells in the presence of PBS, Cis (100 µM), OX (50 µM), and DOX (1 µM) for 4 h. The cell nuclei, surface membrane and CRT were detected by Hoechst 33342, Alexa Fluor^®^ 488-Conjugated Wheat Germ Agglutinin, and Alexa Fluor^®^ 647-conjugated anti-CRT antibody staining, respectively. Scale bar is 20 μm. **b** CRT surface detection by flow cytometry, using the same conditions and reagents as in **a** (3 independent experiments). **c** Animal experimentation using 2 rounds of vaccination one week apart, followed by injecting live KPC cells SC on the contralateral side. The details of the animal vaccination experiment are provided in the methods section. Tumors were collected on day 29 for IHC and flow cytometry analysis. **d** Spaghetti curves to show KPC tumor growth in the contralateral flank. **e** Tumor collection was performed after euthanizing the animal to conduct IHC. Representative images are shown for the IHC staining of CD8 (upper panel) and Foxp3 (lower panel) T cells. The tumor tissues were also analyzed by flow cytometry to determine the CD8/Tregs ratio (see experimental section for details) (right panel). **f** IHC staining for cleaved caspase-3 (CC-3) and IFN-γ to demonstrate recruitment of cytotoxic T cells in response to ICD. Scale bar in IHC is 100 μm. **g** The 3 surviving animals in the OX-treated group, described in **c**, received orthotopic implant of live KPC cells on day 74. Animals maintained their tumor-free status up to 132 days, whereupon they were euthanized for collecting the immune splenocytes to perform an adoptive transfer experiment. IV injection of the immune splenocytes into the tail vein of B6/129 mice prevented the growth of KPC cells, implanted SC. The controls included IV administration of non-immune splenocytes or saline. The same experiment was also carried out in mice receiving SC injection of B16 melanoma cells. In this case, there was no interference in tumor growth by immune splenocytes, demonstrating the antigen specificity of the adoptive transfer response (Supplementary Fig. 3). The results are expressed as mean ± SEM. *p < 0.05; **p < 0.01, (ANOVA)

ICD is a modified form of apoptosis that can be used to initiate an effective immune response against endogenous tumor antigens^7^. Although ICD is best described for anthracycline chemotherapeutics (e.g., DOX), we were interested in finding a recognized PDAC drug to provide the same stimulus. OX is FDA-approved for PDAC treatment, and has been shown to induce ICD in PDAC cancer cells^13^. We initiated a screen for CRT expression in human and mouse PDAC cell lines, in which OX was compared with DOX and cisplatin  (Cis). KPC cells were derived from a spontaneous PDAC tumor that developed in a transgenic Kras^LSL-G12D/+^/Trp53^LSL-R172H/+^/Pdx-1-Cre (KPC) mouse^25^. While OX and DOX treatment induced CRT expression on the surface of KPC cells as viewed by confocal microscopy, no surface expression was seen for Cis (Fig. 2a). More quantitative evaluation by flow cytometry confirmed the dose- and time-dependent effects of OX and DOX (Fig. 2b and Supplementary Fig. 1a). A similar stress response was observed in the human PANC-1 pancreatic cancer cell line (Supplementary Fig. 1b), as well as using an ELISA to measure HMGB-1 release in both cell types (Supplementary Fig. 1c).

The gold standard for confirming ICD in vivo is a vaccination response in a syngeneic animal model^7^. KPC cells can be grown subcutaneously (SC)  to tumors in immune competent B6/129 mice. To allow bioluminescence imaging of the tumor site, KPC cells were transfected with a luciferase vector^4^. We asked whether ex vivo exposure of KPC cells to above chemo agents can induce an adequate immune response to prevent KPC growth SC. Suspensions of dying tumor cells, generated by exposure to OX (50 µM), DOX (1 µM), or Cis (100 µM)  for 24 h, were SC injected on 2 occasions (7 days apart) in one flank of the animals. The animals was subsequently challenged by SC injection of live KPC cells on the contralateral flank, 7 days later (Fig. 2c). While vaccination with OX- or DOX-treated cells significantly suppressed tumor growth on the contralateral side, Cis treatment had no effect (Fig. 2d). The magnitude of the growth inhibition was confirmed by IVIS imaging (Supplementary Fig. 2a). Notably, 3 (out of 7) mice in the OX-treated group and 2 (out of 7) mice in DOX-treated group survived tumor-free. The rest of the animals were killed on day 29 for immunohistochemistry (IHC) and flow cytometry analysis.

IHC revealed increased tumor staining for CD8^+^ T cells in parallel with a decreased regulatory (Foxp3^+^) T cell component in animals vaccinated with OX or DOX-treated cells (Fig. 2e). Cis treatment had no effect. Quantitative assessment of the same biomarkers using flow cytometry and single-cell suspensions, demonstrated 5.1- and 5-fold increase in the CD8^+^/Tregs cell ratios in the OX and DOX vaccinated groups, respectively, compared to saline (Fig. 2e, right panel). Since elevation of the CD8^+^/Tregs ratio is compatible with a cytotoxic response, IHC staining was used to confirm the appearance of activated (cleaved) caspase-3 (CC-3) and IFN-γ (Fig. 2f) at the tumor sites of animals vaccinated with OX or DOX-treated cells.

The three surviving animals in the OX-induced ICD group were used for orthotopic implantation of KPC cells in the pancreas on day 74. No orthotopic tumors emerged up to day 132, compared to fatality in non-vaccinated animals within 30 days. The surviving, prior vaccinated and orthotopic-challenged animals, were euthanized on day 132 to collect splenocyte populations for adoptive transfer to non-immune animals. The splenocytes were intravenously (IV) injected into the tail vein of 12 non-immunized B6/129 mice. The control was a group of 12 animals receiving IV injection of splenocytes collected from non-immune animals or animals treated with saline only. Each of the three groups was divided in half, with 6 animals receiving SC injection of live KPC cells and the rest being injected with B16 melanoma cells. Monitoring of tumor growth demonstrated a significant reduction in KPC growth in animals injected with immune splenocytes, compared to animals receiving non-immune splenocytes or saline only (Fig. 2g). Two of the six mice receiving immune splenocytes survived tumor-free. No impact was seen on B16 tumor growth (Supplementary Fig. 3). These results indicate that OX treatment generates an ICD effect that culminates in a memory T cell response for PDAC. An abbreviation list was provided for the ease of reading (Supplementray Table 1).

### Synthesis of the IND prodrug for immunomodulatory therapy

IDO1 is frequently overexpressed in the solid TME, where its metabolic action of converting Trp to Kyn can interfere in the proliferation of cytotoxic T cells, expansion of Tregs and interference in memory T cell development^18, 19^. A number of small molecule inhibitors of the IDO effector pathway have been developed for cancer treatment, including IND^20, 21^. While IND is currently being tested in several clinical trials (including PDAC), its utility as a stand-alone immunostimulatory agent appears to be modest and is often combined with other treatment modalities^23, 24^. Oral administration requires a high dose (up to 1200 mg b.i.d.)^26^ to compensate for its poor water solubility, rapid blood clearance and limited accumulation at the tumor site^27^. These potentially unfavorable PK in humans was corroborated by the animal data, in which we observed that IV administration had a short circulatory half-life (t_1/2_) of <0.083 h, with <0.1% of the injected IND dose gaining access to the tumor site (Supplementary Fig. 4i).

**Fig. 3:**
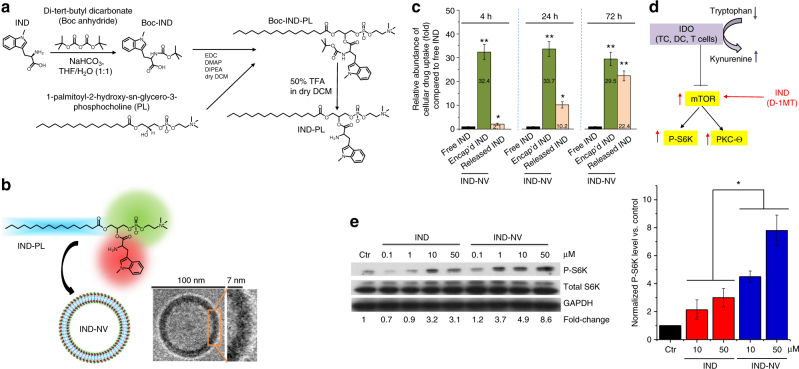
Synthesis of a self-assembling indoximod (IND) prodrug for immune modulatory activity. **a** Detailed synthesis and characterization for generating the phospholipid-conjugated IND prodrug (IND-PL) appears in Supplementary Fig. 4. Successful synthesis of IND-PL was confirmed by a calculated *m/z* of 696.4353 during ESI-MS (Supplementary Fig. 4f, g). **b** Illustration depicting self-assembly of IND-PL nanovesicles (IND-NV), with IND securely anchored in the lipid bilayer. A representative cryoEM image of the spherical IND-NV, with diameter ~80 nm and lipid bilayer thickness of ~7 nm is shown as well. A lower magnification cryoEM picture is shown in Supplementary Fig. 4h. **c** UPLC-MS/MS to determine the cellular uptake and release of IND-PL. KPC cells were treated with 100 µg/mL free IND or IND-NV for the indicated incubation period, followed by collection of cells (via trypsinization) and drug extraction. The data show the fold-increase of the intracellular drug concentration as compared to free IND. A typical UPLC-MS/MS readout is shown in Supplementary Fig. 5. Details about the sample preparation and analysis are described in Supplementary Fig. 5. Three independent experiments were performed. **d** Role of IDO in providing immune suppression in the TME by inhibiting the mTOR pathway through Trp depletion. IND rescues this interference, acting as a highly potent Trp mimetic. This rescue leads to the phosphorylation and activation of P-S6K, as well as activation of PKC-ϴ that is involved in signal transduction by the T-cell antigen receptor; **e** KPC cells were treated with free IND or IND-NV at the indicated concentrations for 3 h in tryptophan-deficient DMEM. Western blot assays showing the enhanced effect of IND-PL on mTOR signaling, which can be conveniently studied by assessing the phosphorylation of P-S6K (upper panel). The graphic in the right panel shows the pooled data for 3 experiments to assess P-S6K activation at 10 μM and 50 μM IND. The results are expressed as mean ± SEM. *p < 0.05; **p < 0.01, (ANOVA)

We hypothesized that the biodistribution, retention and PK of IND at the tumor site can be improved by a nano-enabled drug design approach that prolongs the duration of action. An IND prodrug was constructed by using the labile ester bond to conjugate 1-methyl-D-Trp to a single-chain phospholipid, 1-palmitoyl-2-hydroxy-sn-glycero-3-phosphocholine (PL) (Fig. 3a). The conjugation reaction was accomplished by the following steps: (i) Boc protection of the IND amine group, (ii) esterification of Boc-IND with the PL, and (iii) Boc removal (Fig. 3a). The detailed synthesis and characterization are described in Supplementary Fig. 4. When aqueously suspended, amphiphilic IND-PL self-assembles into spherical ~80 nm nanovesicles (IND-NVs), demonstrated by cryo-electron microscopy (cryoEM) (Fig. 3b and Supplementary Fig. 4h). UPLC-MS/MS was used to study IND uptake and release in KPC cells (Supplementary Fig. 5). Compared to free IND, the total intracellular IND content increased 34.5, 43.9, and 51.9-fold after exposure to IND-NV at 4, 24, and 72 h in cells collected by trypsinization (Fig. 3c). Increased cellular uptake was accompanied by increased IND release from the prodrug (Fig. 3c). An abiotic study confirmed that esterase activity and acidification of the incubation medium can release the IND from the prodrug; similar to what may happen in a mammalian cell (Supplementary Fig. 5c).

IDO-mediated Trp depletion and Kyn accumulation leads to the perturbation of downstream effector pathways involved in immune suppression^14, 21, 28^. Key among these, is the mTOR pathway that is sensitive to Trp depletion, resulting in interference in the phosphorylation and activation of P-S6 kinase  (P-S6K) (Fig. 3d), as well as inhibition of autophagy^17^. In accordance with the increased bioavailability of IND-PL vs. free IND, anti-phosphopeptide immunoblotting demonstrated that the conjugated drug is better capable of enhancing P-S6K phosphorylation (Fig. 3e). In addition to studying S6K phosphorylation, we also looked at the effect of the IDO pathway in controlling the aryl hydrocarbon receptor (AhR) pathway that engages the autocrine AhR-IL-6-STAT3 signaling loop, putatively responsible for sustained IDO expression (Supplementary Fig. 6b)^28^. Noteworthy, IND-NV was more potent than the free drug in interfering in IL-6 production in KPC cells (Supplementary Fig. 6c).

### The IND prodrug synergizes with OX at the PDAC tumor site

**Fig. 4:**
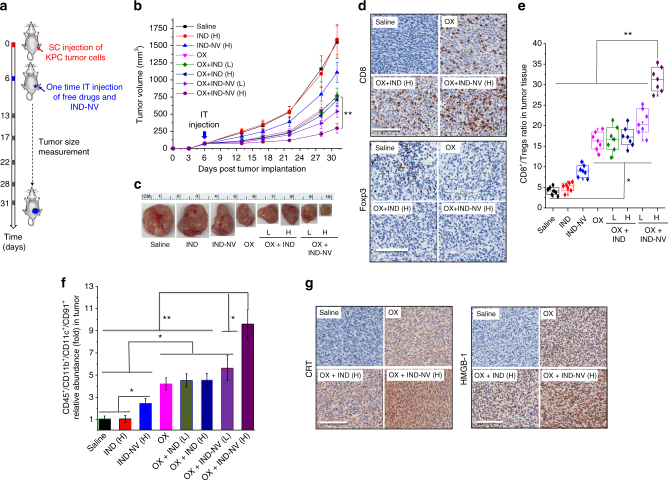
Co-administration of IND-NV with OX at the tumor site augments anti-PDAC immunity. **a** Local co-administration in KPC tumors established by SC injection in syngeneic mice. Treatment details are provided in the methods section. **b** KPC tumor growth curve after a single IT injection of the various drugs at a tumor size of 60–80 mm^3^. OX was injected at 1.25 mg/kg^15^. Low (L, 2.5 mg IND/kg) and High (H, 12.5 mg IND/kg) refer to the IND or IND-NV doses. **c** Representative tumor images from each group after euthanizing the animal on day 31. **d** IHC depicting CD8 and Foxp3 biomarkers in the collected tumor tissue. A full set of panels of the IHC staining data are shown in Supplementary Fig. 7a–j. **e** Flow cytometry determination of CD8/Tregs ratio, as described in **d**. **f** Flow cytometry analysis to determine CD91 expression in the population of CD45^+^/CD11b^+^/CD11c^+^ cells in the tumor tissue. **g** IHC to depict CRT and HMGB-1 expression in the collected tumor tissues. The results are expressed as mean ± SEM. *p < 0.05; **p < 0.01, (ANOVA). The scale bar is 100 μm

Because the vaccination approach does not address the immunosuppressive TME in an established tumor, we asked whether IND-PL could strengthen the OX-induced ICD response in KPC tumors grown SC (Fig. 4). Following tumor growth to 60–80 mm^3^ size, the mice received a single intratumoral (IT) injection of 1.25 mg/kg OX^15^ plus either a low (L) (2.5 mg/kg IND) or a high (H) (12.5 mg/kg IND) dose of IND-NV IT (Fig. 4a). Controls included animals receiving injection with saline, free OX (1.25 mg/kg), free IND (12.5 mg/kg), or IND-NV (12.5 mg/kg IND) alone. Serial assessment of tumor volume, followed by euthanizing of animal on day 31 for in situ inspection of tumor size (Fig. 4c) demonstrated that OX plus IND-NV (H) had the most robust tumor reduction effect, while OX plus IND-NV (L) or OX plus free IND (L or H) had lesser potency (Fig. 4b, c). Free IND had no effect on tumor growth, while IND-NV alone exerted a small effect (Fig. 4b, c).

The resected tumor tissues were used for IHC and multi-parameter flow cytometry analysis. IHC staining for CD8 and Foxp3 showed that OX plus IND-NV (H) resulted in significantly enhanced recruitment of CD8^+^ T cells along with a reduction in Foxp3^+^ T cells (Fig. 4d). Moreover, the comprehensive IHC profiles shown in Supplementary Fig. 7a–k demonstrate good responsiveness to OX alone, OX plus IND-NV (L), and OX plus IND (H or L), although not as prominent as OX plus IND-NV (H). These findings were corroborated by flow cytometry, demonstrating that the co-administration of OX plus IND-NV (H) synergistically enhance the CD8^+^/Tregs ratio compared to other treatments (Fig. 4e).

Since IDO also plays a significant role in DC, we asked whether IND co-administration could impact the innate immune events that underpin tumor antigen presentation. In this regard, CD91 expression on DC serves as the binding receptor for CRT^+^ tumor cells, while TLR4 is engaged by released HMGB-1^6, 7, 16, 17^. Multi-parameter flow cytometry showed that OX plus IND-NV (H) induced the most abundant CD91 expression in a CD45^+^/CD11b^+^/CD11c^+^ cell population, compared to animals treated with free drugs (Fig. 4f). The phenotypic data were also confirmed by IHC staining for CD91 in the tumor tissue (Supplementary Fig. 7e). Moreover, IHC analysis of CRT and HMGB-1 expression demonstrated more abundant expression during treatment with OX plus IND-NV (H) (Fig. 4g). The comprehensive IHC profiles for CRT and HMGB-1 showed less robust responses to OX, OX plus IND (H and L) and OX plus IND-NV (L) (Supplementary Fig. 7d, f). Likewise, assessment of TLR4 expression by IHC or flow cytometry analysis of CD45^+^/CD11b^+^/CD11c^+^ cells confirmed the synergy between OX and IND-NV (H and L) (Supplementary Fig. 7g). Local OX plus IND-NV (H) administration was also accompanied by significant IFN-γ release, in parallel with increased staining for CC-3 (Supplementary Fig. 7h, j). This was accomplished by decreased abundance of the anti-inflammatory cytokine, IL-10, which contributes to immune suppression (Supplementary Fig. 7i). All considered, these data confirm that, in addition to the impact on the adaptive immune system, the combination of free OX plus IND-NV has unique stimulatory effects on the innate immune system.

### Development of a dual delivery carrier for OX plus IND-PL

**Fig. 5:**
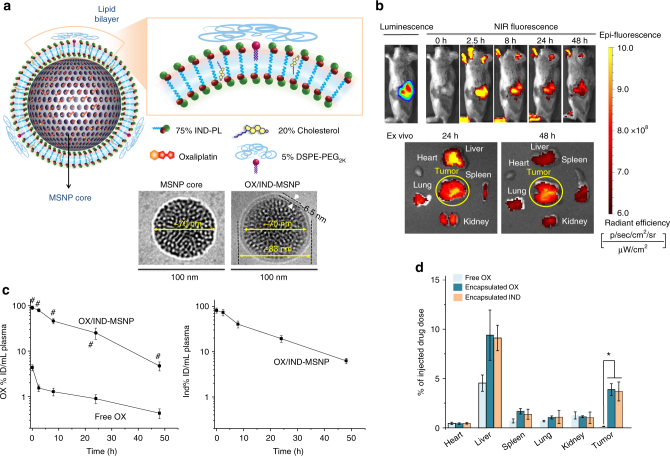
Development of a dual delivery carrier for OX plus IND using lipid-bilayer coated mesoporous silica nanoparticles (OX/IND-MSNP). **a** Schematic to show the structure of OX-laden MSNP, in which the drug is trapped by a lipid bilayer (LB) that contains the IND-PL. This leads to stable entrapment of OX in the pores, with IND-PL trapped in the bilayer. The coating procedure provides uniform and instantaneous sealing of the particle pores. The development of an optimized lipid coating mixture (75% IND-PL, 20% cholesterol, and 5% DSPE-PEG_2K_), is described in Supplementary Fig. 8a. The CryoEM picture shows a spherical MSNP core and its coated lipid bilayer. CryoEM imaging of ~100 particles demonstrated that the average particle size of the MSNP core was ~70 nm, while that of the LB-coated particles was ~83 nm (including a 6.5 nm thick lipid bilayer). CryoEM images for the control OX/LB-MSNP particles demonstrated a particle size of ~82 nm (Supplementary Fig. 8d). Low-magnification cryoEM images are provided in Supplementary Fig. 8c, d. **b** IVIS optical imaging to study the biodistribution of IV OX/IND-MSNP in orthotopic-implanted KPC tumors in mice (*n* = 6) at the indicated time points. Dylight 680-labeled DMPE was used for NIR imaging. Ex vivo imaging was performed for tumor, heart, liver, spleen, kidneys, and lung tissue collected from the animals 24 and 48 h post injection. **c** A separate experiment evaluated the PK profile of OX/IND-MSNP in orthotopic tumor-bearing mice (*n* = 6), receiving single IV injection to deliver the equivalent 5 mg/kg OX and 50 mg/kg IND. Free OX served as a control. Plasma was collected after 0.083, 2, 8, 24 and 48 h, and used for the analysis of IND, IND-PL, and silicon (Si) content, as described in the methods section. **d** The tumors and major organs were collected after 48 h for analysis of the tissue content of OX, IND, and Si. The results are expressed as mean ± SEM. ^#^ *p* < 0.001, (ANOVA).

An IV injectable carrier was established for dual delivery of OX plus IND in the orthotopic KPC model, which closely mimics the growth and metastatic profile of human PDAC^29–31^. We chose a lipid bilayer (LB) coated MSNP platform based on drug loading capacity, stability, and effective biodistribution to orthotopic PDAC sites by a transcytosis mechanism^4, 32^. The MSNP platform has been used extensively for the drug delivery in cancer therapy^4, 5, 32–37^. The increased ability of a supported LB over that of nanovesicles constituted another reason for the choice of MSNPs. Moreover, the LB can also be used to incorporate IND-PL, while serving at the same time to encapsulate OX in the porous interior (Fig. 5a). Optimal design of the LB was accomplished by employing an IND-PL/Cholesterol/DSPE-PEG_2K_ mixture at a molar ratio of 75:20:5 (Supplementary Fig. 8a). The biofilm was laid down at the bottom of a round bottom flask, to which the OX-soaked MSNPs were added, followed by sonication, particle purification and washing^4, 5^. As a control, we synthesized a MSNP in which OX was encapsulated in an IND-PL free carrier (OX/LB-MSNP). CryoEM images of the dual-delivery (Fig. 5a and Supplementary Fig. 8c) and OX/LB-MSNP (Supplementary Fig. 8d) carriers showed particles of ~ 83 nm and ~ 82 nm in size, respectively. The particles are uniformly coated with an intact LB, ~ 6.5 nm thick, and slight-negative zeta potential. The OX loading capacities for OX/LB-MSNP and OX/IND-MSNP were 4.5% and 4.4%, respectively (Supplementary Fig. 8c, d). The particles had good colloidal stability in biological media for up to 30 days (Supplementary Fig. 8b).

To visualize the biodistribution of the IV-injected OX/IND-MSNP, 0.1% w/w Dylight 680-labeled DMPE was incorporated into the lipid biofilm. This allowed IVIS imaging of the near-infrared (NIR) labeled particles at the orthotopic tumor site, as early as 2.5 h (Fig. 5b, upper panel). Particles were retained at the tumor site for up to 48 h, and could also be observed in the explanted organs, obtained from euthanized animals (Fig. 5b, lower panel). The ex vivo images also showed biodistribution to the liver and spleen, independent from the tumor site. The PK of OX and IND were assessed using WinNonlin software after tumor-bearing mice were IV injected with the OX/IND-MSNP carrier (5 mg/kg OX, 50 mg/kg IND) (Fig. 5c). Blood was collected at the indicated time points (0.083, 2, 8, 24 and 48 h) to measure OX, IND and silicon (Si) concentrations. The 1^st^ measurement at 5 min (0.083 h) represents *C*_max_. Animals were euthanized at 48 h to assess the IND content of the collected organs by UPLC-MS/MS (Fig. 5d). Compared to free OX, lipid-coated MSNP encapsulation significantly prolonged the circulatory t_1/2_ of this drug from <0.083 h to 10.4 h (Fig. 5c, left panel). The t_1/2_ of IND was 9.5 h, which is significantly longer than the t_1/2_ <0.083 h for free IND (Fig. 5c, right panel, Supplementary Fig. 4i). These results agree with calculation of t_1/2_ for Si, which was determined to be 9.2 h (Supplementary Fig. 9). Calculated as a % of the total injected dose, ∼4 wt% of OX and IND could be seen to distribute to the tumor site by 48 h (Fig. 5d). The % ID at the tumor site is in line with data obtained for FDA-approved nanoparticles such as Abraxane (~ 2.4%)^38^, Doxil (~ 3%)^39, 40^, and Onivyde (~ 2%)^41^.

### Effective anti-PDAC immunity by a dual delivery carrier

**Fig. 6:**
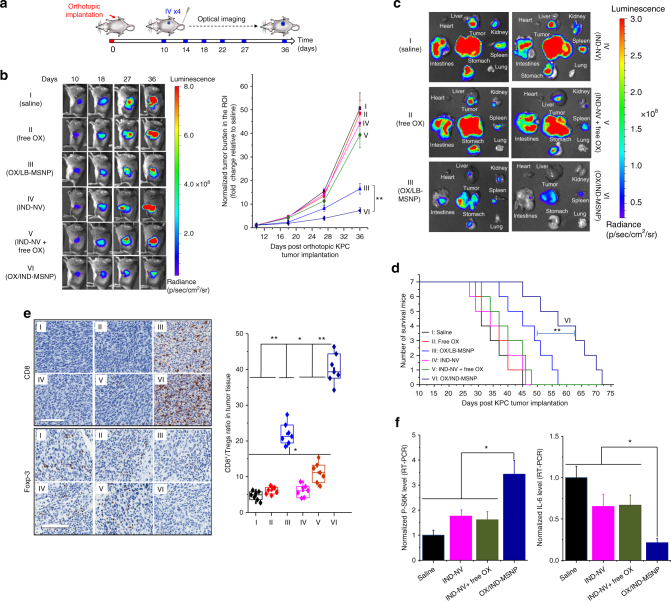
Dual delivery of OX plus IND-NV by MSNP induced effective anti-PDAC immunity in the orthotopic tumor model. **a** Orthotopic tumor-bearing B6/129 mice (*n* = 7) were IV injected with the OX/IND-MSNP to deliver the equivalent 5 mg/kg OX and 50 mg/kg IND every 4 days, for a total of 4 administrations. The 1^st^ injection started on day 10. Free OX, OX/LB-MSNP, IND-NV, IND-NV + free OX, and OX/IND-MSNP were used for comparison at the equivalent doses. Interval IVIS imaging monitored tumor growth, which was quantitatively expressed as image intensity at the ROI. **b** Representative IVIS imaging on days 10, 18, 27, and 36, according to which the normalized tumor burden was plotted as fold-increase compared to the non-treated control. **c** Representative ex vivo bioluminescence imaging on day 36 to show the effect of treatment on metastatic tumor spread to the stomach, intestines, liver, spleen, kidneys, diaphragm, and abdominal wall, but not the heart or lung. We also included in the same experiment, treatment with anti-CD8 and anti-TLR4 antibodies, as well as an injectable  pool of siRNAs for knockdown of CD91. The effect of interference in the immune response is shown in Supplementary Fig. 12a. The corresponding heat map display of the ex vivo imaging is summarized in Supplementary Fig. 10b. **d** Assessment of the survival effect of OX/IND-MSNP (*n* = 7) vs. the controls was conducted by repeating the experiment in (**a**). **e** IHC staining for CD8^+^ and Foxp3^+^ T cells in tumor tissue, collected in **c** (left panel). Scale bar is 100 μm. CD8/Tregs ratio in tumor tissue determined by flow cytometry (right panel). **f** Real-time PCR measurement of P-S6K and IL-6 mRNA expression as a result of interference in the IDO pathway in vivo. The results are expressed as mean ± SEM. *p < 0.05; **p < 0.01. (ANOVA for Fig. 6b, e, f; Log-rank Mantel–Cox test for Fig. 6d)

Following orthotopic implantation of luciferase-expressing KPC cells in the pancreas of B6/129 mice, the animals were IV injected with the OX/IND-MSNP carrier on days 10, 14, 18, and 22 (Fig. 6a). Each animal received a MSNP dose of 111 mg/kg (equivalent 5 mg/kg OX and 50 mg/kg IND per injection). The controls included mice receiving saline, free OX, OX/LB-MSNP (without IND-PL), IND-NV only, or IND-NV plus free OX at equivalent doses. Monitoring of orthotopic tumor growth by IVIS imaging and calculating luminescence intensity (Fig. 6b) demonstrated significantly higher rates of tumor shrinkage for OX/IND-MSNP treatment compared to controls, including OX/LB-MSNP. Autopsies were performed to allow ex vivo IVIS imaging of the primary tumor site, surrounding organs and metastatic sites (Fig. 6c). The imaging data were confirmed by visual inspection during autopsy (Fig. 6c). This established highly significant reduction in the primary tumor size and metastatic spread during dual-delivery treatment or administration of OX/LB-MSNP (Supplementary Fig. 10a, b). The rest of the controls showed large primary tumors and numerous metastatic foci. Kaplan–Meier plots confirmed that while OX/LB-MSNP improved survival, the dual-delivery carrier had a significant survival benefit (Fig. 6d). Additional assessment of innate and adaptive immune features  was carried out using IHC staining and multi-parameter flow cytometry. Assessment of CD8, Foxp3, CRT, CD91, HMGB-1, TLR4, IL-12p70, IFN-γ, perforin, IL-10 and CC-3 expression confirmed the observations of the IT injection model (Fig. 6e, Supplementary Fig. 11a–k). This confirms that the dual-delivery carrier was capable of inducing a cytotoxic T cell response, disappearance of Tregs and induction of innate immunity. The CD8^+^/Foxp3^+^ ratio improved further in response  to OX/IND-MSNP carrier, demonstrating the unexpected synergy that can be achieved by dual drug delivery (Fig. 6e). Assessment of mature CD11c^+^/CD80^+^/CD86^+^ DCs amounted to ~ 32.4% in animals treated with the OX/IND-MSNP, which is significantly higher than animals treated with saline (3.4%), free OX (4.9%), OX/LB-MSNP (17.8%), IND-NV (5.4%), and IND-NV + free OX (7.2%). We also observed an increase in CD103 expression in CD45^+^/CD11b^+^/CD11c^+^ cells by dual drug delivery (Supplementary Fig. 11l); these DCs are particularly adapted for instructing CD8^+^ T cell development and antitumor immunity^42, 43^.

On the basis of the critical role of CD8-mediated cytotoxicity and the role of CD91 and TLR4 in the innate immunity (Fig. 4, Supplementary Fig. 7), we asked, as an extension of the results in Fig. 6b, whether IV injection of antibodies to CD8 and TLR4^44^ or an injectable pool of siRNAs targeting CD91^45^, could interfere with the protective immune response observed in this experiment^45^ (Supplementary Fig. 12). Notably, these treatments had a significant inhibitory effect on the ability of OX/IND-MSNP to shrink tumor growth, prolong survival, or ability to increase the CD8^+^/Tregs ratio (Supplementary Fig. 12a–d). This was also reflected by IHC staining and the flow cytometry results (Supplementary Fig. 11a–l).

The dual-delivery particle was well-tolerated in animal safety studies, without evidence of weight loss, increased liver enzymes (Supplementary Fig. 13) and interference in organ histology. In contrast, free OX increased AST, ALT, and ALP levels.

To validate the involvement of the IDO metabolic pathway in the antitumor response, the collected tumor tissue was used to analyze the expression of P-S6K and IL-6 mRNA. P-S6K was significantly upregulated with decreased IL-6 levels in tumors from OX/IND-MSNP-treated animals (Fig. 6f). These data agree with the in vitro results (Fig. 3e and Supplementary Fig. 6c).

### Immuno-PET imaging confirms anti-PDAC immunity

**Fig. 7:**
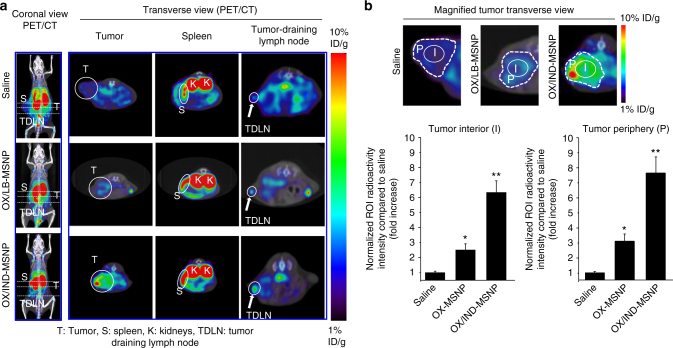
Immuno-PET imaging to demonstrate the induction of the systemic immune response by OX/IND-MSNP administration to animals carrying orthotopic KPC tumors. **a** Animals with established orthotopic tumors (*n* = 3/group) were IV injected with saline, OX/LB-MSNP (5 mg/kg OX), and OX/IND-MSNP (5 mg/kg OX and 50 mg/kg  IND on days 10, 14, 18, and 22 post KPC cell implantation into the pancreas. At day 26, 100 µL doses containing 1.07–2.33 MBq (29–63 µCi, 2.3–5.3 µCi/µg)^89^Zr radiolabeled cDb in saline was IV injected to the same animals. 20 h later, microPET and CT scans were acquired by a G8 PET/CT scanner (Sofie Biosciences). Coronal (left panel) and transverse views (right panel) were acquired and analyzed by AMIDE software. OX/IND-MSNP-treated mice showed significantly increased radioactivity in the tumor, spleen, and TDLN, corresponding to the induction and infiltration of CD8^+^ T cells. **b** To evaluate the CD8^+^ signal at the tumor site, the operator-defined ROIs were used to demonstrate a 6.2- and 7.5-fold increase in the signal intensity in the tumor interior and periphery, respectively, during OX/IND-MSNP compared to saline treatment. The results are expressed as mean ± SEM. *p < 0.05; **p < 0.01, (ANOVA)

Immuno-positron emission tomography (immuno-PET) has been extensively employed in pre-clinical and clinical studies to noninvasively and quantitatively track the presence and abundance of CD8^+^ and other immune cell subsets following immunotherapy^46–48^. This technique is potentially useful to assess immunotherapy success before the treatment impact at the tumor site can be determined. To validate tumor-infiltration and systemic activation of CD8^+^ T cells, a ^89^Zr-desferrioxamine-labeled anti-CD8 cys-diabody (^89^Zr-malDFO-169 cDb) was used for monitoring. This PET probe has high specificity for tracking newly-induced CD8^+^ T cell responses^48, 49^. We asked whether PET imaging could show the induction of an effective anti-PDAC immune response in live animals, IV injected with saline, OX/LB-MSNP (5 mg/kg OX) or OX/IND-MSNP (5 mg/kg OX and 50 mg/kg IND). Treatment was administered to the animals (*n* = 3) on days 10, 14, 18, and 22 after orthotopic implantation. The ^89^Zr-probe (29–63 µCi) was IV injected on day 26 and microPET and CT scans were obtained, using a G8 PET/CT scanner (Sofie Biosciences), at 20 h. Coronal and transverse (Fig. 7a) view signal analysis for the localization of CD8^+^ T cells were obtained by AMIDE software. This demonstrated background levels of CD8^+^ T cells in a peripheral distribution in the tumors of saline-treated animals, accompanied by faint signals in the spleen and tumor draining lymph node (TDLN) (Fig. 7a, right panel). Since the PET probe is eliminated renally, the kidneys show intense radioactivity^48^. OX/LB-MSNP treatment was associated with a modest increases in radioactivity in the interior and peripheral tumor tissues, amounting to 2.5- and 3.1-fold increases, respectively (Fig. 7b). This was accompanied by increased radioactivity in the spleen and TDLN (Fig. 7a, Supplementary Fig. 14). In contrast, treatment with OX/IND-MSNP was accompanied by a prominent increase in the signal intensity in both the peripheral (7.5-fold) and interior (6.2-fold) tumor regions compared to saline. There was also a remarkable increase in signal intensity in the spleen and TDLN. All considered, immuno-PET confirms the generation of an effective systemic anti-PDAC immune response based on the synergistic effect of OX and IND-PL delivery.

## Discussion

PDAC is an often-fatal and treatment-resistant disease, in desperate need of new treatment approaches. We demonstrate three treatment modalities using ICD to generate an anti-PDAC immune response. The 1^st^ is a subcutaneous vaccination approach, which utilizes ex vivo induction of ICD by OX in a KPC cells to generate a systemic immune response that can interfere with tumor growth at a remote site, as well as allowing adoptive transfer to non-immune animals. The 2^nd^ treatment modality involved local injection of OX plus an IND-PL nanovesicle to induce the recruitment of cytotoxic CD8^+^ T  lymphocytes, depletion of Tregs, reversal of the CD8^+^/Foxp3^+^ ratio, cytotoxic tumor killing, and tumor shrinkage at the local injection site. These adaptive immune responses were accompanied by boosting of the innate immune system, as reflected by CRT and HMGB-1 expression, as well as the activation of DC population. The 3^rd^ treatment approach combined OX and IND-PL into a single MSNP-based nanocarrier, which allows systemic biodistribution and drug delivery to orthotopic KPC tumor sites. The dual-delivery approach achieved a synergistic anti-PDAC immune response, associated with a significant increase in animal survival. Strikingly, IND co-delivery had a significant impact on the ICD response, in addition to interference in the IDO pathway.

Our proposed nano-enabled approach for initiating immunotherapy offers distinct advantages over current immunotherapy strategies for PDAC, including peptide and protein vaccines^50^, whole-cell vaccination approaches^26^, DC vaccines^51^, microorganisms^52^ and immune checkpoint blockade (e.g., anti-CTLA-4 or anti-PD1 or monoclonal antibodies)^26^. Since most of these approaches rely on select antigens, the limited scope of the response fails to reflect the multitude of tumor antigens that may evolve during immune editing by the tumor. Moreover, the restricted display of antigenic epitopes to the T-cell antigen receptor (TCR) may not allow selection of receptors with optimal affinity or on/off binding constants for an effective response^53^. In contrast, ICD facilitates APC uptake and presentation of a full complement of tumor-associated antigens (mutagenic and non-mutagenic), which can effectively select the most effective TCRs, which are capable through receptor proofreading to provide the most effective instruction for cytotoxic killing. ICD could also allow the cognitive immune system to adapt to the array of continuously evolving tumor antigens rather than restricting the immune response only to the neo-antigens that are putatively required for the tumor immune response to checkpoint inhibitors.

The potential utility of ICD in an anti-PDAC immune response is reflected in studies using the whole-cell vaccine, Algenpantucel-L^26^. This vaccine is comprised of irradiated PDAC cells, genetically engineered to express the murine enzyme, α (1, 3)-galactosyltransferase (αGT)^26^. The expression of natural antibodies to αGal in the human host induces a hyper-acute immune response during vaccination with the PDAC cell lines. Their death is accompanied by ICD features^6, 15^. However, while the data from a phase II vaccine trial have demonstrated an antibody response to CRT and improved survival in PDAC patients, the outcome could not be reproduced in a phase III clinical trial^54^. This could be due to the limited range and short duration of tumor antigen presentation by the dying PDAC cells. In addition to PDAC, good experimental data have been provided to show the feasibility of ICD-inducing chemotherapy in lung or colon carcinoma, including further response amplification by immune checkpoint blockers^44, 54^. For colon cancer it has also been demonstrated that core-shell nanoparticles, comprised of an OX core and a photosensitizing pyrolipid shell conjugate, can synergize in delivering an abscopal effect^55^.

This is the 1^st^ report demonstrating the use of an ICD approach in PDAC through the use of nanocarriers. We also demonstrate the novelty of using a nanocarrier to generate a synergistic immune response by co-delivery of an ICD stimulus and interfering in immune suppression. The timeliness of using nanocarriers for dual drug delivery is confirmed by recent FDA approval of VYXEOS™, which provides synergistic daunorubicin and cytarabine co-delivery to patients suffering from acute myeloid leukemia^56^. The practical utility of our platform for initiating a synergistic immune response in PDAC is supported by the widespread use of OX as an ingredient of the FOLFIRINOX regimen. IND is also being used in PDAC clinical trials as a chemotherapy adjuvant.

Immune activation in the PDAC microenvironment has to overcome a number of immune suppressive mechanisms, including the presence of CD4^+^/Foxp3^+^ Tregs, secretion of anti-inflammatory cytokines, expression of checkpoint inhibitors and overproduction of IDO. While our results indicate that OX alone is capable of increasing the CD8^+^/Foxp3^+^ ratio at local and systemic tumor sites, the co-administration of a PL-conjugated IDO inhibitor, IND-PL, significantly enhanced the response parameter. This change reflects the importance of the IDO metabolic pathway in tumor immune surveillance, in much the same way as the regional IDO expression in the placenta plays a role in protecting the fetus^18^. Importantly, the delivery of IND in the form of a prodrug also impacts the innate immune system, as demonstrated by enhanced expression of CRT and HMGB-1 by the dual delivery carrier (Supplementary Figs. 7d, f, and 11c, e). This could reflect the effect of IND in promoting autophagy as a result of activation of the mTOR1 pathway. Autophagy plays a key role in ATP release during ICD^21^.

IDO inhibitors are currently undergoing clinical trials in several cancer types, including breast, prostate, melanoma, brain and pancreas^24^. This includes the use of IND together with gemcitabine, nab-PTX and anti-PDL1^24^. However, we have not observed ICD induction by gemcitabine or PTX in pancreatic cancer cell lines. A major advantage of our nanocarrier approach is the improvement of the PK and intratumoral concentration of IND-PL, along with OX. Free IND is relatively water insoluble and has unfavorable PK characteristics. In contrast, IND-NV significantly increased the uptake and release of IND in tumor cells (Fig. 3c); this also translated to a more robust interference in IDO-mediated immune suppressive signaling pathways at the tumor site (Figs. 3e, 6f, and Supplementary Fig. 6c). In addition to improving the circulatory t_1/2_ and PK of IND, the dual delivery carrier also improved the PK of OX (Fig. 5c and Supplementary Fig. 4i). Harmonization of their PKs contributed to synergy at the tumor site.

How can this discovery be translated to the clinic? On the basis of our animal studies, possible ways to improve immunotherapy in patients could include the following: (i) tumor cell collection from resected cancer tissues during surgery, with the possibility of developing a culture-based vaccine approach; (ii) local injection of OX and IND-PL into the tumor under remote guidance, during collection of biopsies or direct visualization during surgery; (iii) systemic administration of one or a combination of treatment modalities, which may include the use of free drugs, IND-NV or the dual-delivery carrier. In addition, it is also possible to enhance treatment efficacy by nanomaterials that exhibit catalytic properties that can be used for sequential induction of ER stress, ICD, autophagy and the release of adjuvants. It is also possible to use nanocarriers to deliver other FDA-approved drugs (e.g., cardiac glycosides, Ca^2+^-activated K-channel agonists, etc)^57^ to achieve ICD, individually or in combination with chemotherapy or ICD-inducing nanoparticles. Another approach could be to combine chemotherapy and IND delivering nanoparticles with immune checkpoint blockers, irradiation, photodynamic therapy, or cytotoxic viruses to achieve additional immune response amplification. The ultimate goal of a cure of PDAC through immunotherapy will likely require a series of steps and combination therapies.

In summary, we demonstrate that a nano-enabled approach for OX and IND delivery to the PDAC site can be used for a synergistic immunotherapy response premised on the induction of ICD plus reversal of IDO immune suppressive effects. The nano-enabled approach can be reduced to clinical practice by using a vaccination approach, local treatment or systemic administration. The same approach may also apply to other cancers.

## Methods

### Cells and mice

A KPC cell line, derived from a spontaneous tumor in a transgenic Kras^LSL-G12D/+^/ Trp53^LSL-R172H/+^/Pdx-1-Cre mouse, was used for the cellular studies and growing subcutaneous and orthotopic tumors in mice^25^. It was not logistically feasible to use the spontaneous mouse model because of the variability of tumor development, making it impossible to obtain enough mice for a comprehensive study. We also obtained a PANC-1 cell line from ATCC. Both cell lines were cultured in complete DMEM medium, containing 10% FBS, 100 U/mL penicillin, 100 μg/mL streptomycin, and 2 mM L-glutamine. All cell lines were tested to ensure freedom from mycoplasma contamination. To visualize KPC tumor growth by IVIS bioluminescence imaging, the KPC cells were stably transfected with a luciferase-expressing lentiviral vector in the vector core facility at UCLA^4^. Female B6/129 mice (Jackson Laboratory, 8~ 10 weeks old) were used to grow subcutaneous or orthotopic KPC tumors. The animals were maintained under pathogen-free conditions and all animal experiments were approved by the UCLA Animal Research Committee.

### CRT expression and HMGB-1 release from the cell lines

1 × 10^5^ KPC or PANC-1 cells were seeded in 24-well plates overnight. The cell culture medium was removed and replenished with Cis, OX and DOX containing media at the indicated concentrations for 4 h or 24 h. Supernatants were collected for HMGB-1 detection by an ELISA kit (IBL International GmbH), according to the manufacturer’s instructions. To assess CRT expression by flow cytometry, cells were trypzinized, washed in cold PBS and then sequentially stained with a primary rabbit anti-CRT antibody (Ab2907, Abcam), followed by an Alexa Fluor^®^ 680-conjugated goat-anti-rabbit IgG antibody for 30 min at 4 °C. The cells were incubated in 500 µL PBS containing 50 µg/mL propodium iodide before washing and assessment in a LSRII flow cytometer (BD Biosciences). The data were expressed as fold-increase in mean fluorescence intensity (MFI) compared to the PBS control. The analysis was repeated once. Visualization of CRT expression was performed in KPC cells added to 8-well chamber slides (Lab-Tek^®^). Each well contained 1 × 10^4^ KPC cells in 0.4 mL of culture medium. After incubation with 50 µM Cis, 50 µM OX, and 1 µM DOX for 4 h, cells were fixed and washed 3 times. Cells were stained with an Alexa Fluor^®^ 647-conjugated anti-CRT antibody (ab196159, 1/500, Abcam) for 30 min, followed by co-staining with 5 μg/mL Alexa Fluor^®^ 488-conjugated wheat germ agglutinin (WGA) to visualize the cell surface membrane. Slides were mounted with Hoechst 33,342 nuclear dye and visualized under a Leica SP8-SMD confocal microscope. High magnification images were obtained under the 63 × objective lens.

### Vaccination approach to induce systemic immunity

The timeline for the vaccination schedule is described in Fig. 2c. KPC cells were exposed to PBS, 100 µM Cis, 50 μM OX and 1 μM DOX for 24 h to induce CRT expression. After confirmation of CRT expression by flow cytometry, 1 × 10^6^ dying cells were injected twice into the right flank of B16/129 mice (*n* = 7), 7 days apart. 14 days after the 1^st^ injection, the animals received SC injection of viable KPC cell suspensions (1 × 10^6^ cells in 0.1 mL DMEM/matrigel, 1/1, v/v) in the contralateral (left) flank. Tumor size was measured by a digital caliper every 3–4 days, and the volume calculated according to the formula π/6 × length × width^2^. Tumor burden was also monitored by IVIS imaging on day 7, 18, 25, and 29 and quantitatively expressed as luminescence signal intensity in the region of interest (ROI). The data were present as “spaghetti plots” that display the tumor growth in each individual animal. Statistical comparison of the groups was performed using two-way analysis of variance with SPSS software. Animals were killed on day 29 and the tumors were collected for flow cytometry and IHC analysis as described below.

### Orthotopic rechallange and adoptive transfer

In order to demonstrate immune memory, surviving mice from the vaccination study were used for this experiment. Three tumor-free survivors in the OX group and 3 healthy mice were used for secondary tumor challenge by orthotopic pancreatic implant on day 74. This was accomplished by injecting 1 × 10^6^ live KPC-luc cells into the pancreas after minor surgery^4^. Tumor development was monitored by IVIS imaging. While the healthy animals developed pancreatic tumors, the animals in the OX-treated group remained tumor-free. After killing of the survivors and collecting their splenocytes on day 132, adoptive transfer was performed to non-immune B16/129 recipients (*n* = 6). This was accomplished by injecting 3 × 10^6^ splenocytes IV. The controls consisted of 6 non-immunized animals injected with splenocytes from non-immune animals or 6 animals injected with splenocytes from saline-treated animals. Two days later, each of the groups was challenged by injection of 2 × 10^5^ viable KPC cells SC. To confirm the tumor specificity, 3 identical injected animal groups were used for SC challenge with B16 melanoma cells.

### Synthesis of the IND-PL prodrug

The procedure was carried out in 3 steps, the 1^st^ of which was “synthesis of Boc-IND”. IND (200 mg), Di-*tert*-butyl dicarbonate (Boc anhydride, 260 mg) and NaHCO_3_ (230 mg) were dissolved in a mixture containing 10 mL tetrahydrofuran (THF) and 10 mL H_2_O. The sample was stirred at 0 °C for 15 min and then at room temperature overnight. THF was removed by evaporation, followed by the addition of 1 N HCl (10 mL). The solution was brought to pH = 1 by crystal precipitation, followed by suction filtration to purify the pale-yellow solid. The molar ratio of the product vs. starting materials was used to determine the yield in each step (Supplementary Fig. 4a). Synthesis success  was confirmed by ^1^H-NMR, ^14^C-NMR and ESI-MS (positive mode), as described online. Subsequent synthesis of Boc-IND-PL was performed by dissolving 100 mg 1-palmitoyl-2-hydroxy-sn-glycero-3-phosphocholine (PL), 150 mg Boc-IND, 156.7 mg EDC, 97.3 mg DMAP, and 146 mg DIPEA in water-free dichloromethane (DCM, 20 mL), while stirring for 48 h. The resulting pale-yellow solution was obtained by funnel separation (repeated 3 times, using water). The DCM solution was vacuum-dried and purified by silica-gel chromatography, using a mobile phase comprised of ethanol:chloroform:water (4:6:1, v/v/v). Analysis of the yield, and characterization of the product was performed by NMRs and ESI-MS, as described online (Supplementary Fig. 4). In the final step, the synthesis of IND-PL was carried out by stirring 58.6 mg Boc-IND-PL in a mixture of 1 mL trifluoroacetic acid and 1 mL DCM for 6 h at room temperature. The solvent was removed by rotatory evaporation and the residue was re-dissolved in 400 µL DCM, to which 25 mL diethyl ether was added dropwise, followed by centrifugation to retrieve the pale-yellow solid. The washing step was repeated thrice using diethyl ether. The final product was comprehensively characterized for its purity and composition by NMRs and ESI-MS.

### Self-assembly of IND-PL into INV-NV nanovesicles

The self-assembly of IND-PL into IND-NV was carried out by a slight variation of a liposome synthesis procedure. Briefly, 5 mg of IND-PL was dissolved in chloroform in a 50 mL round bottom glass flask. The solvent was evaporated under a rotatory vacuum to form a thin film, which was dried further under vacuum overnight. The film was hydrated with 1 mL of PBS and sonicated for 1 h. To obtain size-controlled IND-NV assembly, the suspension was extruded 13 times through a Mini-Extruder (Avanti Polar Lipids), using a polycarbonate membrane with 100 nm pores (Avanti Polar Lipids) at 80 °C. IND-NV size and morphology were assessed by dynamic light scattering and cryoEM, respectively (Fig. 3b).

### Cellular uptake of IND-NV

KPC cells were treated with free IND or IND-NV at the IND dose-equivalent of 100 µg/mL for 4, 24, and 72 h, respectively. This dosing is based on literature.^20^ The cells were detached by trypsinization and extracted overnight in methanol to determine drug content by UPLC-MS/MS analysis. These extracts were added to a C18 Column (130 Å, 1.7 µm, 2.1 mm × 50 mm), connected to Waters LCT Premier with ACQUITY UPLC and Auto sampler. The separation involved gradient elution as follows: (i) 0–4.5 min, 95% water + 5% Acetonitrile; (ii) 4.5–6 min, 5% water + 95% Acetonitrile; and (iii) 6–10 min, 95% water + 5% Acetonitrile. The flow rate was 0.4 L/min. Since IND is a small molecule that may diffuse from trypsinized cells, with the possibility of impacting intracellular drug analysis, we also performed the experiment by washing the pre-chilled monolayer with ice-cold buffer and then extracting in situ before UPLC-MS/MS analysis.

### Effect of IND-NVs on immunoregulatory signaling pathways

1 × 10^6^ KPC cells were seeded into each well of a 6-well plate overnight, using Trp-deficient DMEM (Gibco)^21^. After attachment, the cells were treated with IND or IND-NV at the indicated concentrations for 3 h. The supernatants were collected to assess IL-6 levels by an ELISA kit (BD Biosciences) according to the manufacturer’s instructions. In order to determine the abundance of P-S6, total S6K and GAPDH by western blotting, cells were extracted in a lysis buffer, followed by electrophoresis on a 4–12% SDS-PAGE gel (Invitrogen, Grand Island, NY). The proteins were subsequently transferred to a PVDF membrane. After blocking in 5% BSA, the membrane was sequentially overlaid with primary and secondary antibodies and the blots developed by the addition of the ECL solution. The original uncropped scans of the blot was shown in Supplementary Fig. 5e. The band intensity on the film was quantified by Image J software.

### Local tumor injection of free OX plus IND-NV

1 × 10^6^ KPC cells (in 100 µL DMEM/matrigel, 1/1, v/v) were SC injected in the right flank of the animals (*n* = 7). The dose design is based on literature^15^. When tumors reached 60–80 mm^3^ in size, the mice received one-time IT administration of saline, free OX (1.25 mg/kg), free OX (1.25 mg/kg) + free IND (2.5 mg IND/kg), free OX (1.25 mg/kg) + free IND (12.5 mg/kg IND), free OX (1.25 mg/kg) + IND-NV (2.5 mg/kg IND) and free OX (1.25 mg/kg) + IND-NV (12.5 mg IND/kg). We also included a single IT injection of free IND (12.5 mg IND/kg) and IND-NV (12.5 mg IND/kg) as additional controls. Tumor burden was measured by a digital caliper, similar to what was described in the vaccination experiment. On day 31, tumors were collected for flow cytometry and IHC analysis as described above.

### Synthesis of the OX/IND-MSNP nanocarrier

An OX/IND-MSNP dual-delivery carrier was designed by trapping OX in the mesoporous interior of a ~ 70 nm MSNP, using a lipid bilayer into which IND-PL was incorporated. Particle coating used a biofilm method that rapidly encapsulates the MSNP core during energy input^4, 5^. This was accomplished by adding the OX-soaked MSNP suspension on top of a lipid biofilm. For OX loading into the pores of MSNP, 30 mg of OX was dissolved in 30 mg of a MSNP solution (4 mL) while stirring overnight. The mixture was centrifuged at 15000 rpm 3 times for 15 min to remove the non-encapsulated OX and then resuspended in 3 mL DI water. The biofilm was comprised of a 60 mg lipid mixture, which included IND-PL, cholesterol, and DSPE-PEG_2K_ in the molar ratio of 75:20:5. This ratio was derived by experimenting with different lipid mixtures (Supplementary Fig. 8a). The lipids were dissolved in chloroform at a concentration of 20 mg/ml, and added to a round bottom flask. Following rotary vacuum evaporation of the chloroform, the OX-soaked MSNP suspension was added to the uniformly dispersed lipid biofilm, and then sonicated with a probe sonicator for 1 h, using a 15/15 s on/off working cycle at a power output of 32.5 W. Then drug-loaded particles were washed 3 times by centrifugation at 15,000 rpm for 15 min to remove free liposomes, and resuspended in DI water, saline, or PBS, as indicated. The purified OX/IND-MSNPs were fully characterized for size, charge, loading capacity, morphology and endotoxin level using DLS, UPLC-MS/MS, ICP-OES, cryoEM and the Chromogenic LAL Assay, respectively. An optimal particle batch was comprised of particles with size around 100 nm, slightly negative charge and suspension stability of at least one month. Control particles were synthesized by entrapping OX only inside the particle with a lipid bilayer of the same composition, except for using DSPC in place of IND-PL to yield OX/LB-MSNP (DSPC/cholesterol/DSPE-PEG_2K_ = 75:20:5, molar ratio in lipid bilayer). Particles were stored at 4 °C prior to use in cellular and animal experiments.

### PK study of IV-injected OX/IND-MSNP

Orthotopic tumor-bearing mice were used in this experiment (*n* = 6). To visualize OX/IND-MSNP nanoparticle biodistribution in vivo, NIR-labeled OX/IND-MSNP was prepared by incorporating 0.1% w/w Dylight 680-labeled DMPE in the lipid biofilm^4^. For IVIS bioluminescence imaging of the tumor site, mice were injected intraperitoneally (IP) with 75 mg/kg D-Luciferin. Reference fluorescence images for the tumor-bearing mice were acquired prior to particle injection (0 h). Following a single IV injection of NIR-labeled OX/IND-MSNP, delivering the equivalent of 5 mg/kg OX and 50 mg/kg IND, mice were imaged at 2.5, 8, 24, and 48 h post injection. After killing, ex vivo images were obtained for the collected tumor, heart, liver, spleen, kidney, and lung tissues at 24 h and 48 h. In a separate experiment, OX/IND-MSNP (5 mg/kg OX; 50 mg/kg IND) was IV administered to orthotopic KPC tumor-bearing mice (*n* = 6). Free OX served as a control. At the indicated time points (0.083, 2, 8, 24, and 48 h) plasma was collected and digested in methanol or HNO_3_/H_2_O_2_ for UPLC-MS/MS (to measure IND&IND-PL) or to perform ICP-OES (for Si elemental analysis), respectively. The use of 5 times reflect the limitation of not withdrawing a total of >250 µL blood and requirement of 50 µL for each assay. The *t*_1/2_ was calculated based on a non-compartmental model, using WinNolin software^58–60^. The collected tumor tissue and organs were also used to measure the drug and Si contents using similar methods.

### Efficacy assessment of the dual delivery OX/IND-MSNP carrier

We utilized a National Cancer Institute protocol^44, 61^ to calculate the maximum tolerated dose (MTD) of IV-injected free and encapsulated OX; this was determined to be 8 and 12 mg/kg, respectively. Briefly, two healthy male b6/129 mice were IV injected with a 2.5 mg/kg (C1 dose) equivalent of free or encapsulated OX. This was followed by a dose escalation factor of 1.8 to obtain the C*n* dose, which is followed by animal death within 24 h after the last administration. The second round dose-seeking began with the C*n*−1 dose and utilized a 1.15 escalation factor (*n* = 2) to determine the MTD, which is defined as the absence of mortality or morbidity. The MTD was validated by injecting six mice, which were monitored for 15 days to evaluate the absence of morbidity, mortality, or >15% weight loss. The corresponding MTD value for IV-injected IND-NV was >500 mg/kg. Orthotopic tumor-bearing B6/129 mice (*n* = 7) were randomly assigned to 9 groups, each including seven animals. The mice were IV injected with OX/IND-MSNP to deliver a dose of 5 mg/kg OX and 50 mg/kg IND every 4 days from day 10 onwards. The controls included equivalent doses of free OX, OX-laden particles only, IND-NV, IND-NV + free OX, delivered at the same administration frequency. We also included saline as a negative control. Tumor burden was monitored by IVIS imaging from day 10 onwards, allowing the bioluminescence imaging intensity to be quantitatively expressed in the operator-defined ROI. The tumor tissue and major organs were collected for quantification of ex vivo bioluminescence image intensity. On day 36, tumors were collected for flow cytometry, IHC analysis and determination of mRNA expression (for P-S6K, AHR, and IL-6) by real-time PCR. We also withdrew blood for blood chemistry analysis. In order to assess the adaptive and innate immune changes leading to effective anti-PDAC immunity during OX/IND-MSNP treatment, we also asked whether dosing with anti-CD8 and anti-TLR4 antibodies or knockdown of CD91 by siRNA could change the outcome (Supplementary Fig. 12). The experiment was repeated once, using the same treatment comparisons, to determine the effect on survival rate in each group (*n* = 7). Kaplan–Meier plots were used to express animal survival.

### Immunohistochemistry analysis

In order to visualize the phenotypic changes during the induction of innate and cognate immune response, IHC analysis was performed. Tumors collected from the killed animals were evenly divided into two parts, one for IHC and the other for flow cytometry. To prepare the tumor samples for IHC staining, the tumor pieces were fixed in 10% formalin followed by paraffin embedding. Tumor sections of 4 μm thickness were mounted on glass slides by the UCLA Jonsson Comprehensive Cancer Center Translational Pathology Core Laboratory for hematoxylin-eosin (H&E) staining as well as a series of IHC staining procedures, following standardized protocols. Briefly, the slides were deparaffinized, incubated in 3% methanol-hydrogen peroxide, followed by 10 mM EDTA (pH = 8) or 1 mM sodium citrate (pH = 6) at 95 °C using the Decloaking NxGen Chamber (Biocare Medical, DC2012). The slides were brought to room temperature, rinsed in PBST (Phosphate Buffered Saline containing 0.05% Tween-20) and then incubated with individual primary antibodies for 1 h. The slides were rinsed with PBST and then incubated with appropriate HRP-conjugated secondary antibodies at room temperature for 30 min. After rinsing with PBST, the slides were incubated with DAB (3,3′-Diaminobenzidine) or Vulcan Fast Red Chromogen Kit 2 (for the CRT and CD91/LRP1 protocols only) (Biocare Medical, FR805) for visualization. Subsequently, the slides were washed in tap water, counterstained with Harris’ Hematoxylin, dehydrated in ethanol, and mounted with media. The slides were scanned by an Aperio AT Turbo Digital Pathology Scanner (Leica Biosystems) and interpreted by an experienced veterinary pathologist.

### Antibody sources used for IHC

Primary antibody sources and dilutions (2% BSA) obtained from Abcam included: anti-CD4 (ab183685, 1/200), anti-CRT (ab2907, 1/50), anti-HMGB-1 (ab18256, 1/200), anti-LRP1(CD91) (ab92544, 1/50), anti-TLR4 (ab13867, 1/50), and anti-perforin (ab16074, 1/100). Anti-CD8 (#14-0808, 1/100), anti-Foxp3 (#13-5773, 1/200) and anti-IL-10 (#14-7101, 1/50) were from eBioscience. Anti-cleaved caspase-3 antibody was from Cell Signaling (#9664, 1/200) and anti-IFN-gamma from Novus Biologicals (NBP1-19761, 1/200). Anti-IL-12p70 was purchased from Novus Biologics (NBP1-85564, 1/100), and anti-IDO was from Biolegend (#122402, 1/100) Secondary antibodies included MACH2 Rabbit HRP-Polymer (Biocare Medical, RHRP520L) for IL-10 and TLR4; MACH2 Rabbit AP-Polymer (Biocare Medical, RALP525) for CD91; Dako EnVision + System HRP-labeled polymer Anti-Rabbit (Dako, K4003) for the remaining biomarkers.

### Flow cytometry analysis

The tumor pieces obtained for single-cell analysis were cut into smaller pieces with scissors and digested in DMEM with 0.5 mg/mL collagenase type I (Worthington Biochemical Corporation) at 37 °C for 1 h. The digested tissues were gently meshed though a 70 μM cell strainer, twice. Red blood cells were lysed by Ack lysing buffer (Gibco) according to the manufacturer’s instructions. The single-cell suspensions were washed twice and resuspended in staining buffer. Following cell counting and aliquoting, the suspensions were incubated with FcBlock (TruStain fcX^TM^ anti-mouse CD16/32, clone 93, BioLegend) for 20 min to avoid nonspecific binding. Staining was then performed by using various combinations of fluorophore-conjugated antibodies for 40 min at 4 °C. The following anti-mouse antibodies were purchased from BD Biosciences: CD45-V450 (#560501, 1/100), CD45-APC-Cy7 (#557659, 1/100), CD4-Alexa Fluor488 (#557667, 1/100), Foxp3-PE (#563101, 1/100), CD8α-PE (#561095, 1/100), CD11b-PE (553311, 1/100), CD11c-V450 (560521, 1/100). CD284 (TLR4)-APC (145406, 1/100) and CD103-Alexa Fluor 647 (#121410, 1/250) was purchased from BioLegend. LRP1 (CD91)-Alexa fluor 647 (ab195568, 1/250) was obtained from Abcam. CD3-APC-eFluor780 (#47-0032-82, 1/100) and CD25-APC (#17-0251-82, 1/100) were purchased from eBiosciences. Multi-parameter staining was used to identify the following populations of interest: (i) CD8^+^ T cells (CD45^+^CD3^+^CD8^+^CD25^+^), (ii) Tregs (CD45^+^CD3^+^CD4^+^Foxp3^+^), (iii) CD91^+^ DCs (CD45^+^CD11b^+^CD11^+^cCD91^+^), (iv) TLR4^+^ DCs (CD45^+^CD11b^+^CD11^+^cTLR4^+^), and (v) CD103^+^ DCs (CD45^+^CD11b^+^CD11^+^cCD103^+^). For intracellular Foxp3 staining, cells were further fixed and permeabilized using a Foxp3/Transcription Factor Staining Buffer Set (eBioscience). After washing, cells were used for flow cytometry analysis (machine brand name: LSRII, BD Biosciences). The data were processed by FlowJo software (Tree Star). Dead cells and doublets were excluded based on forward and side scatter.

### Immuno-PET imaging

Immuno-PET imaging was used to assess systemic immune activation in live animals., MalDFO-conjugated anti-CD8 cDb fragment was incubated for 1 h at room temperature at about 4–5 µCi ^89^Zr per µg protein^48, 49^. Radiolabeling efficiency was measured by ITLC (Biodex Medical Systems) using 20 mM citrate buffer pH 5.6 as the mobile phase. The ITLC strip was cut in half and sections were counted using a Wizard 3″ 1480 Automatic Gamma Counter (Perkin-Elmer). Protein was purified using BioRad6 Spin columns equilibrated with PBS. Radiochemical purity was assessed by ITLC as above. Nine KPC orthotopic mice were established as described earlier. Saline, OX/LB-MSNP (5 mg OX/kg), and OX/IND-MSNP (5 mg OX/kg and 50 mg IND/kg) were IV injected to mice (*n* = 3) on day 10, 14, 18, and 22 for 4 consecutive administration post KPC tumor cells inoculation into pancreas. At day 26, 100 µL doses containing 1.07–2.33 MBq (29–63 µCi, 2.3–5.3 µCi/µg) ^89^Zr radiolabeled cDb PET probe in saline was IV injected to orthotopic KPC-tumor-bearing mice. 20 h later, mice were anesthetized and microPET and microCT scans were acquired using a G8 PET/CT scanner (Sofie Biosciences) in CNSI. MicroPET images were reconstructed by non-attenuation or scatter corrected maximum a posteriori (MAP) reconstruction. Images including coronal and transverse views were acquired and analyzed by AMIDE (a software for viewing, analyzing, and registering the volumetric PET imaging data).

### Statistical analysis

Statistical analysis was carried out with the SPSS statistical package (version 23, SPSS). Differences between groups were analyzed using analysis of variance (ANOVA). Comparison of Kaplan–Meier survival curves was performed with the Log-rank Mantel–Cox test. The results were expressed as mean ± SEM of at least three independent experiments. Statistical significance thresholds were set at *p < 0.05; **p < 0.01; ^#^ *p* < 0.001.

### Data availability

The data that support the findings of this study are available within this article and its Supplementary Information or from the corresponding author upon reasonable request.

## Electronic supplementary material

Supplementary Information
