# Supplementary material for: Nano-enabled pancreas cancer immunotherapy using immunogenic cell death and reversing immunosuppression
Source: Nat Commun. 2017 Nov 27;8:1811. doi: 10.1038/s41467-017-01651-9 (PMC5703845; doi:10.1038/s41467-017-01651-9)
Supplement: Supplementary file 1 — Supplementary Information [file 41467_2017_1651_MOESM1_ESM.pdf]

Supplemental Table and Figures

Supplementary Table 1. Abbreviation list.

Ctr	Control
Cis	Cisplatin
OX	Oxaliplatin
DOX	Doxorubicin
IND	Indoximod
PL	1-palmitoyl-2-hydroxy-sn-glycero-3-phosphocholine
IND-PL	1-palmitoyl-2-hydroxy-sn-glycero-3-phosphocholine conjugated Indoximod
IND-NV	Self-assembled nanovesicles that is formed when adding IND-PL into aqueous solution
CRT	Calreticulin
HMGB-1	High mobility growth box 1 protein
OX/LB-MSNP	OX-laden lipid coated mesoporous silica nanoparticles
OX/IND-MSNP	OX-laden Ind-PL coated dual delivery mesoporous silica nanoparticles
RT	Retention time
ALT	Alanine aminotransferase
AST	Aspartate aminotransferase
ALP	Alkaline phosphatase

Supplementary Figure 1. (**a**) Flow cytometry analysis to show the normalized CRT expression levels in KPC cells exposed to the indicated concentrations of Cis, OX, and DOX for 24 h. (**b**) Similar flow cytometry analysis in PANC-1 cells treated for 4 or 24 h. (**c**) ELISA measurement of HMGB-1 release from KPC and PANC-1 cells after treatment with Cis, OX, and DOX for 4 h. *$p < 0.05$; **$p < 0.01$, (ANOVA) compared to 0 μM

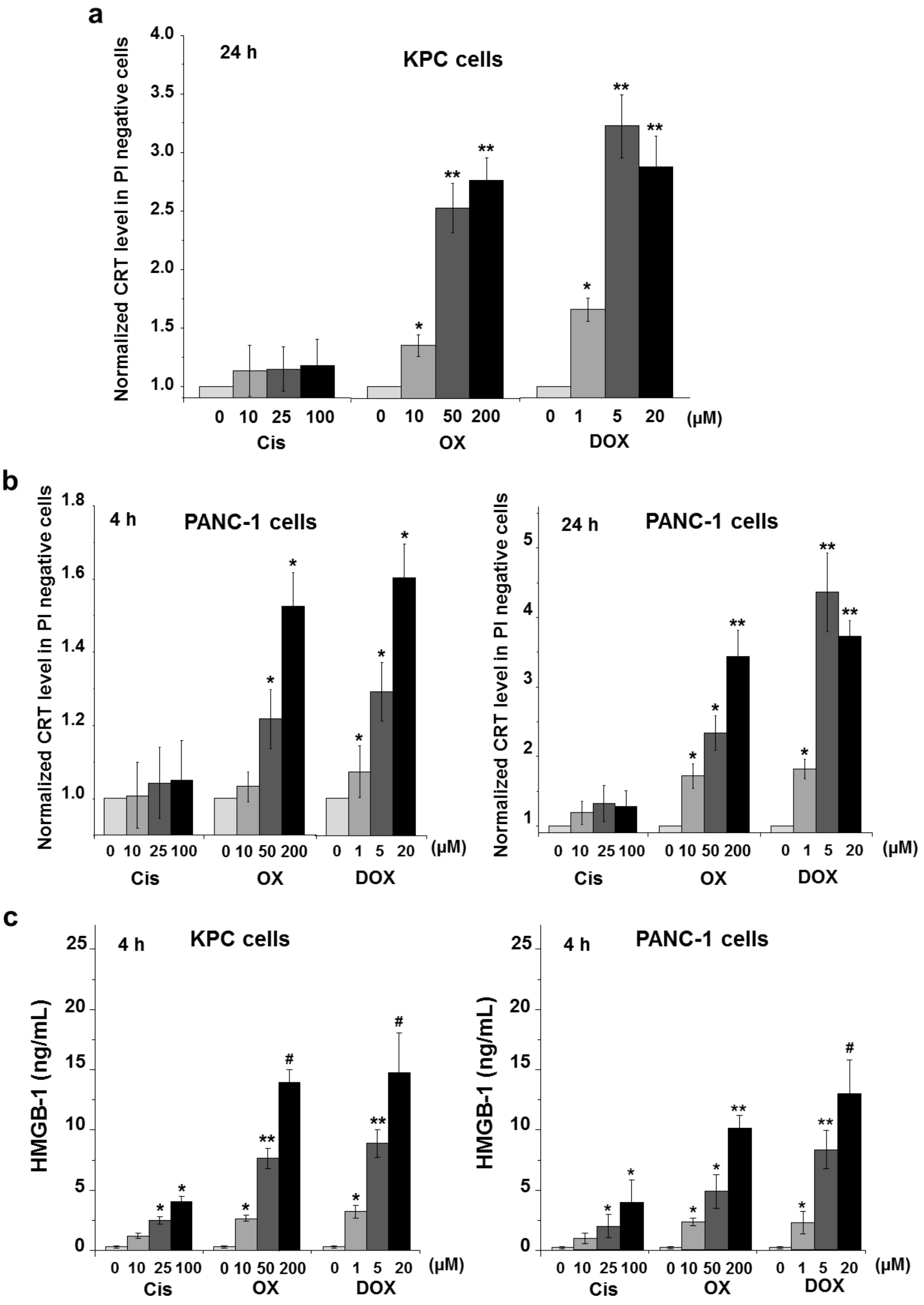

Supplementary Figure 2. (**a**) IVIS optical imaging to follow the tumor burden in the vaccination experiment, as described in the manuscript in **Fig. 2d**. (**b**) Monitoring of animal weight in the vaccination experiment. (**c**) IHC analysis to discern CD4 expression in harvested tumor tissue on day 29. Scale bar is100 μm.

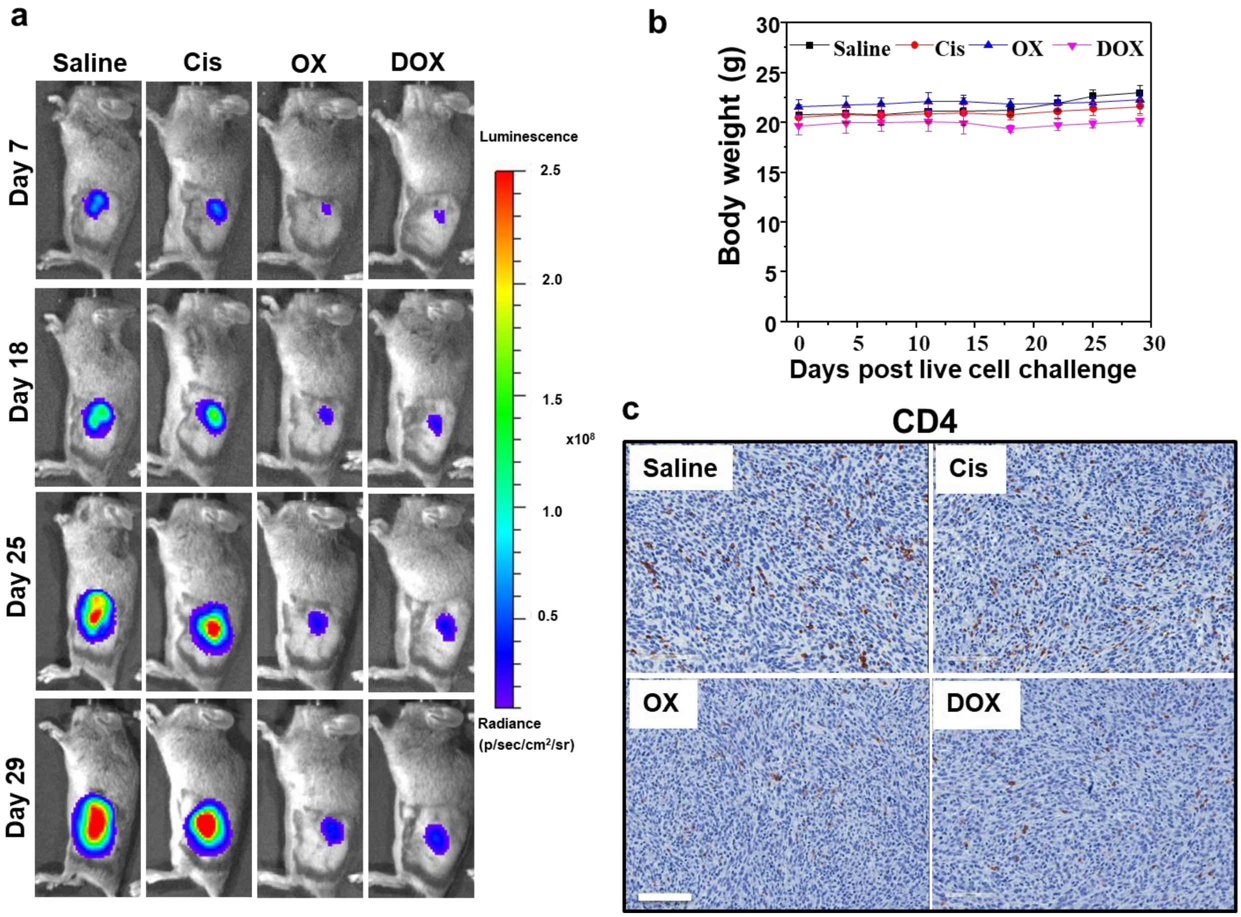

Supplementary Figure 3. Adoptive transfer experiment in which the recipient mice are challenged by SC injection of the B16 melanoma cell line, after receiving IV injection of immune and non-immune splenocyte populations, as described in **Fig. 2g** in the manuscript. There was no statistical significance in the growth rate of the 3 different animal groups (n = 6), demonstrating the antigen-specific nature of the anti-PDAC immune response that was not protective against melanoma.

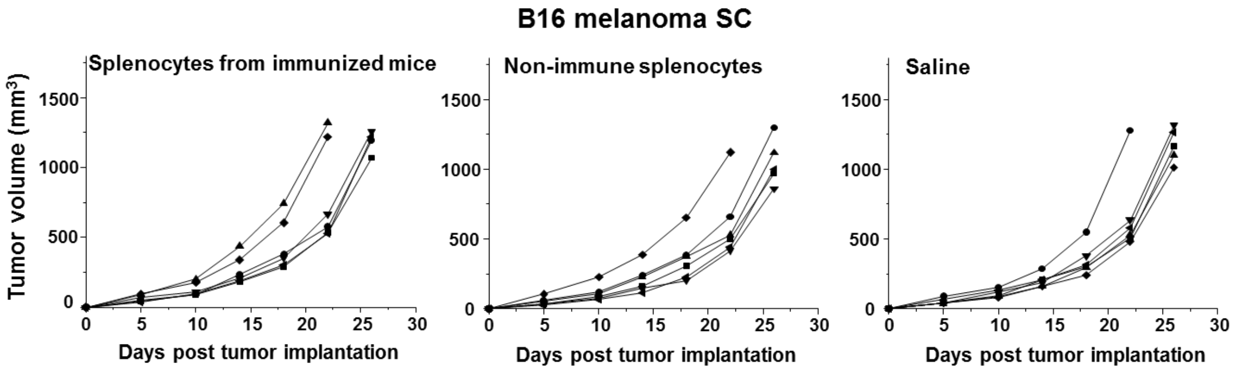

Supplementary Figure 4. Detailed characterization of the intermediary products during IND-PL synthesis, as shown in **Fig. 3a** of the manuscript. Table (**a**) summarizes the intermediates and show the yield at each stage of the synthesis process. (**b, c**) ESI-MS, ^{1}H-NMR and ^{13}C-NMR data for Boc-IND. (**d, e**) Boc-IND-PL analysis by ESI-MS, ^{1}H-NMR and ^{13}C-NMR. (**f, g**) IND-PL analysis by ESI-MS, ^{1}H-NMR and ^{13}C-NMR. Collectively, the comprehensive characterization confirms successful IND-PL synthesis. (**h**) Low magnification cryoEM image of IND-NV. (**i**) The unfavorable PK of free IND, as demonstrated by the short half-life and low tumor retention capability, highlights the necessity of a nano-enabled approach to improve retention of the IDO inhibitor.

**a**	***Starting material 1***	***Starting material 2***	***Catalytic materials/ Reaction solution***	***Product***	***Yield***
**Step 1**	Indoximod (IND) (200 mg)	Di-tert-butyl dicarbonate (Boc anhydride) (260 mg)	$NaHCO_3$ (230 mg)<br>THF (10 mL)<br>H_2O (10 mL)	Boc-IND (272 mg)	93 %
**Step 2**	PL (100 mg)	Boc-IND (150 mg)	EDC (156.7 mg)<br>DMAP (97.3 mg)<br>DIPEA (146 mg)<br>Dry DCM (20 mL)	Boc-IND-PL (58.6 mg)	37%
**Step 3**	Boc-IND-PL (58.6 mg)	N/A	TFA (1 mL)<br>Dry DCM (1 mL)	IND-PL 32.4 (mg)	63 %

b **Boc-IND**

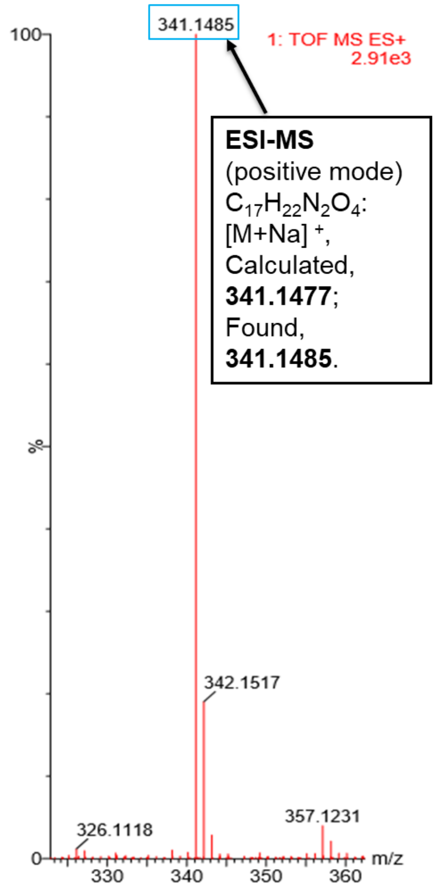

c

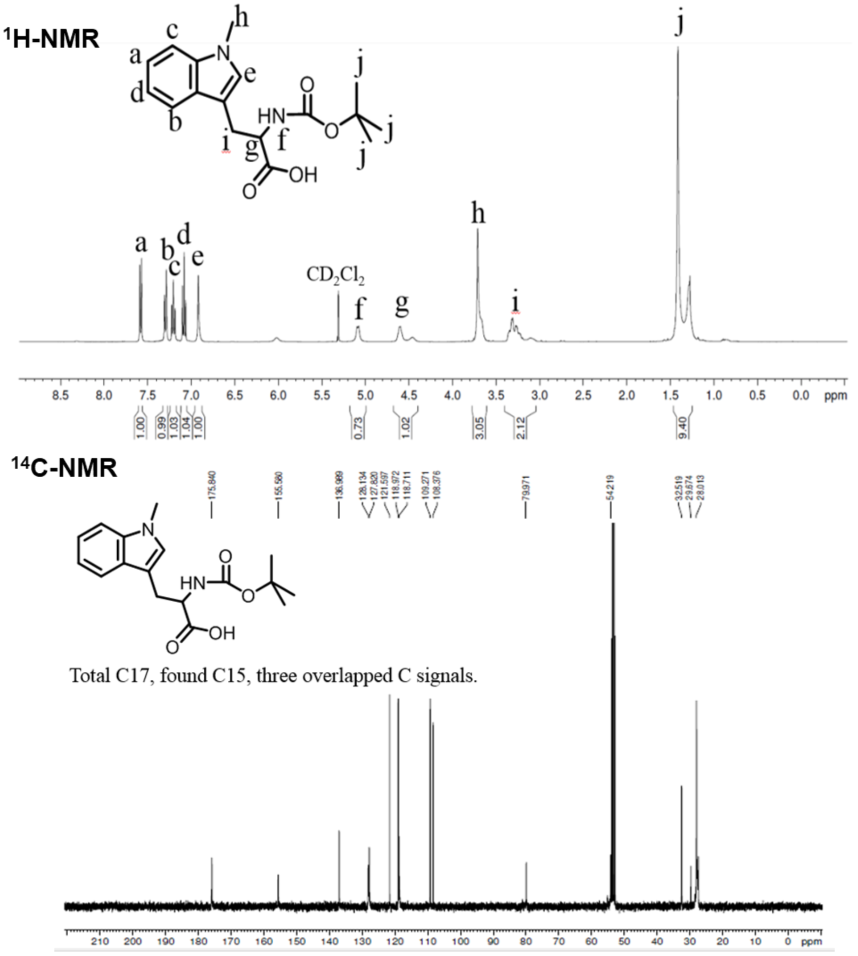

d Boc-IND-PL

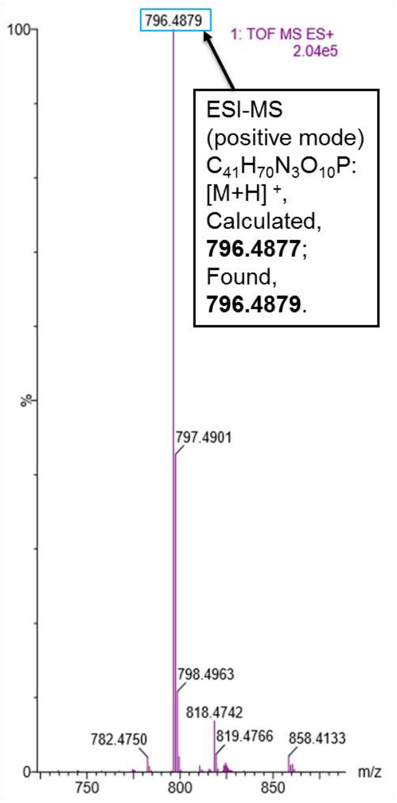

e

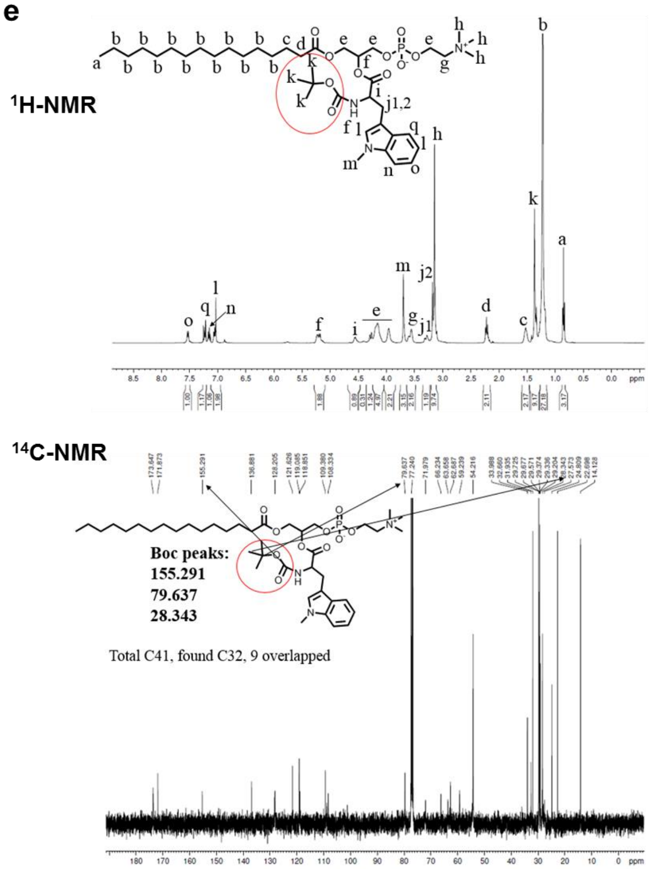

f IND-PL (final product)

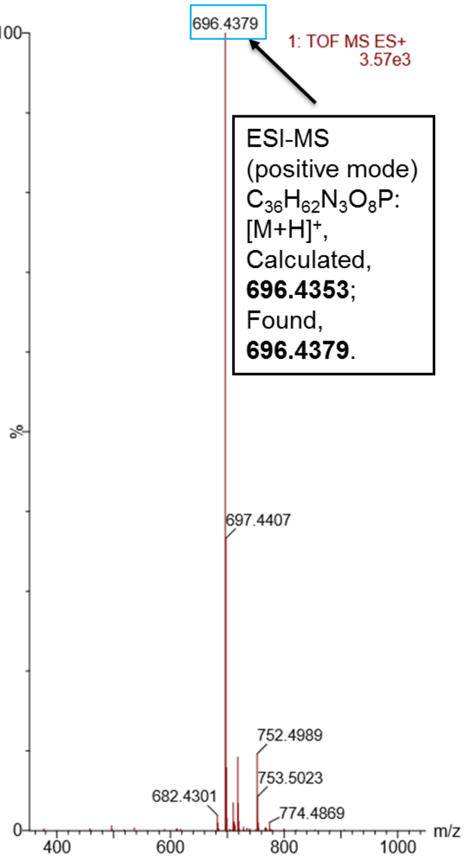

g

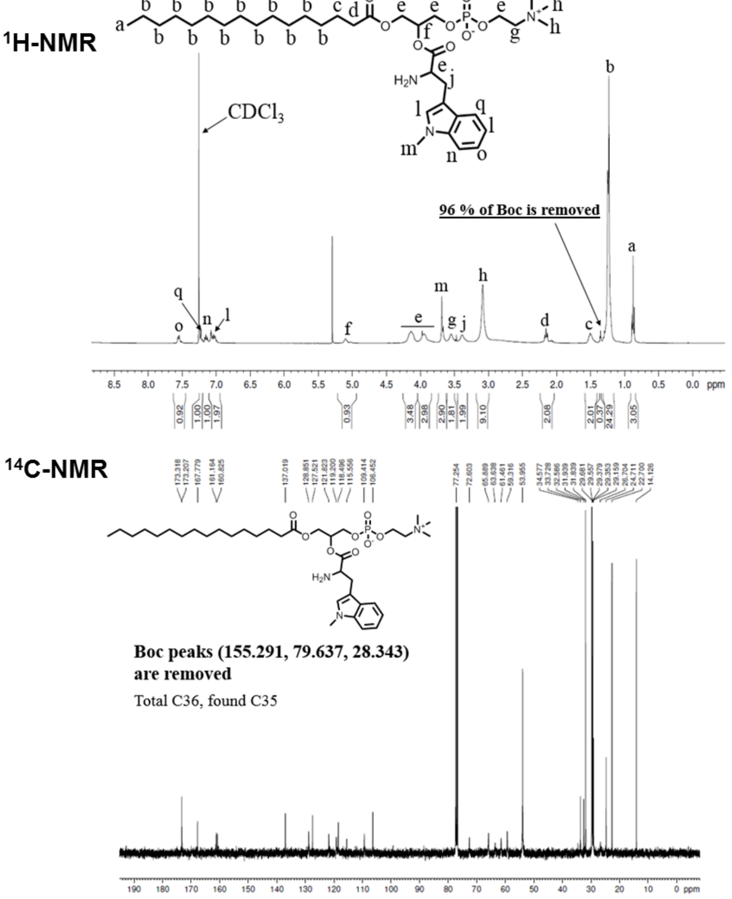

h

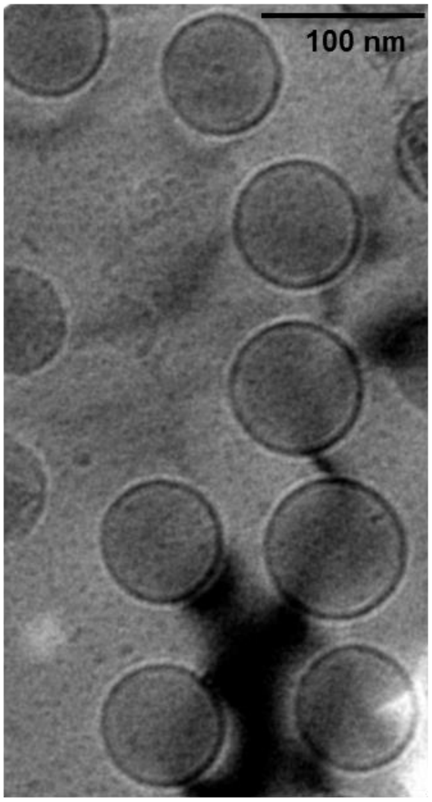
100 nm

i

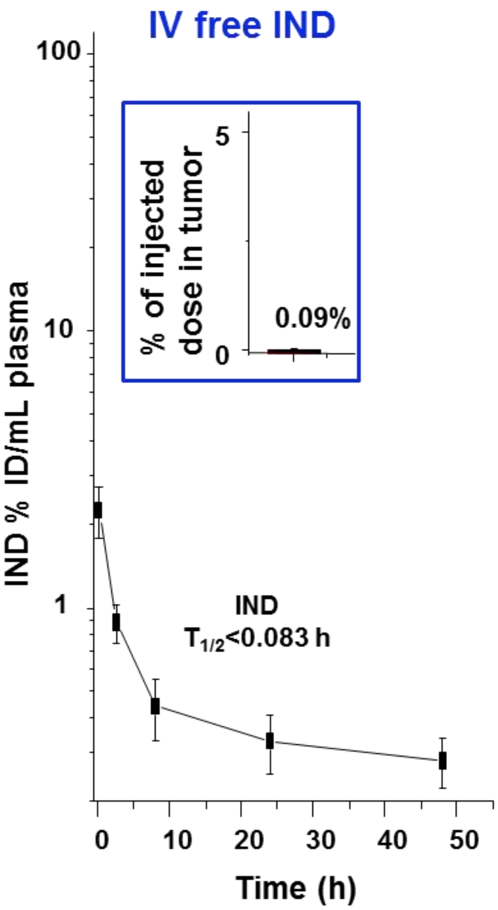
IV free IND
100
10
1
IND % ID/mL plasma
% of injected dose in tumor
5
0
0.09%
IND
$T_{1/2}$<0.083 h
0
10
20
30
40
50
Time (h)

Supplementary Figure 5 (**a**). UPLC-MS/MS analysis of the cellular content and intracellular release of IND from IND-NV. (**b**) Establishment of distinct retention times for IND (1.23 min), PL (3.97 min), and IND-PL (3.08 min) allowed us to use UPLC analysis to demonstrate the total and released drug content in the cells, as described in **Fig. 3c** in the manuscript. (**c**) An abiotic experiment was performed to demonstrate that acidic pH and esterase activity can release IND from the prodrug. Briefly, 2 mg/mL of IND-NV was dissolved in 2 mL PBS (pH=7.4), acidified buffer (pH 5.0 and 6), or 100 μM esterase in PBS, while vortexing at 37 °C. Solutions were centrifuged and the drug precipitates were dissolved in methanol for overnight before UPLC-MS/MS analysis after 0, 1, 6, 12, 24, 48, 72 h. **(d)** Since IND is a small molecule that may diffuse from trypsinized cells, with the possibility of impacting intracellular drug analysis, we also performed the experiment by washing the pre-chilled monolayer with ice-cold buffer and then extracting in situ before UPLC-MS/MS analysis. There was a minimal change in the intracellular drug ratios compared to the data in **Fig. 3c**. **(e)** The original uncropped western blot scans for **Fig. 3e**. Lanes 1→9 in blot scans represent the control, free IND (0.1, 1, 10, 50 μM) and IND-NV (0.1, 1, 10, 50 μM), sequentially. **p* < 0.05; ***p* < 0.01; #*p* < 0.001 (ANOVA) compared to pH7.4 group or free IND.

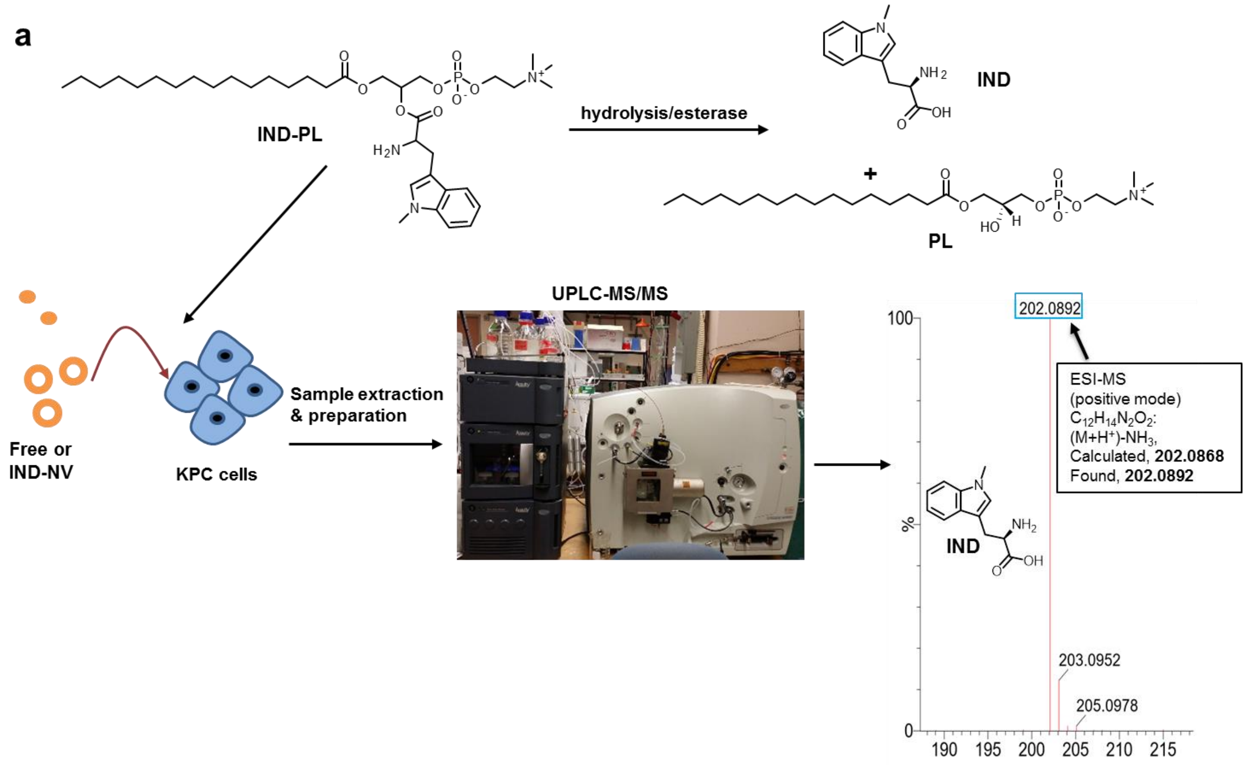

b

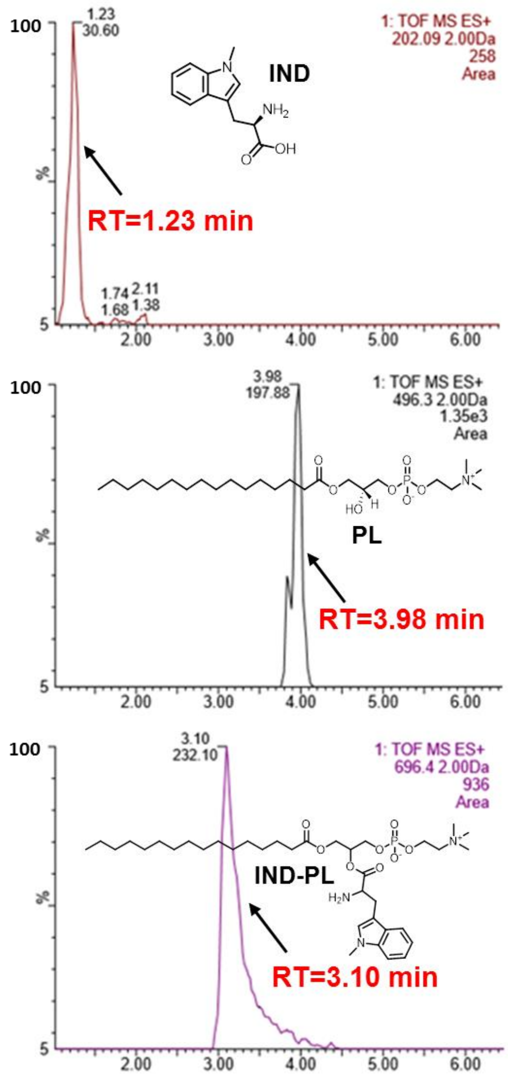

100
1.23
30.60
1: TOF MS ES+
202.09 2.00Da
258
Area
IND
RT=1.23 min
%
1.74 2.11
1.68 1.38
5
2.00
3.00
4.00
5.00
6.00
3.98
197.88
1: TOF MS ES+
496.3 2.00Da
1.35e3
Area
PL
RT=3.98 min
3.10
232.10
1: TOF MS ES+
696.4 2.00Da
936
Area
IND-PL
RT=3.10 min

c

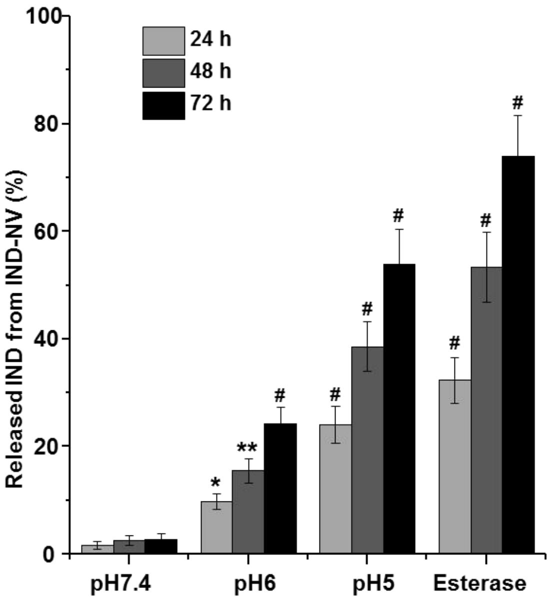

24 h
48 h
72 h
Released IND from IND-NV (%)
100
80
60
40
20
0
pH7.4
pH6
pH5
Esterase
*
**
#

d

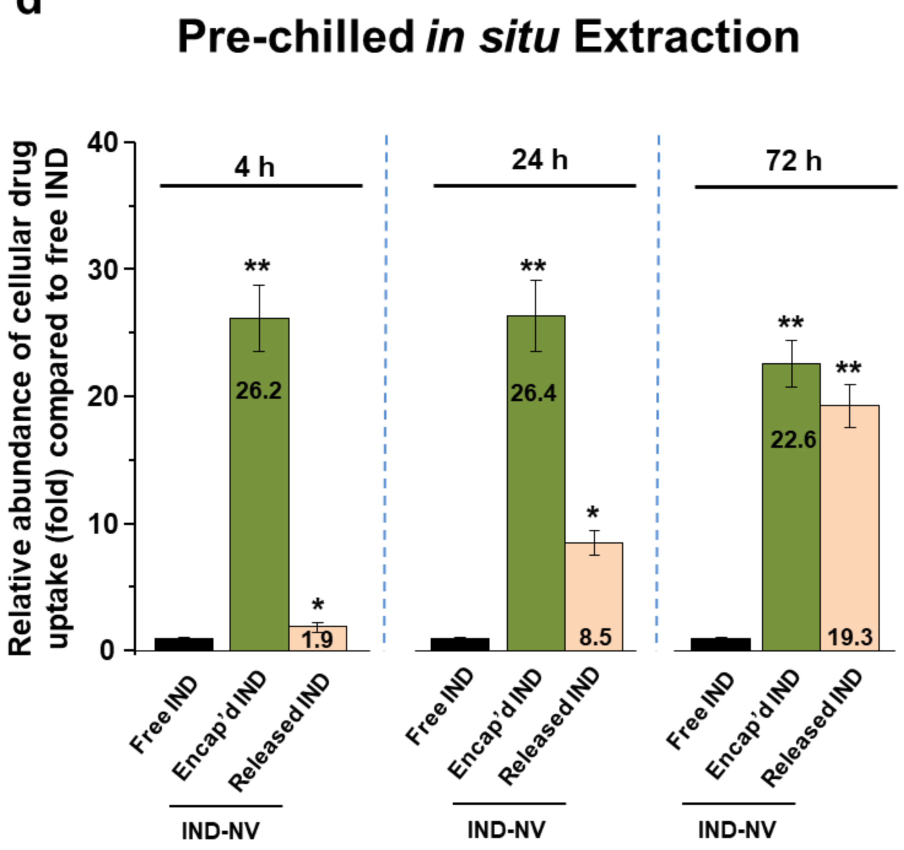

e

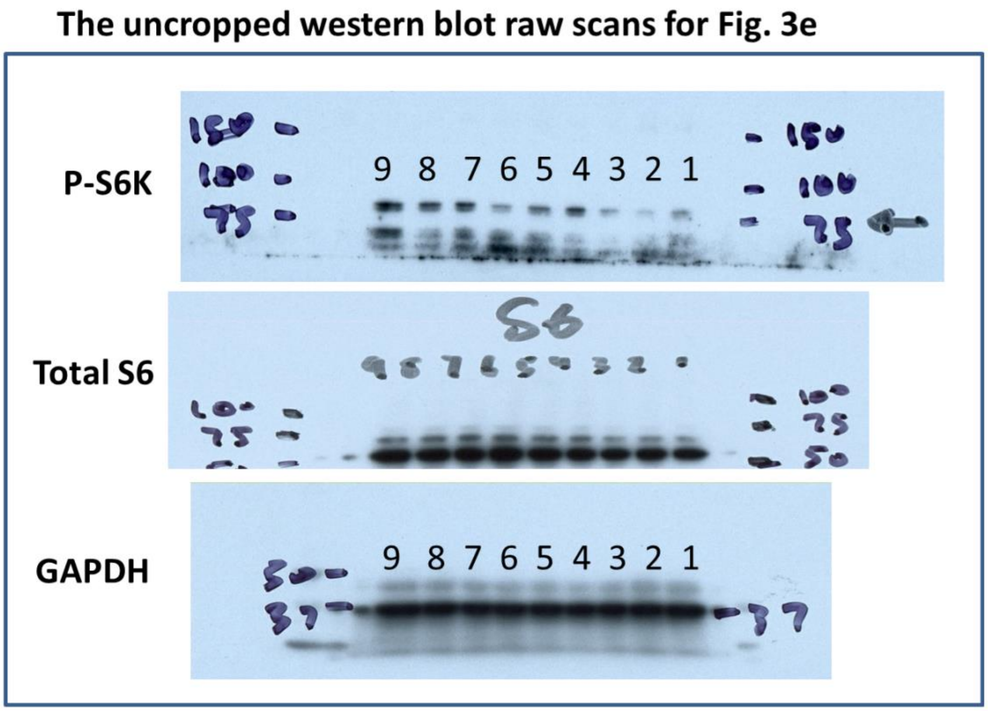

Note: Please be advised that for the ease of view, the picture in Fig. 3e has been flipped, placing the control (#1) on the very left.

Supplementary Figure 6. IHC staining for IDO expression in the orthotopic model, as well as demonstrating the impact of indoximod on IL-6 in the autocrine IL-6/STAT3/aryl hydrocarbon receptor (AHR) signaling loop that is responsible for constitutive IDO expression. IHC staining was performed with an anti-IDO antibody, using tissue sections from the orthotopic tumor experiment shown in **Fig. 6**; control staining was performed with an isotype control antibody **(a).** Scale bar is100 μm. In addition to the impact on the mTOR pathway, the increased IDO expression at the tumor site (as shown in panel a) has also been suggested to induce aryl hydrocarbon receptor (AHR) activation as a result of Kyn accumulation in the TME[11]. Moreover, it has also been suggested that the AHR contributes to constitutive IDO expression at the tumor site by an autocrine signaling loop that involves this receptor, IL-6 and STAT3 as shown in panel (**b**). The enhanced IL-6 expression is thought to derive from the activation of GCN2, a stress response kinase that is stimulated by non-ligated tryptophan-tRNA as a result of tryp depletion by IDO. How exactly IND interferes in this autocrine loop is not clear. In order to determine whether free IND and IND-NV impact IL-6 production in KPC cells, cells were cultured in tryptophan-deficient medium overnight, and then treated with IND or IND-NV at equivalent dose of 0.1 μM, 1 μM, 10 μM, and 50 μM IND for 3 h. The supernatants were collected to assess IL-6 levels by an ELISA kit (BD Biosciences) following manufacturer's instructions (**c**). The pooled results of 3 experiments are shown in panel (**b**), which indicates that IND or IND-NV was able to effectively suppress the release of IL-6 from KPC cells in a dose dependent manner, therefore, leading to the inhibition of the IL-6/STAT3/AHR autocrine signaling loop. *$p < 0.05$ (ANOVA) compared to control.

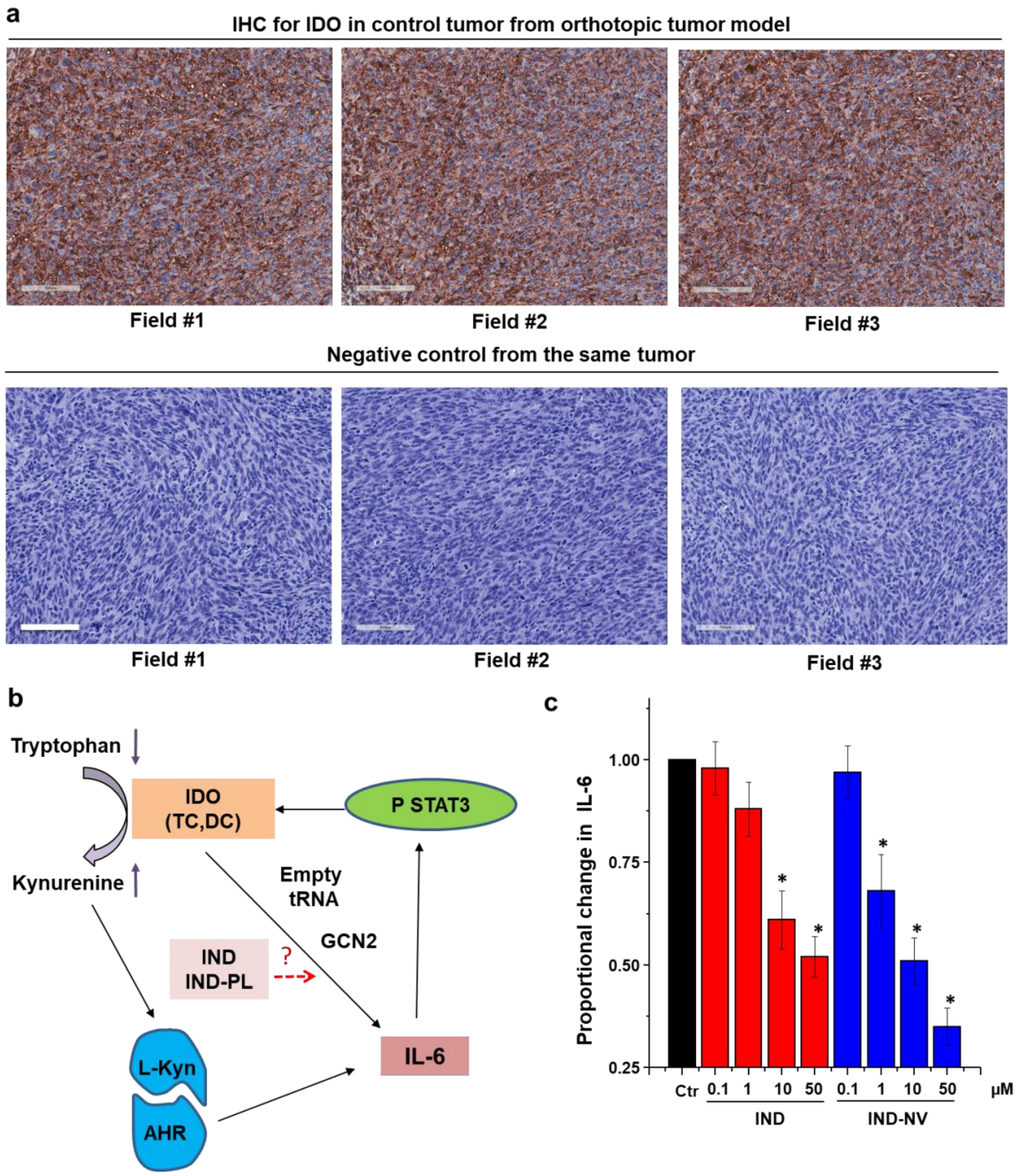

Supplementary Figure 7. The full panel of IHC analysis data in the IT injection experiment (**Fig. 4** in the manuscript) is shown below. This includes the staining for: CD8 (**a**), Foxp3 (**b**), CD4 (**c**), CRT (**d**), CD91 (**e**), HMGB-1 (**f**), TLR4 (**g, upper panel**), IFN-γ (**h**), IL-10 (**i**), and CC-3 (**j**). We also show H&E staining data in panel **(k)**. Moreover, we conducted flow cytometry analysis to quantify TLR4 expression in the population of $CD45^{+}/CD11b^{+}/CD11c^{+}$ cells (**g, lower panel).** **p* < 0.05; ***p* < 0.01, (ANOVA). All the immune biomarkers were visualized by DAB (3,3'-Diaminobenzidine), with exception of CD91 that used Vulcan Fast Red Chromogen Kit 2. Scale bar is 100 μm.

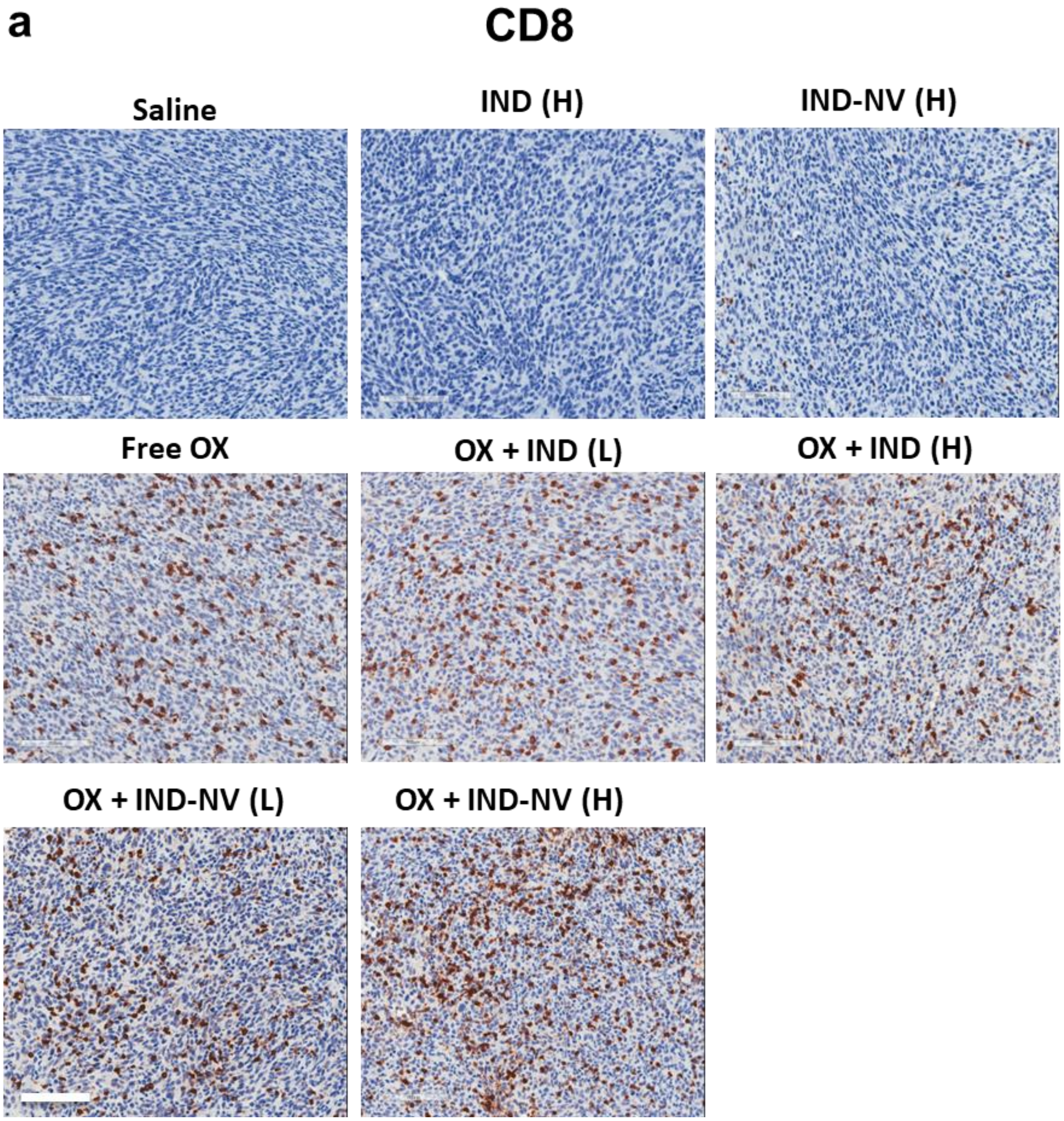

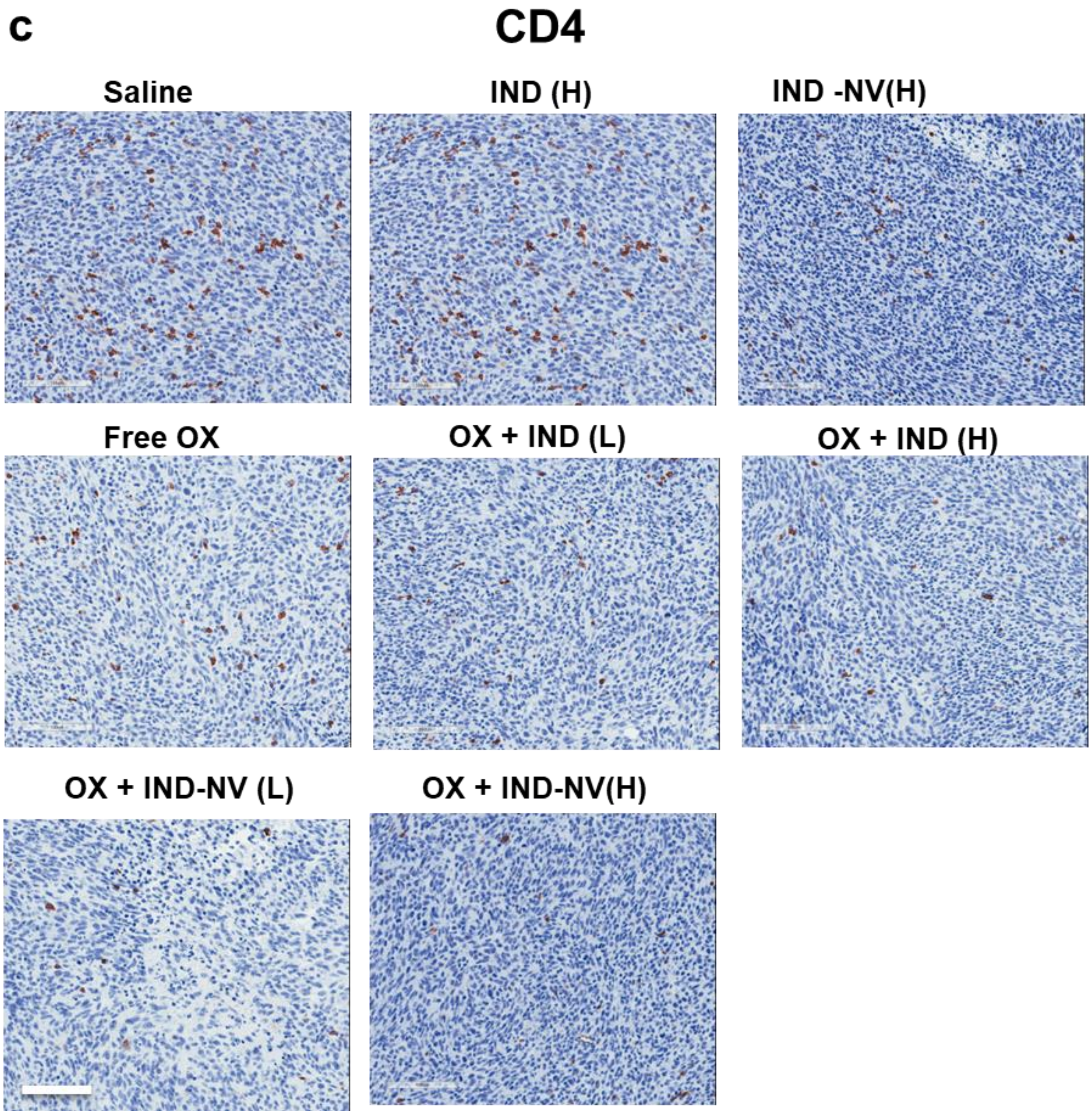
c
CD4
Saline
IND (H)
IND -NV(H)
Free OX
OX + IND (L)
OX + IND (H)
OX + IND-NV (L)
OX + IND-NV(H)

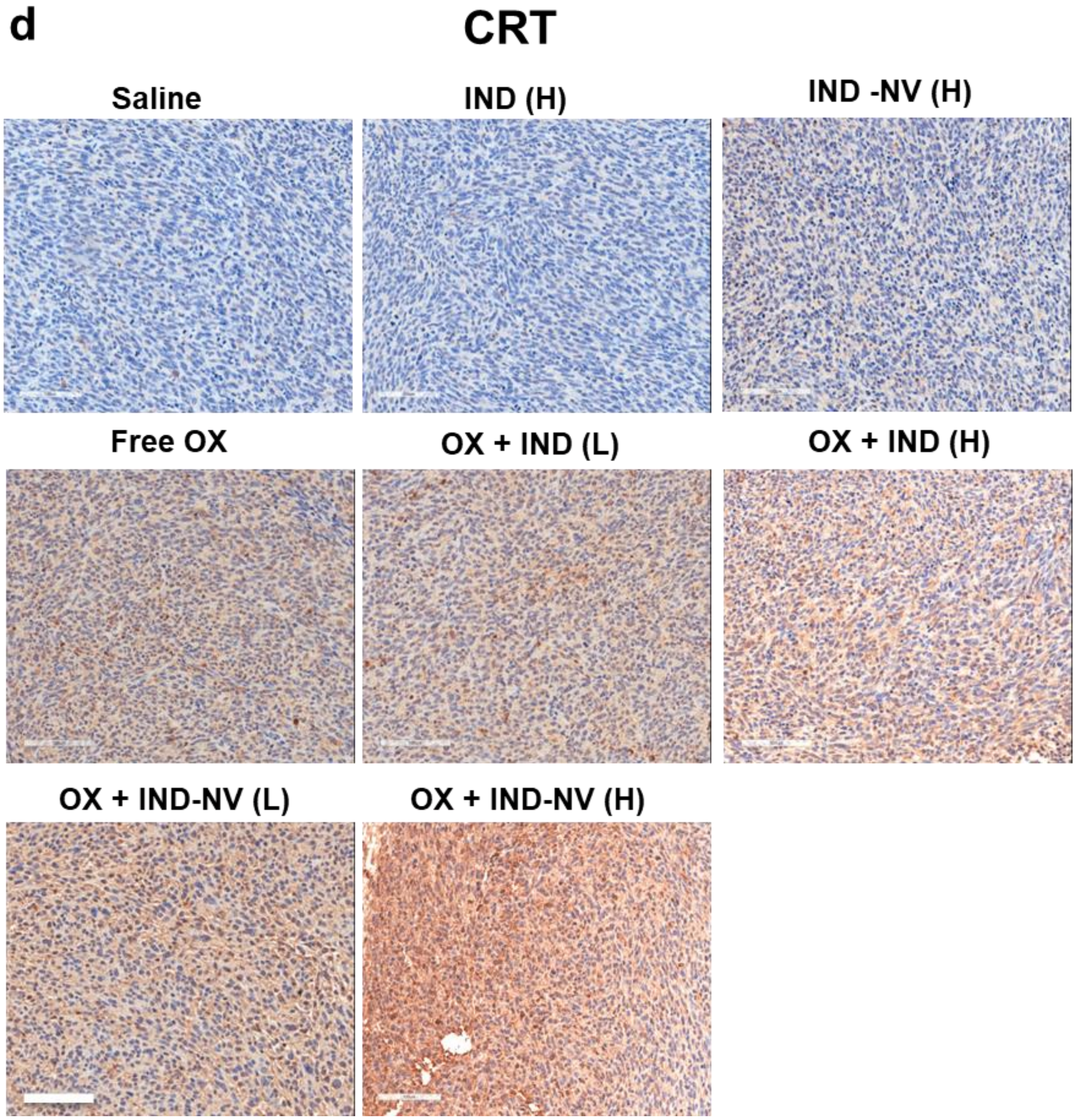
d
CRT
Saline
IND (H)
IND -NV (H)
Free OX
OX + IND (L)
OX + IND (H)
OX + IND-NV (L)
OX + IND-NV (H)

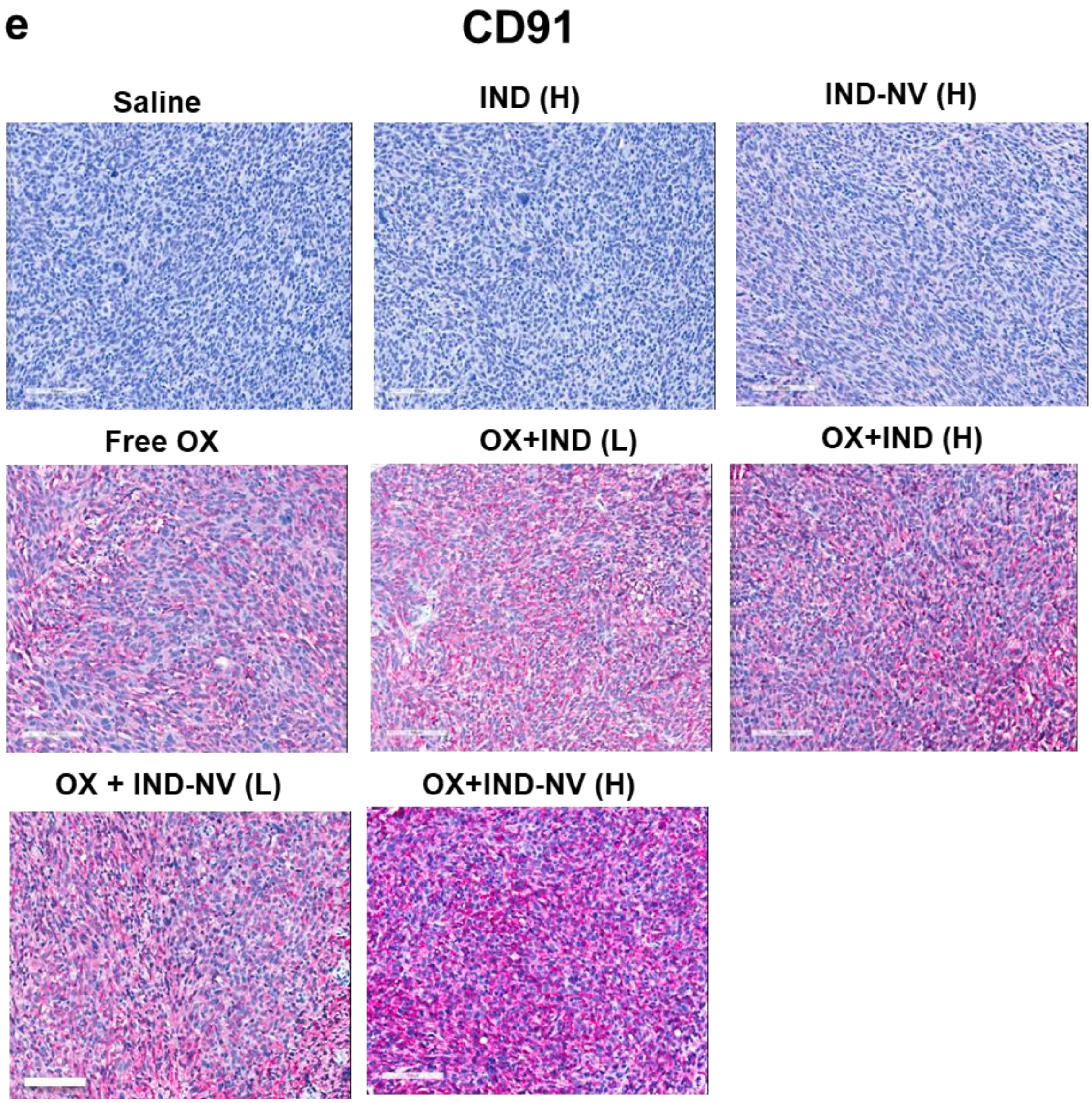
e
CD91
Saline
IND (H)
IND-NV (H)
Free OX
OX+IND (L)
OX+IND (H)
OX + IND-NV (L)
OX+IND-NV (H)

f

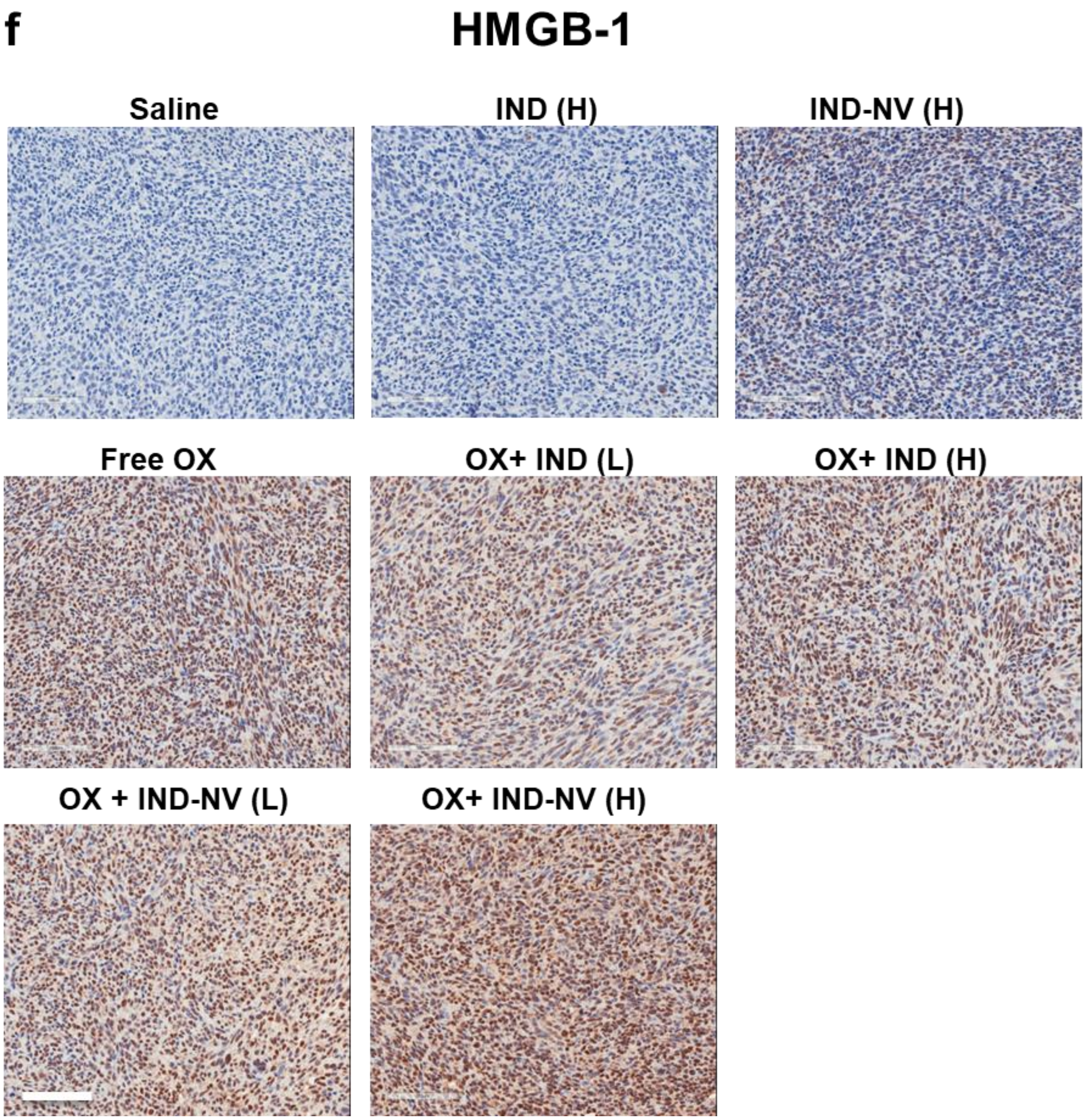
HMGB-1
Saline
IND (H)
IND-NV (H)
Free OX
OX+ IND (L)
OX+ IND (H)
OX + IND-NV (L)
OX+ IND-NV (H)

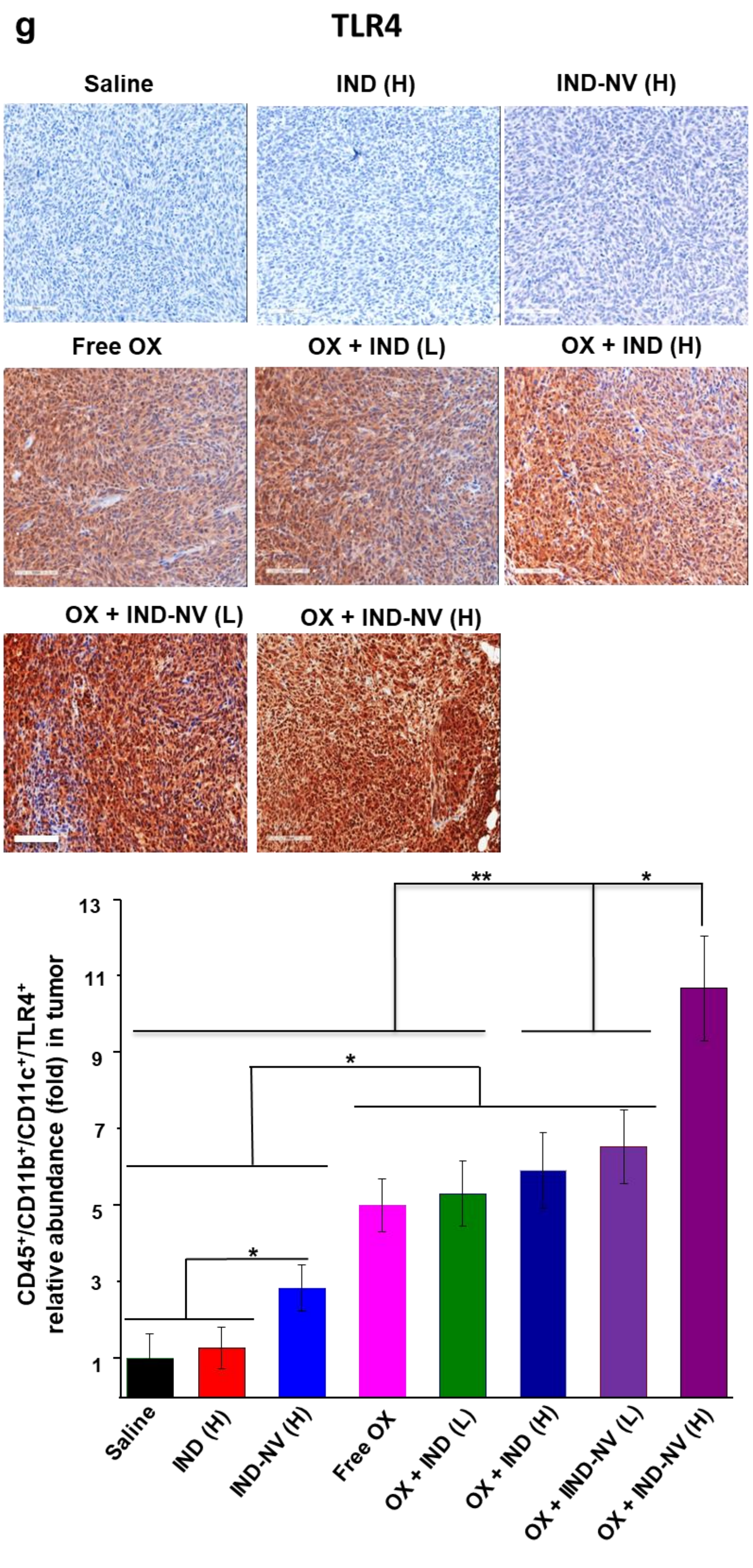

g
TLR4
Saline
IND (H)
IND-NV (H)
Free OX
OX + IND (L)
OX + IND (H)
OX + IND-NV (L)
OX + IND-NV (H)
CD45+/CD11b+/CD11c+/TLR4+ relative abundance (fold) in tumor
13
11
9
7
5
3
1
**
*
*
*
Saline
IND (H)
IND-NV (H)
Free OX
OX + IND (L)
OX + IND (H)
OX + IND-NV (L)
OX + IND-NV (H)

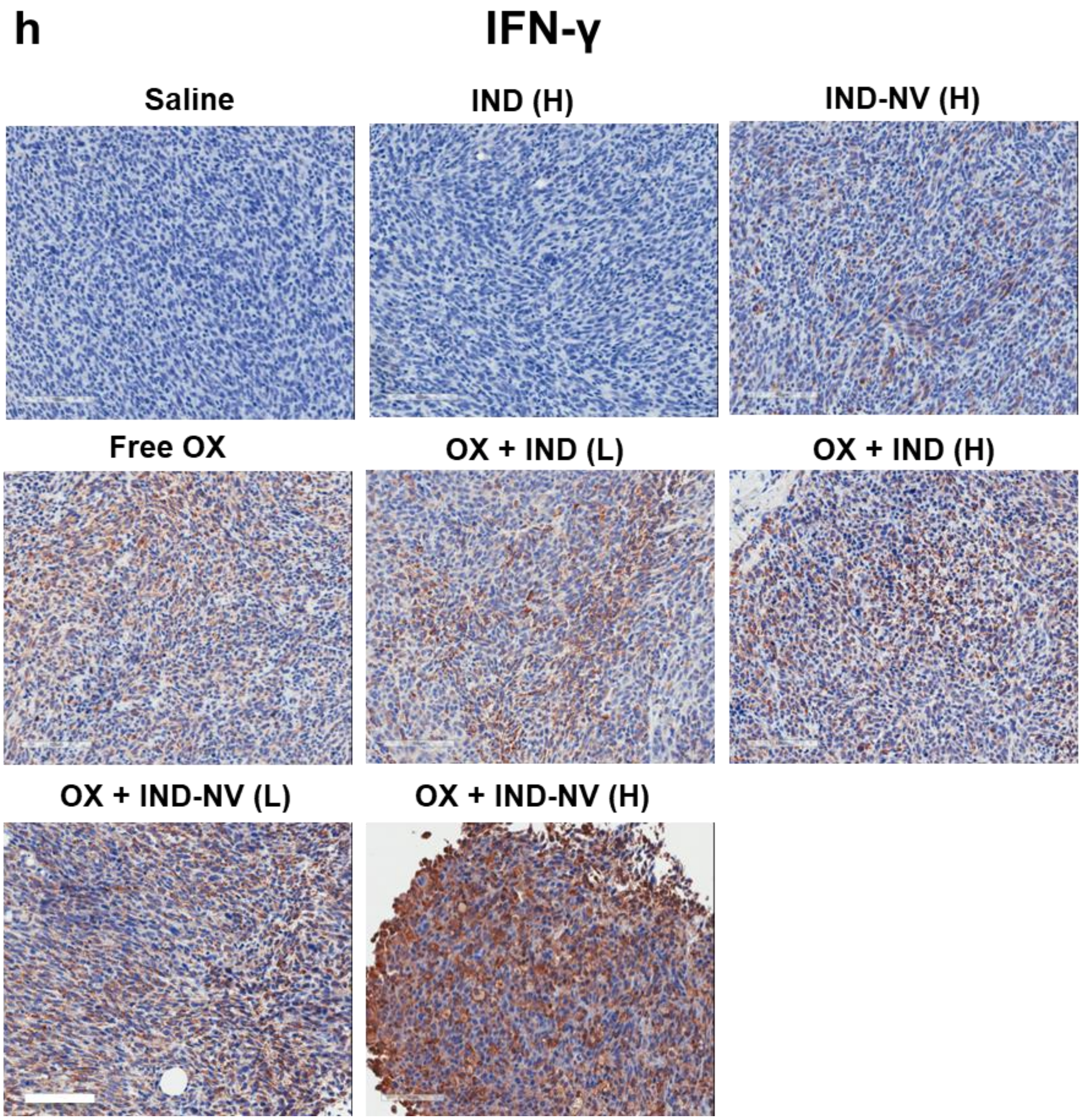
h
IFN-γ
Saline
IND (H)
IND-NV (H)
Free OX
OX + IND (L)
OX + IND (H)
OX + IND-NV (L)
OX + IND-NV (H)

i

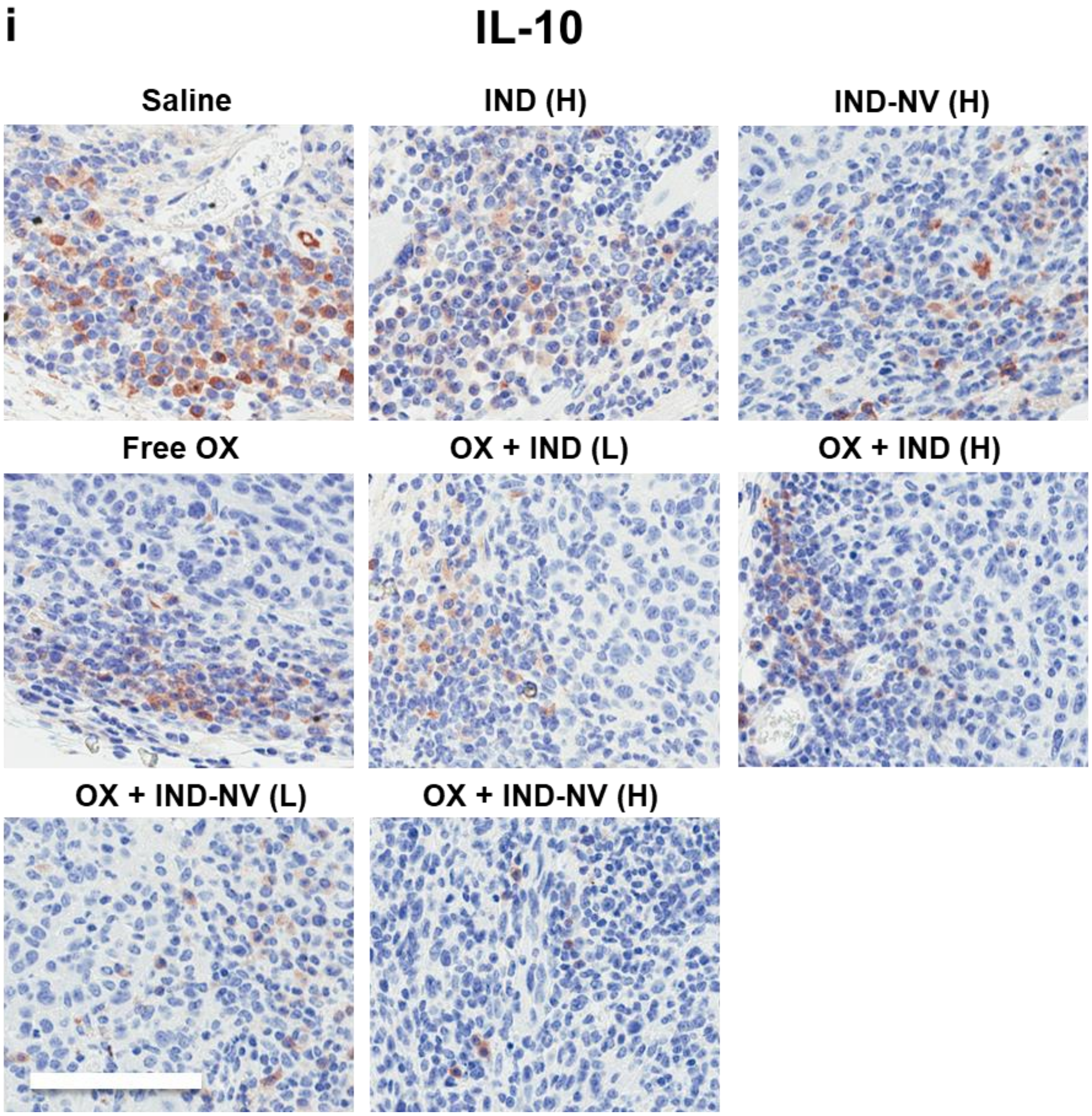
IL-10
Saline
IND (H)
IND-NV (H)
Free OX
OX + IND (L)
OX + IND (H)
OX + IND-NV (L)
OX + IND-NV (H)

j

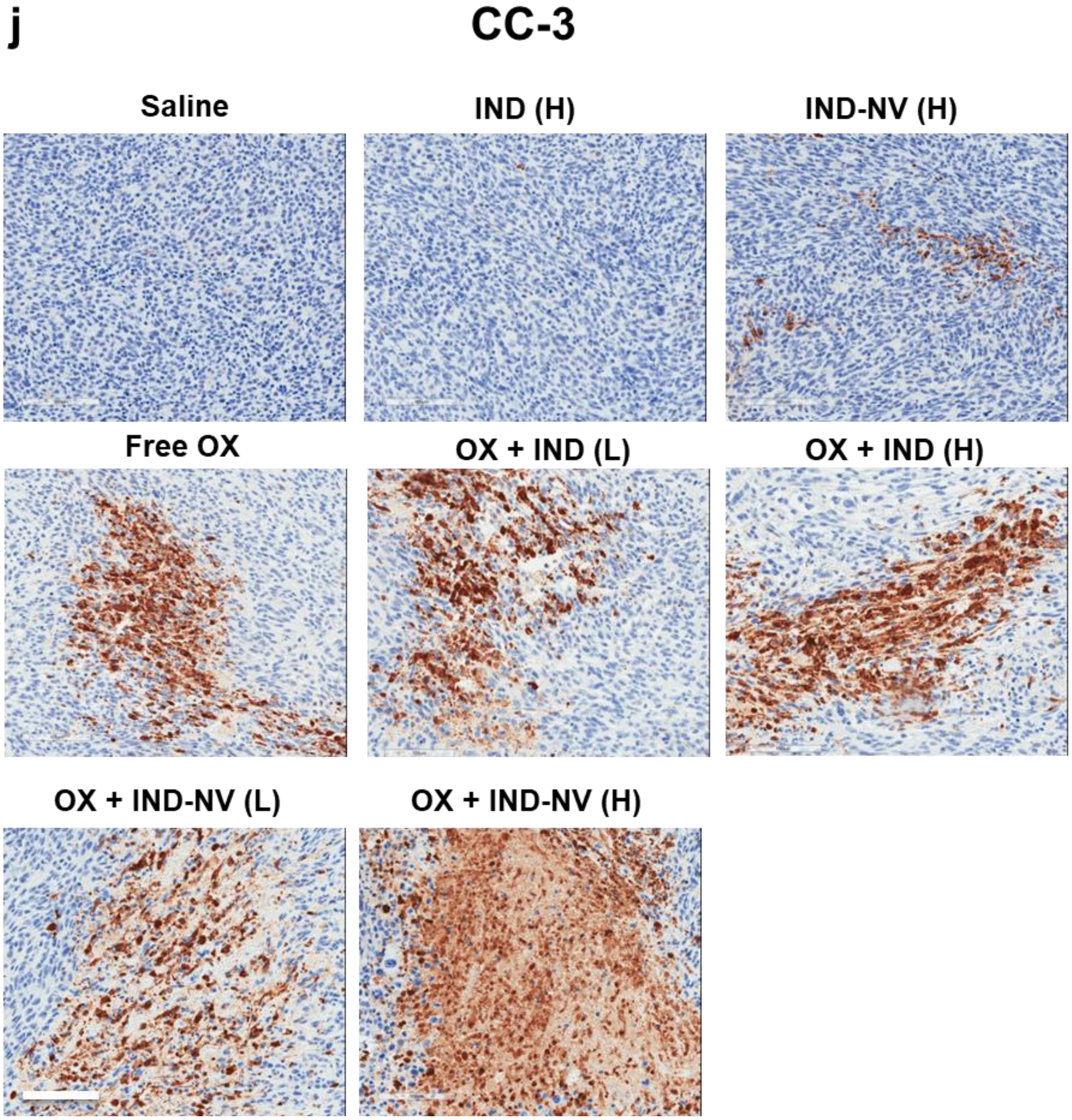
CC-3
Saline
IND (H)
IND-NV (H)
Free OX
OX + IND (L)
OX + IND (H)
OX + IND-NV (L)
OX + IND-NV (H)

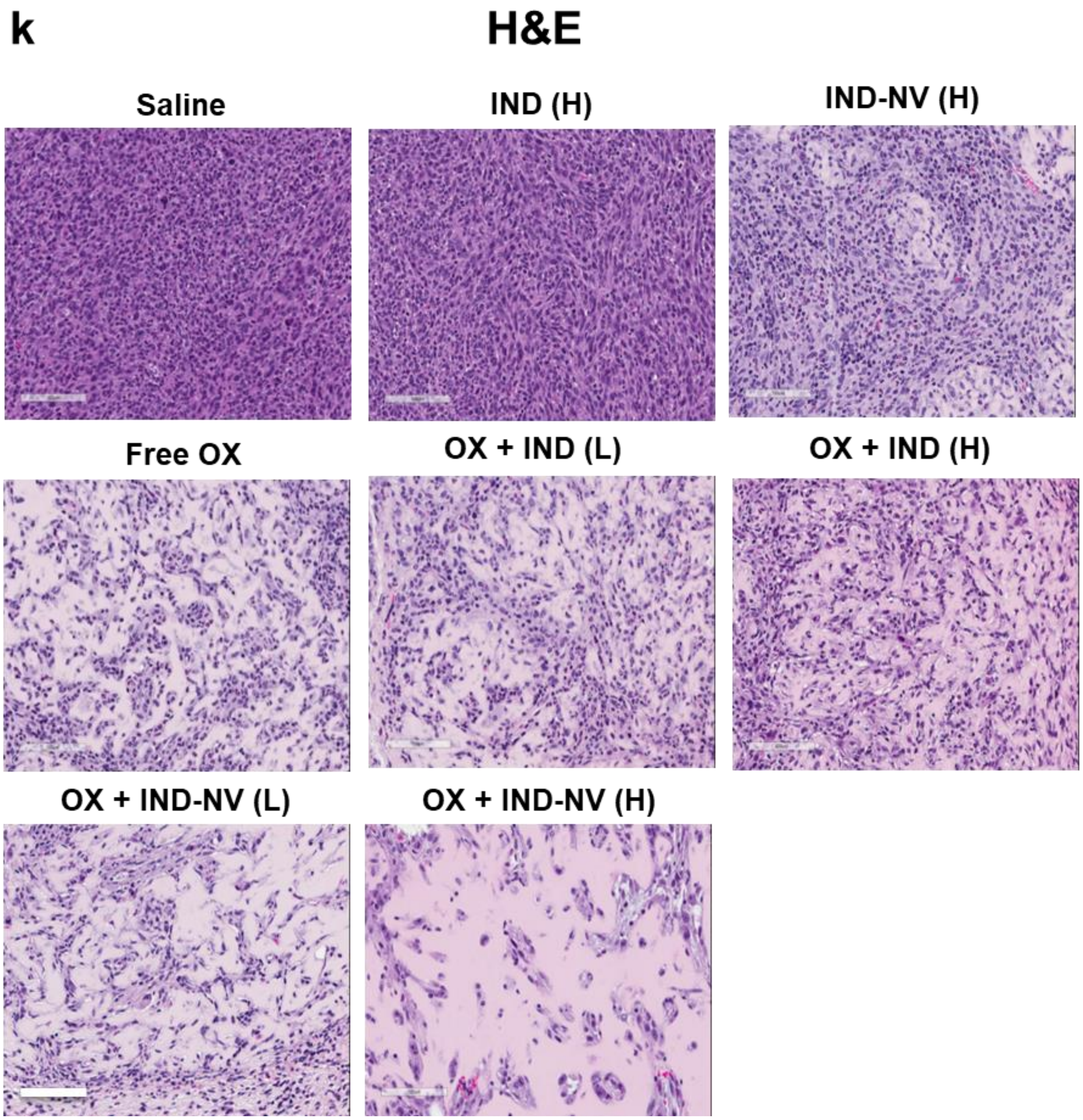
k
H&E
Saline
IND (H)
IND-NV (H)
Free OX
OX + IND (L)
OX + IND (H)
OX + IND-NV (L)
OX + IND-NV (H)

Supplementary Figure 8 (a) Development and optimization of the IND-PL biofilm to coat MSNP with a lipid bilayer. This accomplished by adjusting the ratio of IND-PL vs. cholesterol and DSPE-PEG_{2K} in the lipid mixture. The optimal molar ratio for IND-PL/Cholesterol/DSPE-PEG2K was determined to be 75:25:5 (ratio #4). (**b**) Nanoparticle characterization of OX/IND-MSNP in DI water, PBS, and DMEM containing 10% FBS on days 1 and 30. OX and IND loading capacities were determined to be 4.4% and 44.3%, respectively. The particles had negligible endotoxin content. We show the lower magnification cryoEM pictures for OX/IND-MSNP. **(c)** Low magnification cryoEM of OX/IND-MSNP particles. **(d)** Low magnification cryoEM of OX/LB-MSNP control particle.

a

Development and optimization of IND-MSNP

	**Ratio-1**		**Ratio-2**		**Ratio-3**		**Ratio-4**		**Ratio-5**	
Ind-PHGP	100%		95%		65%		75%		85%	
Cholesterol	0		0		30%		20%		10%	
PEG_{2K}-DSPE	0		5%		5%		5%		5%	
Total lipids	10 mg		10 mg		10 mg		10 mg		10 mg	
MSNP	5 mg		5 mg		5mg		5mg		5mg	
Indoximod loading	62.7%		51.7%		40.0%		44.3%		48.2	
Days	1	30	1	30	1	30	1	30	1	30
Size (nm)	108.4;	129.5	102.5;	116.1	98.5;	119.1	103.5;	107.7	106.7;	122.2
PDI	0.13;	0.14	0.14;	0.13	0.10;	0.15	0.09;	0.06	0.06;	0.13

b

Nanoparticle characterization of OX/IND-MSNP by DLS

**OX/IND-MSNP**	**Size (nm)**	**PDI**	**Zeta (mV)**
DI Water	103.7±2.15	0.09±0.02	-5.29±1.21
After 30 days	109.8±1.84	0.08±0.01	-4.24±0.98
PBS	104.9±1.47	0.08±0.02	-3.54±0.76
After 30 days	111.6±2.62	0.06±0.01	-1.93±0.23
10 % FBS in DMEM	107.5±2.15	0.06±0.01	-4.49±1.14
After 30 days	117.4±2.91	0.11±0.02	-5.63±1.45

Low magnification cryoEM of OX/IND-MSNP

c

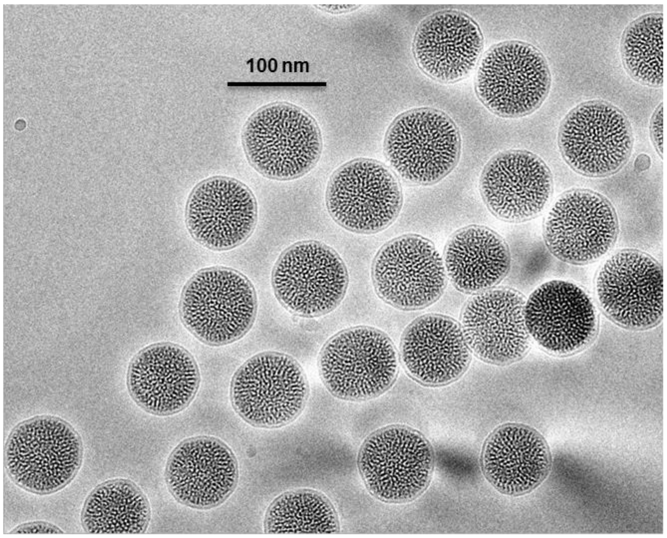

- **Diameter: 83 ±1.6 nm**
- **OX loading: 4.4%**
- **IND loading: 44.3%**
- **Endotoxin level: < 0.1EU/mL**

Low magnification cryoEM of OX/LB-MSNP (control)

d

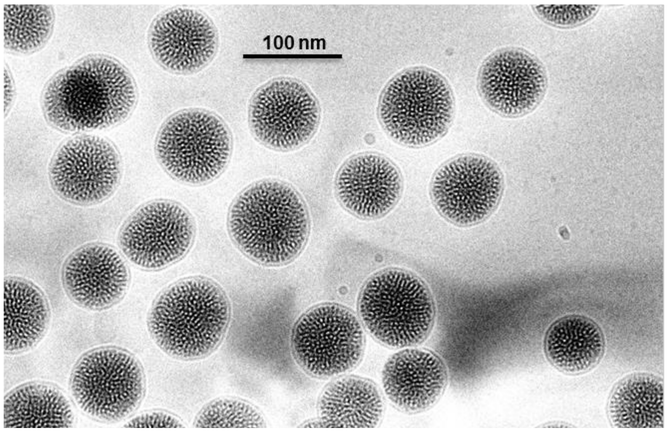

- **Diameter: 82 ±2.1 nm**
- **OX loading: 4.5%**
- **Endotoxin level: < 0.1EU/mL**

Supplementary Figure 9. Comparative PK and tissue distribution of OX by the OX/IND-MSNP dual-delivery carrier vs the OX/LB-MSNP control. ICP-OES analysis to quantify the OX **(a)** and Si **(b)** content in the orthotopic tumor experiment shown in **Fig. 5c**. The animals received IV injection of OX/IND-MSNP (5 mg/kg OX; 50 mg/kg IND, 111 mg/kg Si) or OX/LB-MSNP (5 mg/kg OX; 111 mg/kg Si) (n=6). Blood was collected at indicated time points. 48 h post-IV injection, tumor, heart, liver, spleen, kidney, and lung tissues were harvested and weighed. Tissue pieces were digested in HNO_3/H_2O_2 for OX or Si analysis by ICP-OES, as described previously[2].

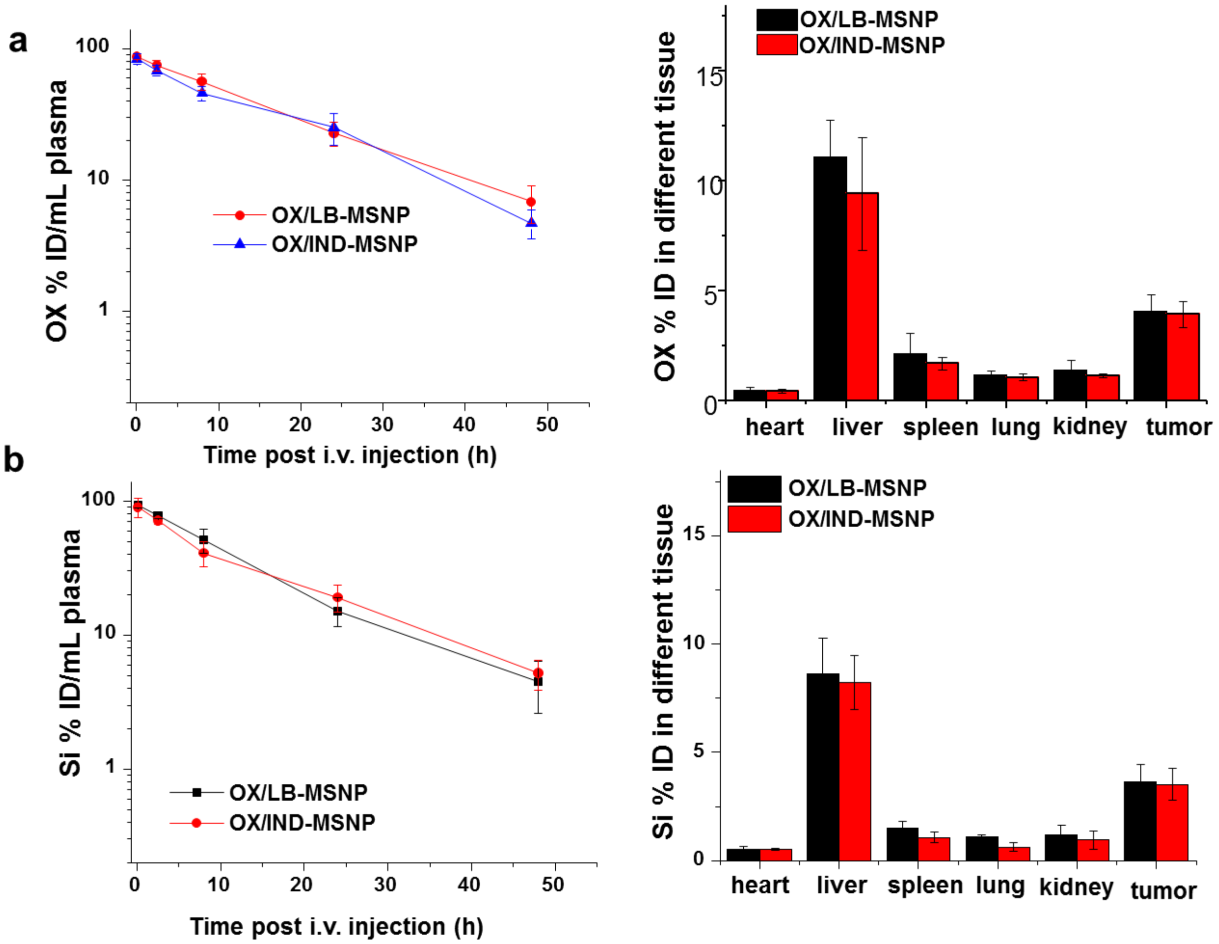

Supplementary Figure 10. (**a**) Representative autopsy images of the animals treated with the dual delivery MSNP, as described in **Fig. 6c**. The broken lines highlight the primary tumors and their local invasion, while the arrows point to metastases. (**b**) Heat map summary of the *ex vivo* imaging data after tumor and organ harvesting for the experiment shown in **Fig. 6c**. This demonstrates a significant reduction in tumor metastases in response to OX/IND-MSNP treatment, and to a lesser extent OX/LB-MSNP.

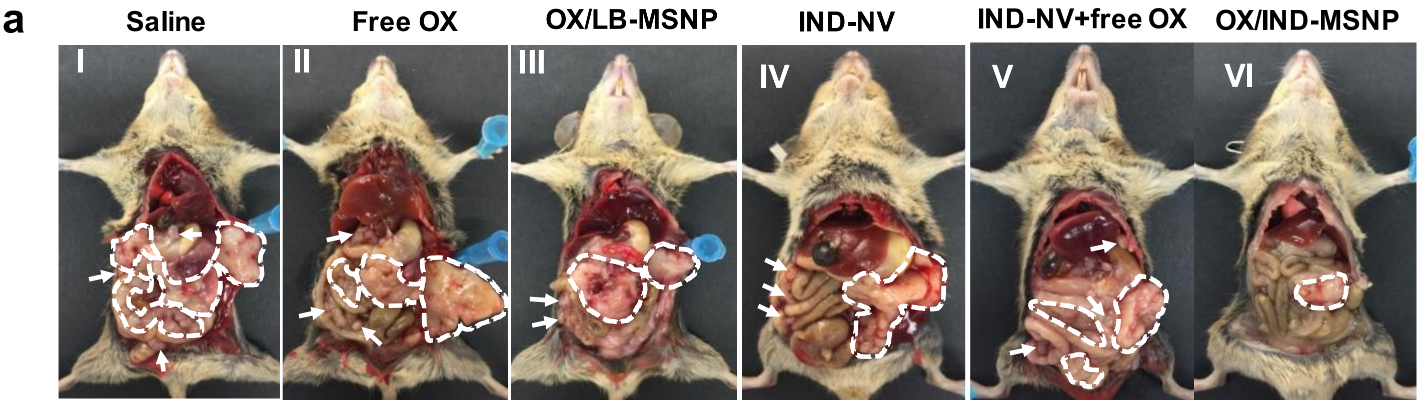

b

Spleen	100	100	71.4	100	100	28.6
Liver	100	85.7	57.1	100	85.7	14.3
Intestines	100	100	71.4	85.7	85.7	28.6
Kidneys	85.7	71.4	57.1	85.7	85.7	28.6
Abdomen wall	100	100	71.4	100	100	42.9
Stomach	100	100	57.1	100	85.7	28.6
Diaphragm	100	100	42.9	100	100	28.6
Blood ascites	100	100	57.1	100	100	28.6

Metastasis frequency	0	10	20	30	40	50	60	70	80	90	100

Supplementary Figure 11. The full panel of IHC data collected during the systemic delivery experiment in **Fig. 6,** showing results for the following markers: CD8 (**a**), Foxp3 (**b**), CRT (**c**), CD91 (**d, upper panel**), HMGB-1 (**e**), TLR4 (**f, upper panel**), IL-12p70 **(g)**, IFN-γ (**h**), perforin (**i**), IL-10 (**j**), and CC-3 (**k**). We also included flow cytometry analysis of CD91 positive cells in the $CD45^+/CD11b^+/CD11c^+$ cell population in the lower panel in **d,** and the same analysis for TLR4 and CD103 positive cells in similarly phenotyped cells in panel **f** and **l,** respectively. **p* < 0.05; ***p* < 0.01, (ANOVA). Scale bar is100 μm.

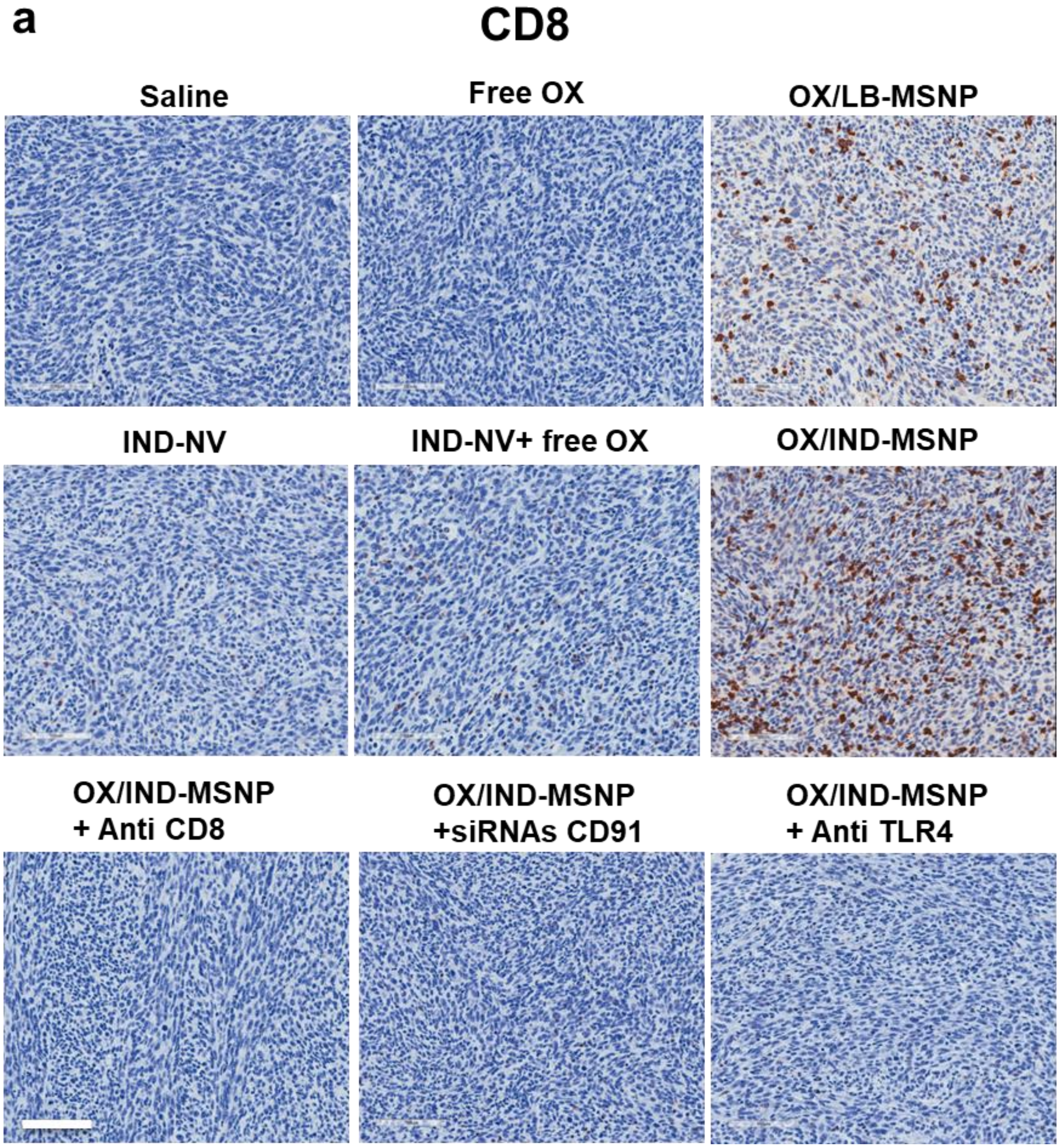

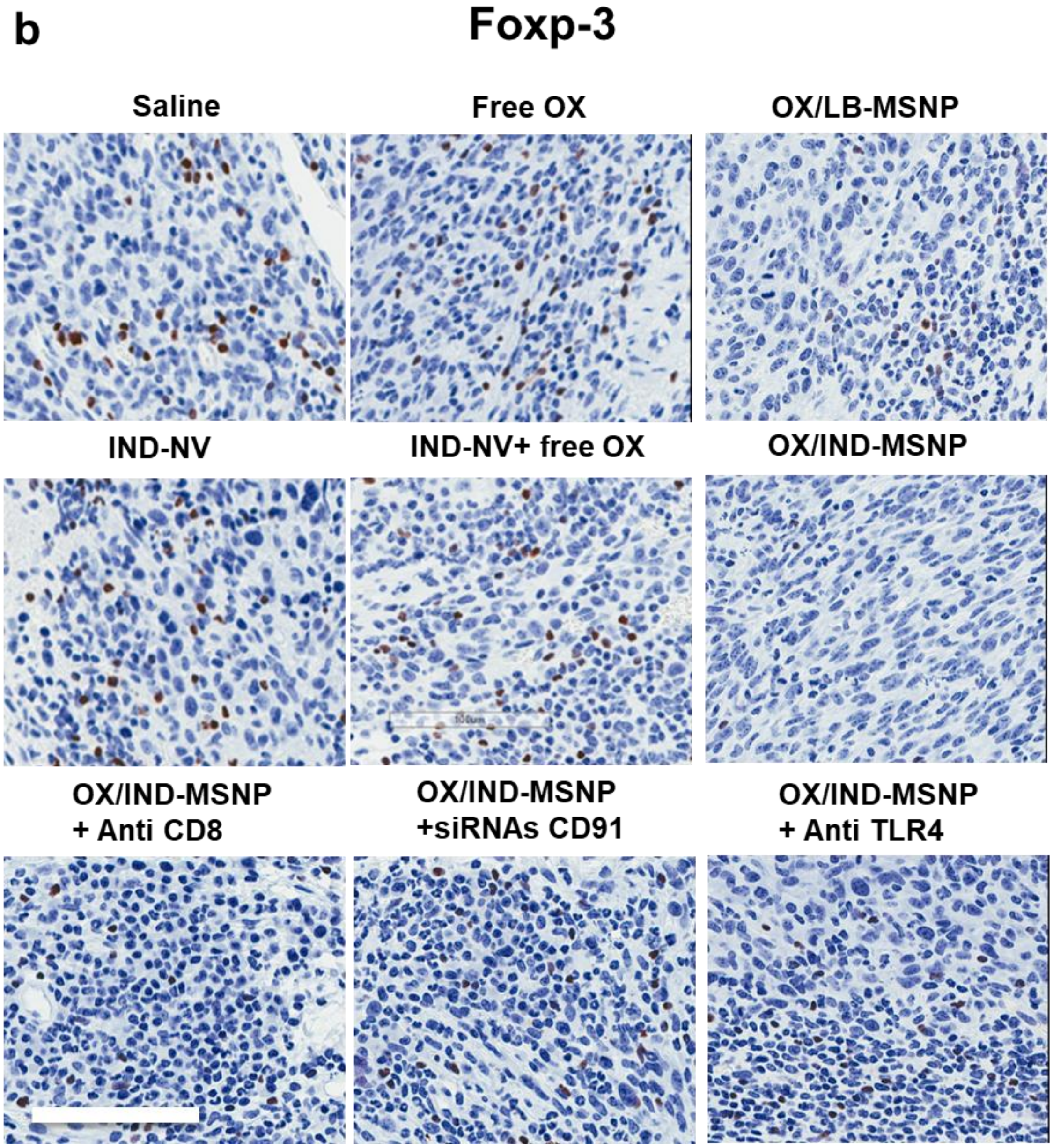
b
Foxp-3
Saline
Free OX
OX/LB-MSNP
IND-NV
IND-NV+ free OX
OX/IND-MSNP
OX/IND-MSNP + Anti CD8
OX/IND-MSNP +siRNAs CD91
OX/IND-MSNP + Anti TLR4

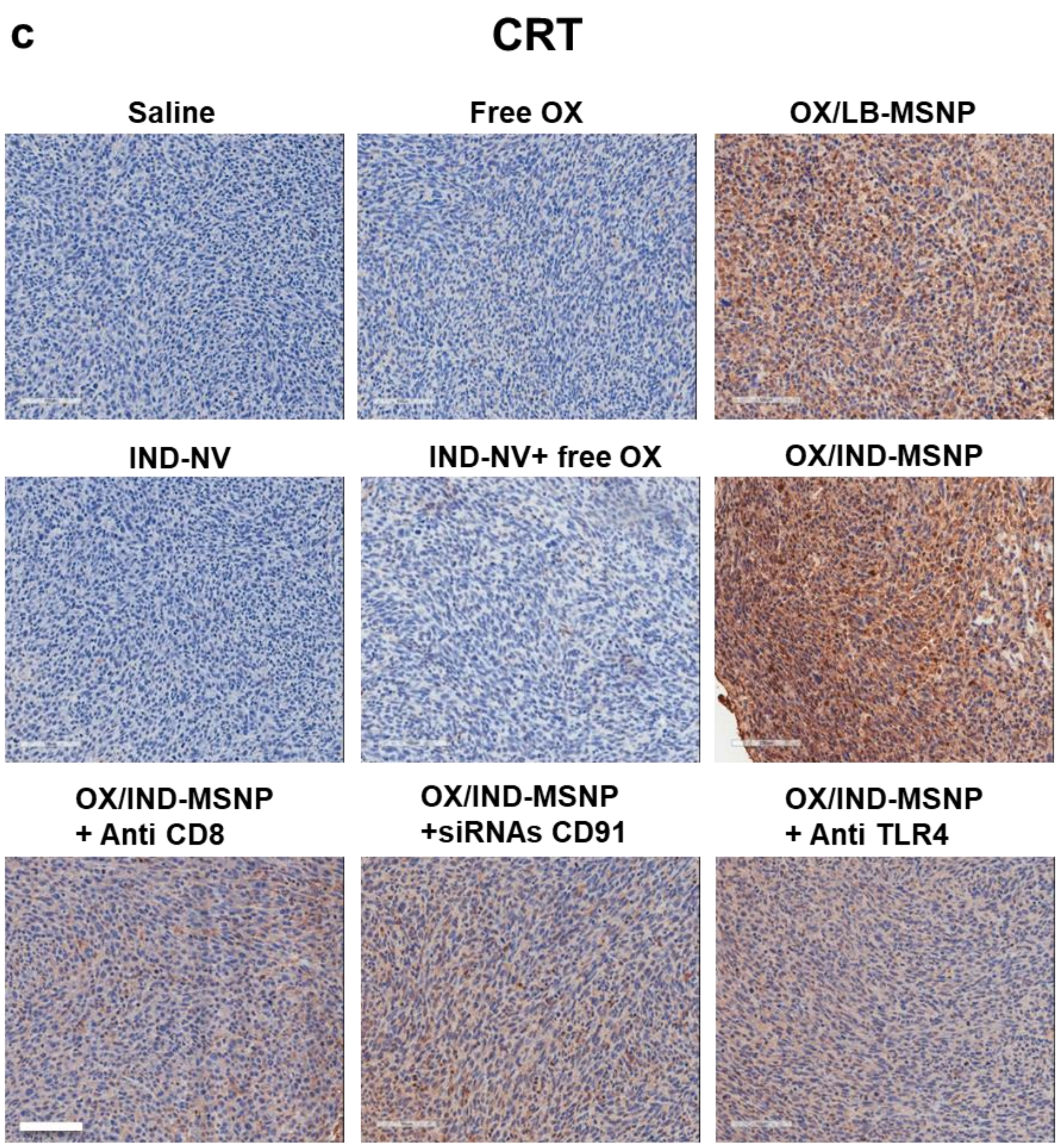
c
CRT
Saline
Free OX
OX/LB-MSNP
IND-NV
IND-NV+ free OX
OX/IND-MSNP
OX/IND-MSNP
+ Anti CD8
OX/IND-MSNP
+siRNAs CD91
OX/IND-MSNP
+ Anti TLR4

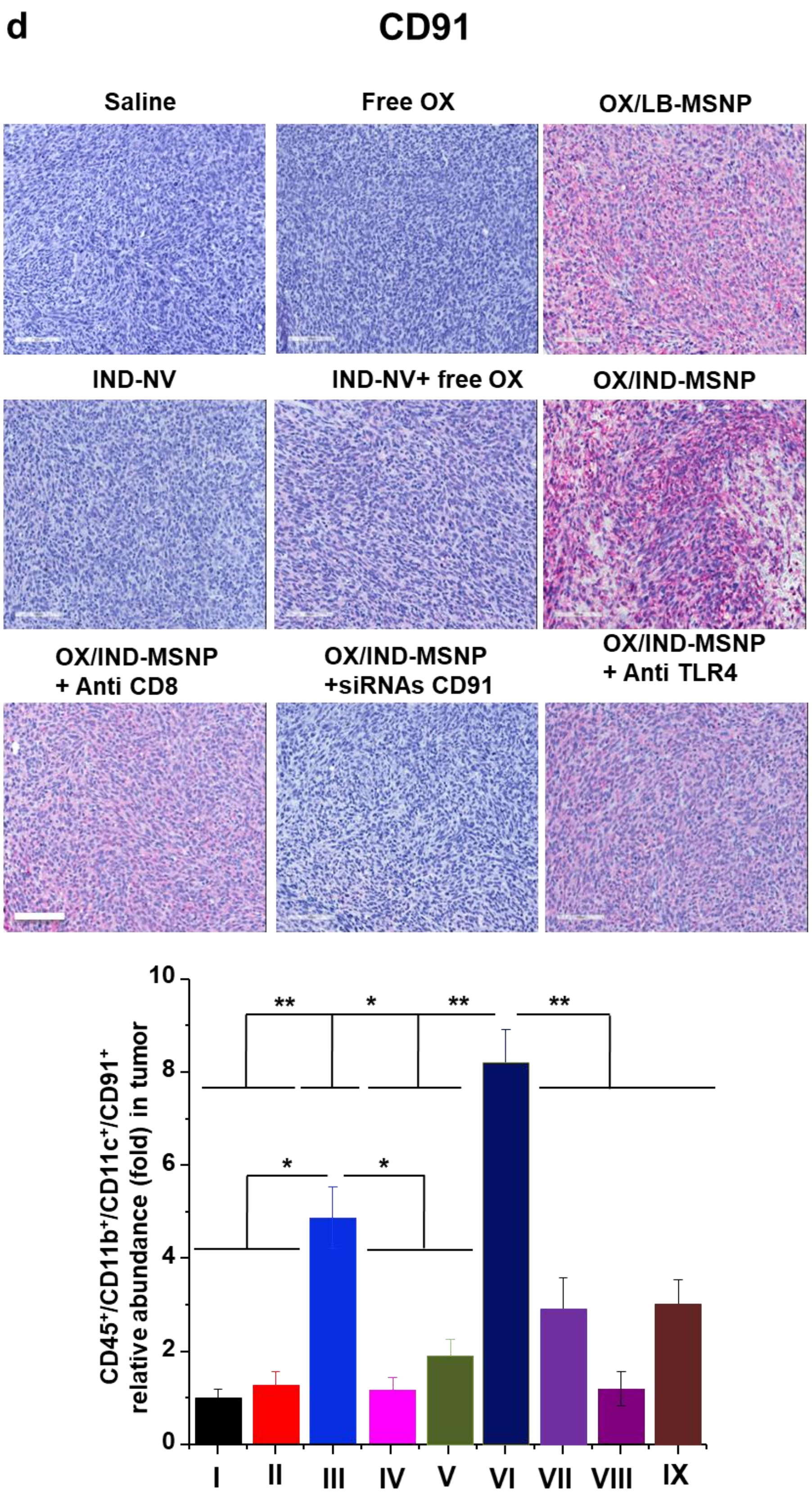
d
CD91
Saline
Free OX
OX/LB-MSNP
IND-NV
IND-NV+ free OX
OX/IND-MSNP
OX/IND-MSNP + Anti CD8
OX/IND-MSNP +siRNAs CD91
OX/IND-MSNP + Anti TLR4
CD45+/CD11b+/CD11c+/CD91+ relative abundance (fold) in tumor
0
2
4
6
8
10
**
*
**
**
*
*
I
II
III
IV
V
VI
VII
VIII
IX

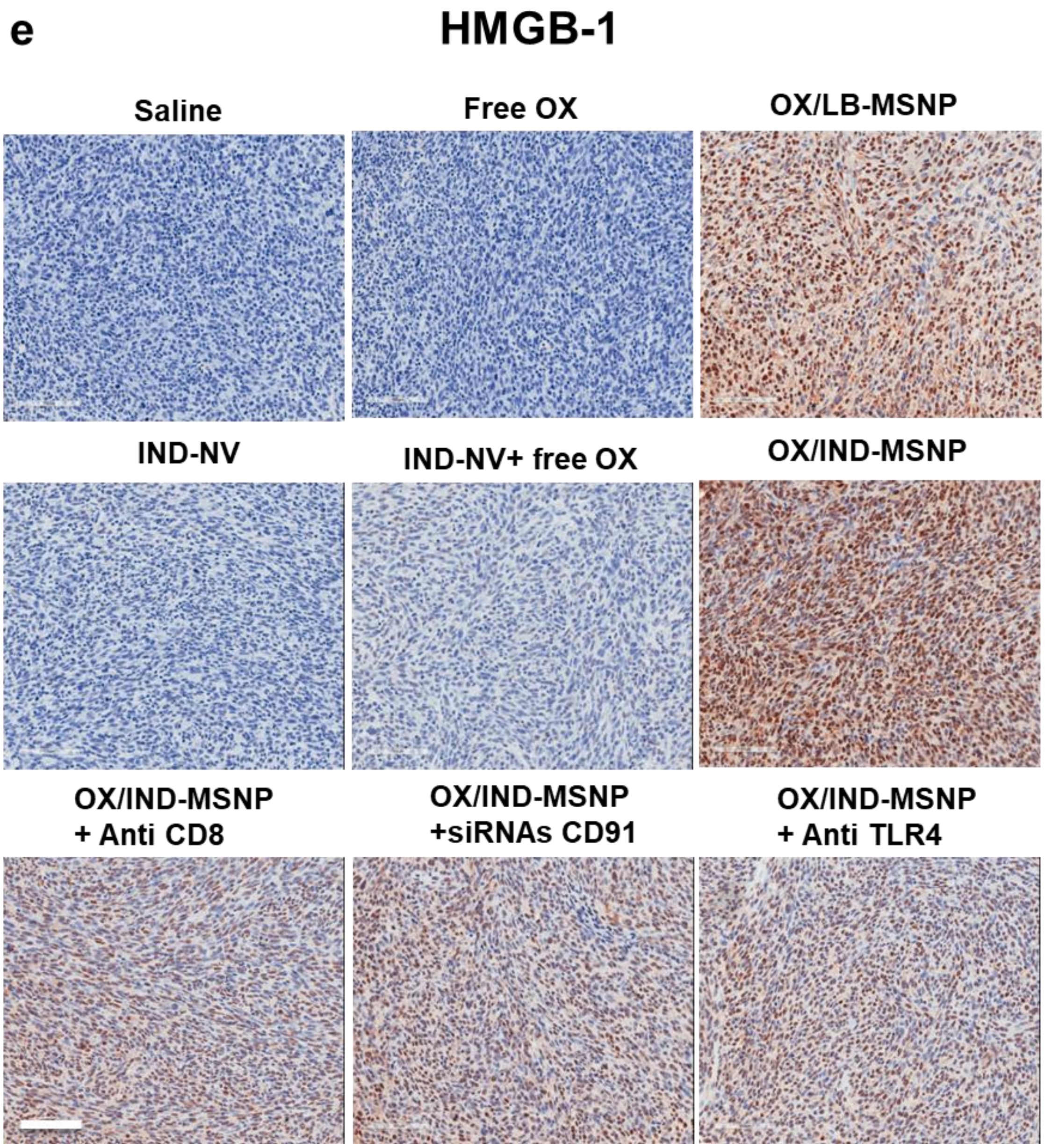
e
HMGB-1
Saline
Free OX
OX/LB-MSNP
IND-NV
IND-NV+ free OX
OX/IND-MSNP
OX/IND-MSNP
+ Anti CD8
OX/IND-MSNP
+siRNAs CD91
OX/IND-MSNP
+ Anti TLR4

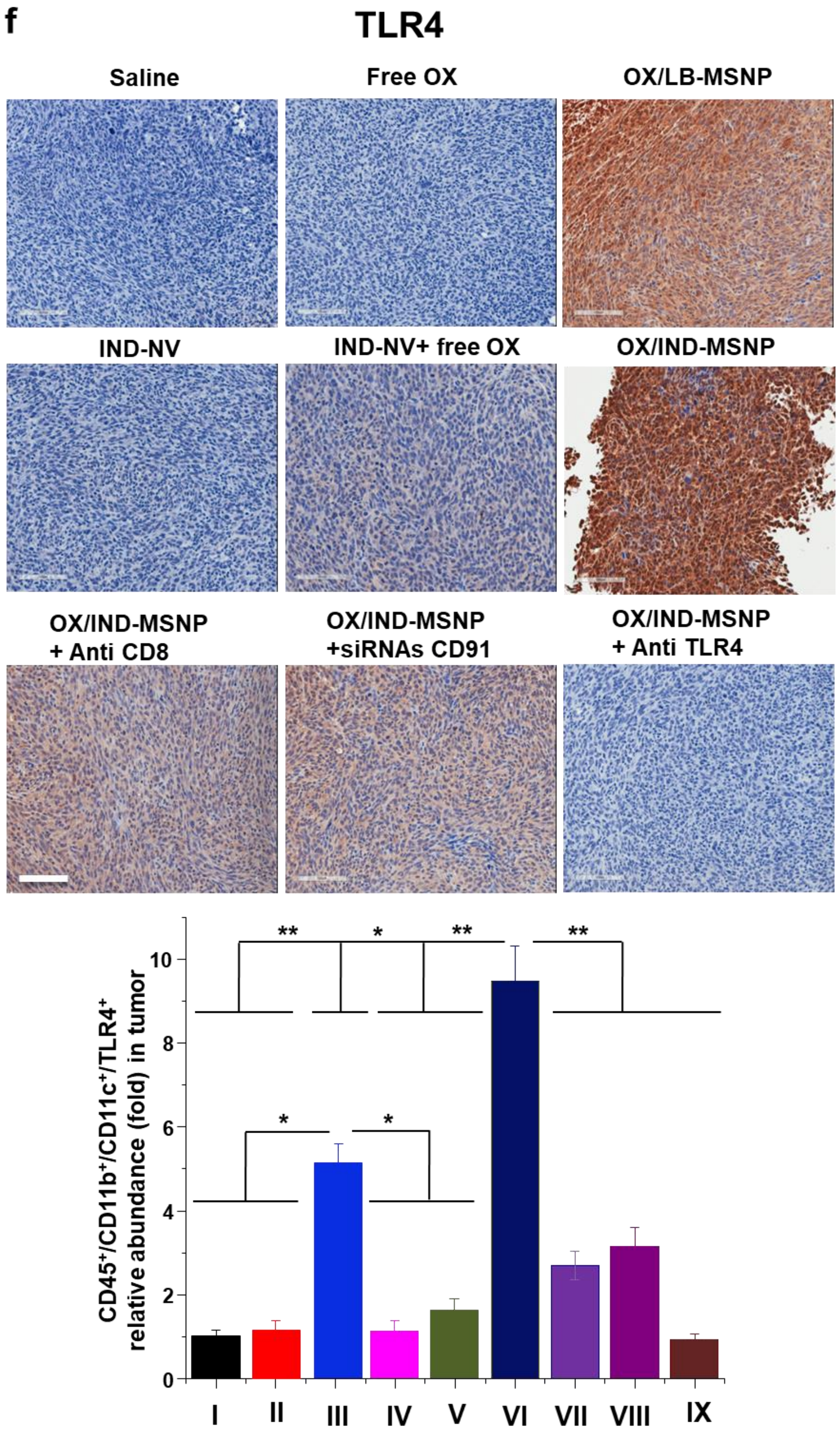
f
TLR4
Saline
Free OX
OX/LB-MSNP
IND-NV
IND-NV+ free OX
OX/IND-MSNP
OX/IND-MSNP
+ Anti CD8
OX/IND-MSNP
+siRNAs CD91
OX/IND-MSNP
+ Anti TLR4
CD45+/CD11b+/CD11c+/TLR4+
relative abundance (fold) in tumor
0
2
4
6
8
10
**
*
**
**
*
*
I
II
III
IV
V
VI
VII
VIII
IX

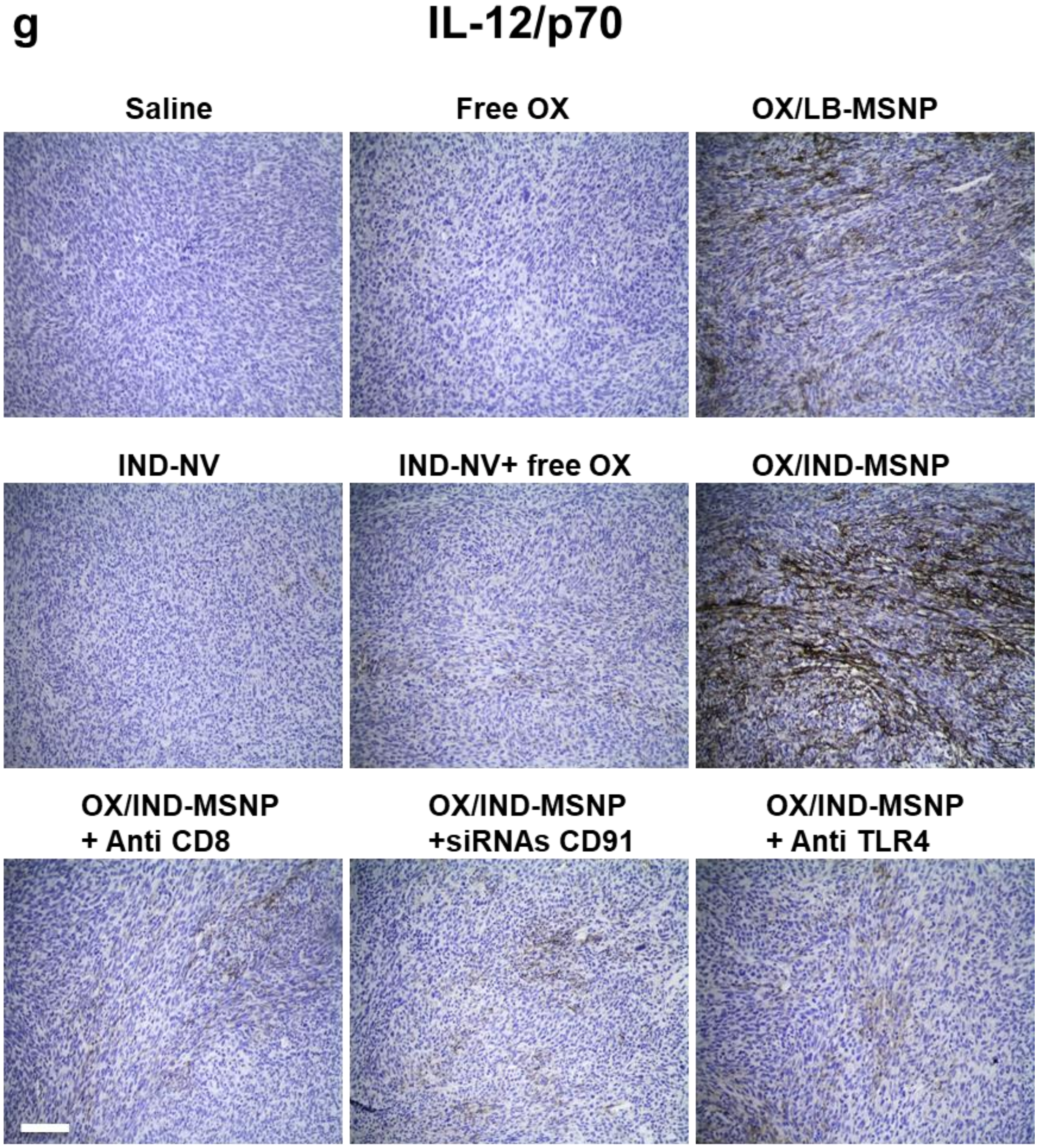
g
IL-12/p70
Saline
Free OX
OX/LB-MSNP
IND-NV
IND-NV+ free OX
OX/IND-MSNP
OX/IND-MSNP
+ Anti CD8
OX/IND-MSNP
+siRNAs CD91
OX/IND-MSNP
+ Anti TLR4

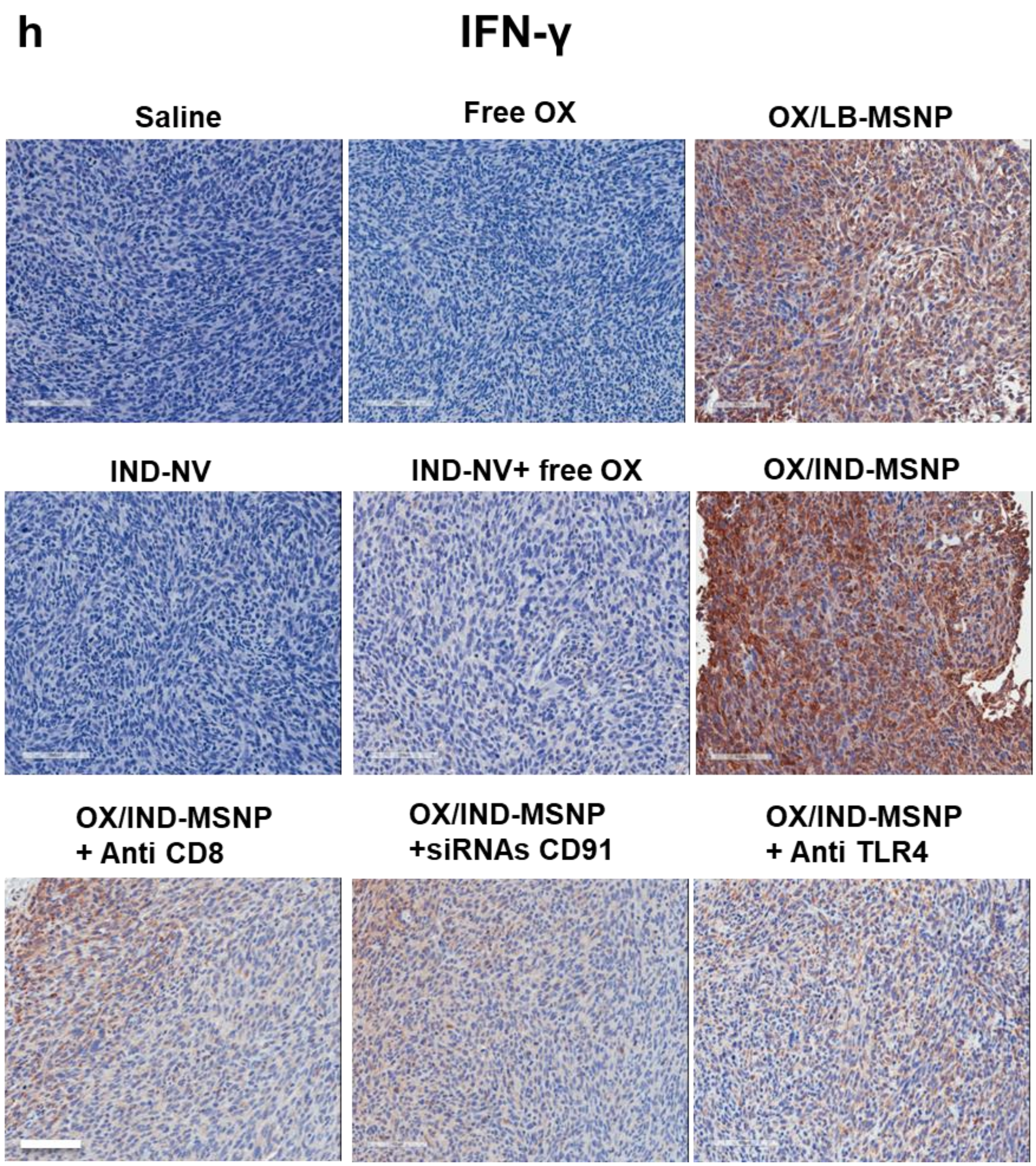
h
IFN-γ
Saline
Free OX
OX/LB-MSNP
IND-NV
IND-NV+ free OX
OX/IND-MSNP
OX/IND-MSNP
+ Anti CD8
OX/IND-MSNP
+siRNAs CD91
OX/IND-MSNP
+ Anti TLR4

i

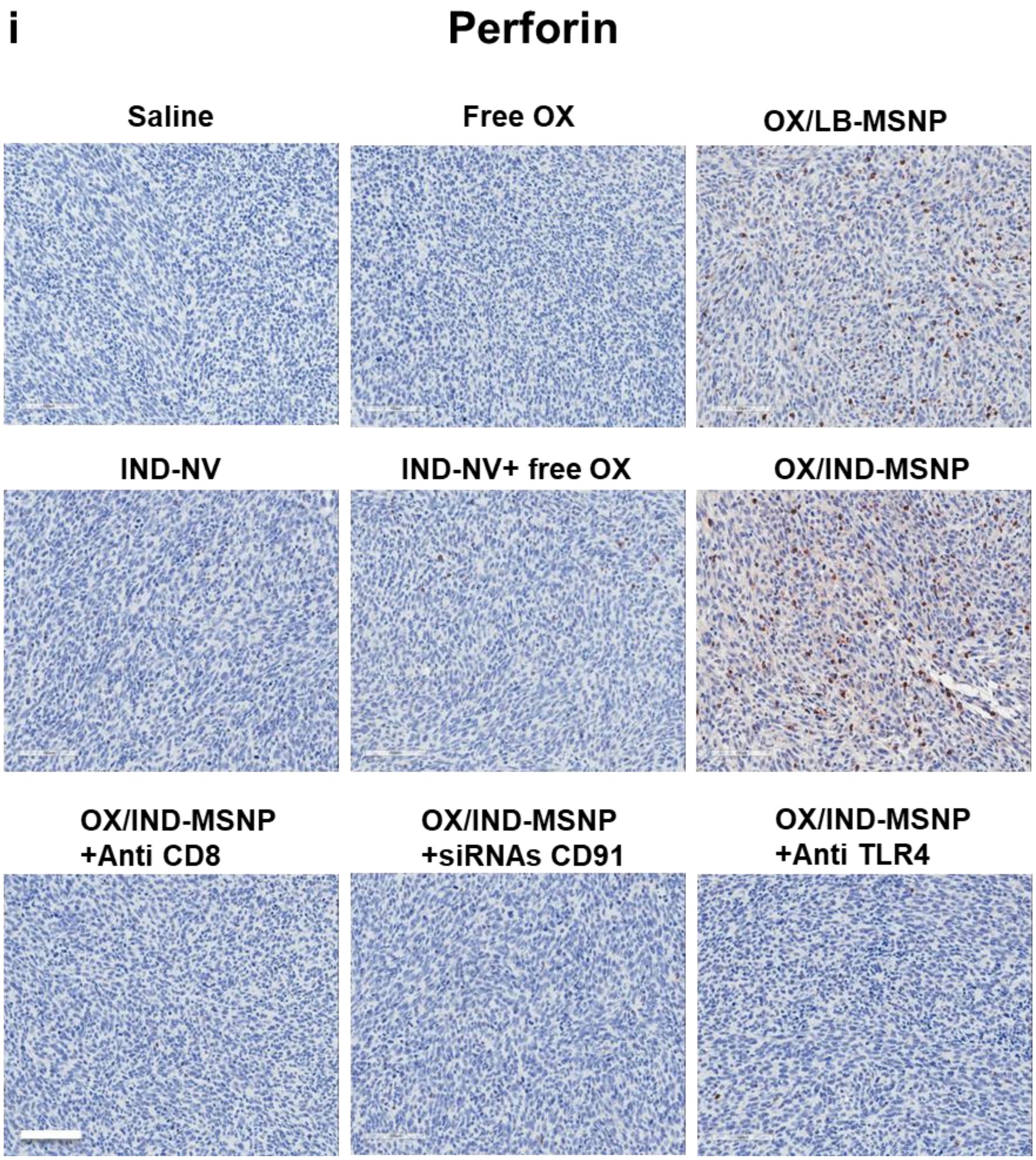
Perforin
Saline
Free OX
OX/LB-MSNP
IND-NV
IND-NV+ free OX
OX/IND-MSNP
OX/IND-MSNP +Anti CD8
OX/IND-MSNP +siRNAs CD91
OX/IND-MSNP +Anti TLR4

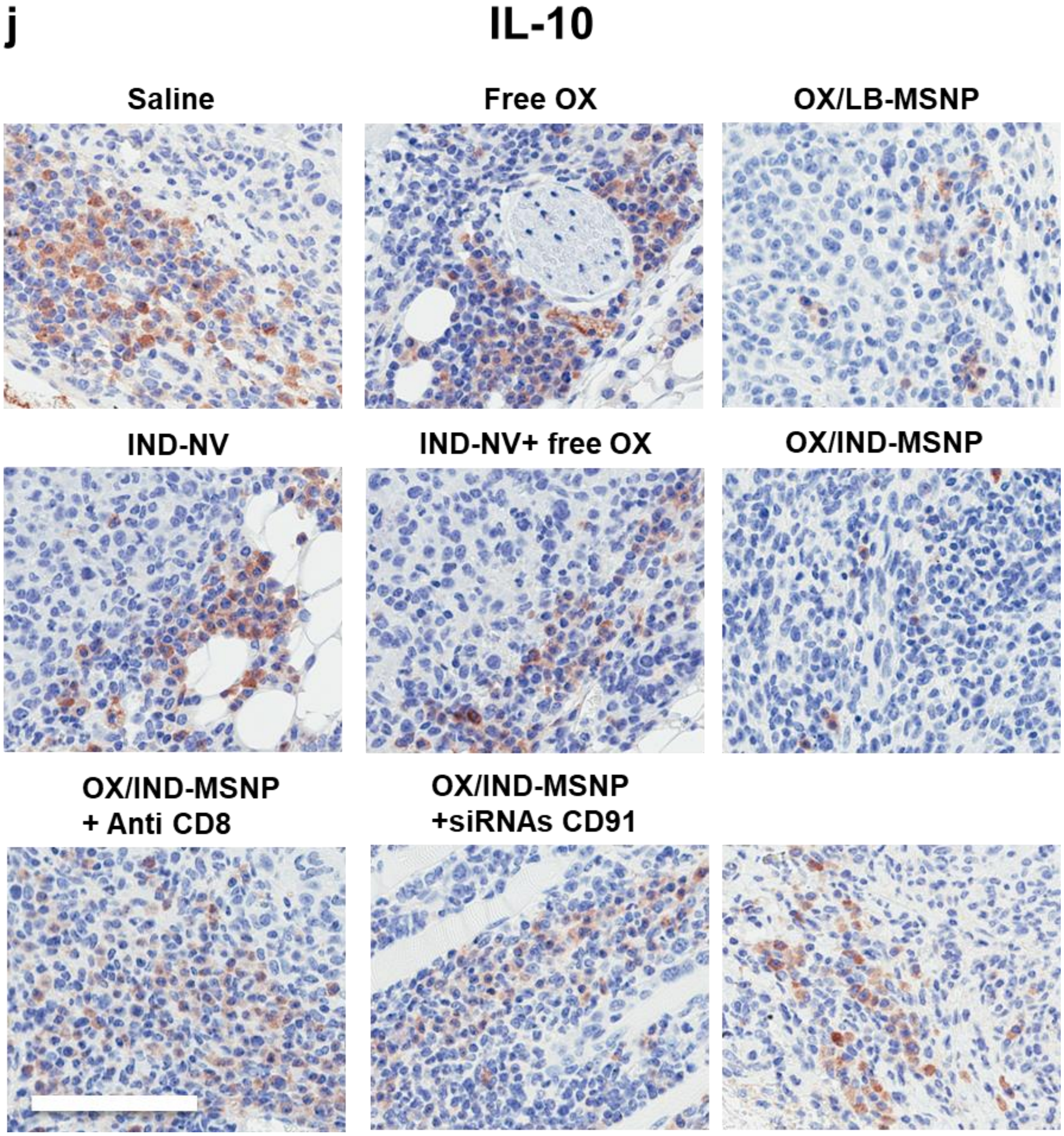
j
IL-10
Saline
Free OX
OX/LB-MSNP
IND-NV
IND-NV+ free OX
OX/IND-MSNP
OX/IND-MSNP
+ Anti CD8
OX/IND-MSNP
+siRNAs CD91

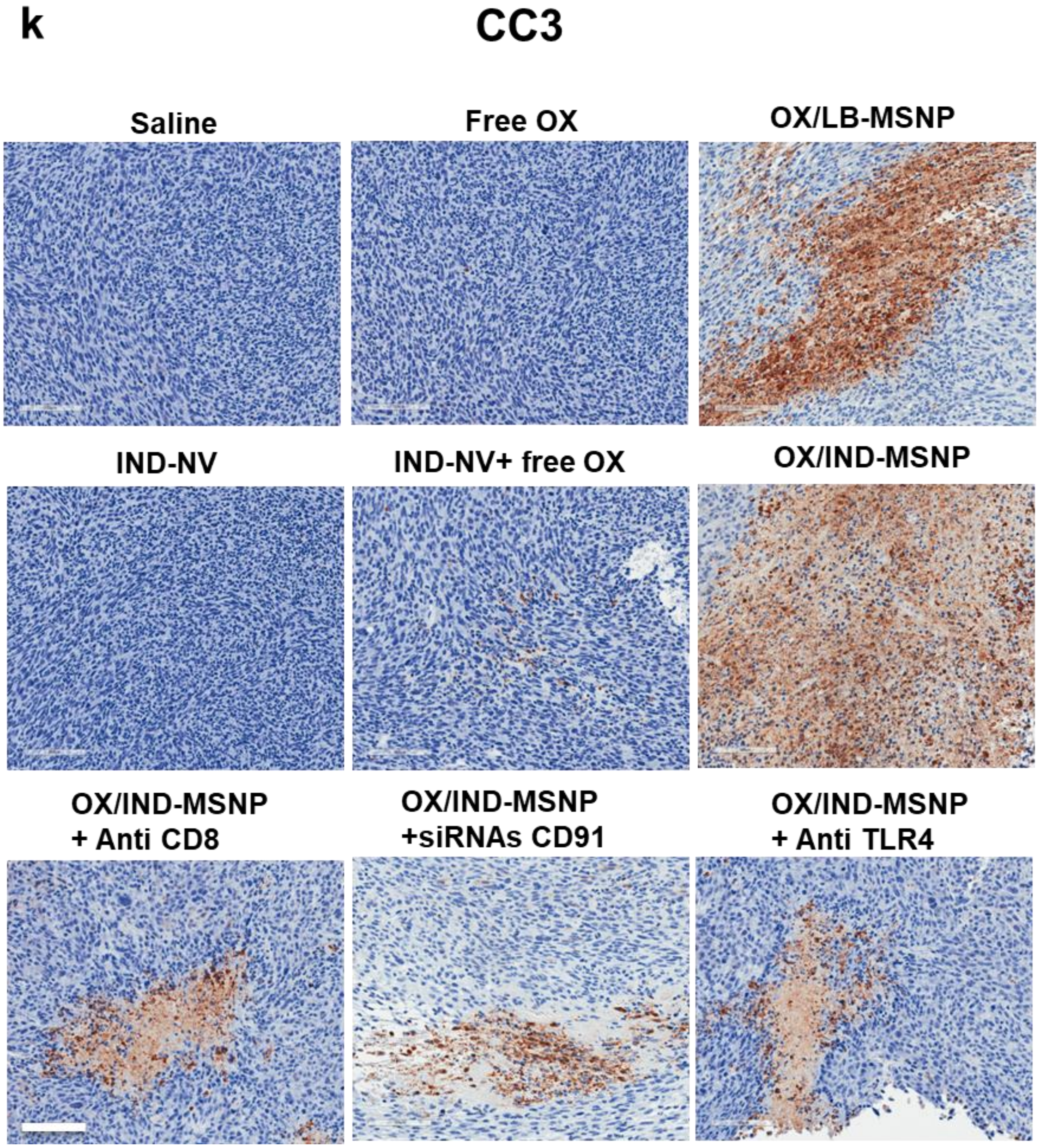
k
CC3
Saline
Free OX
OX/LB-MSNP
IND-NV
IND-NV+ free OX
OX/IND-MSNP
OX/IND-MSNP
+ Anti CD8
OX/IND-MSNP
+siRNAs CD91
OX/IND-MSNP
+ Anti TLR4

I

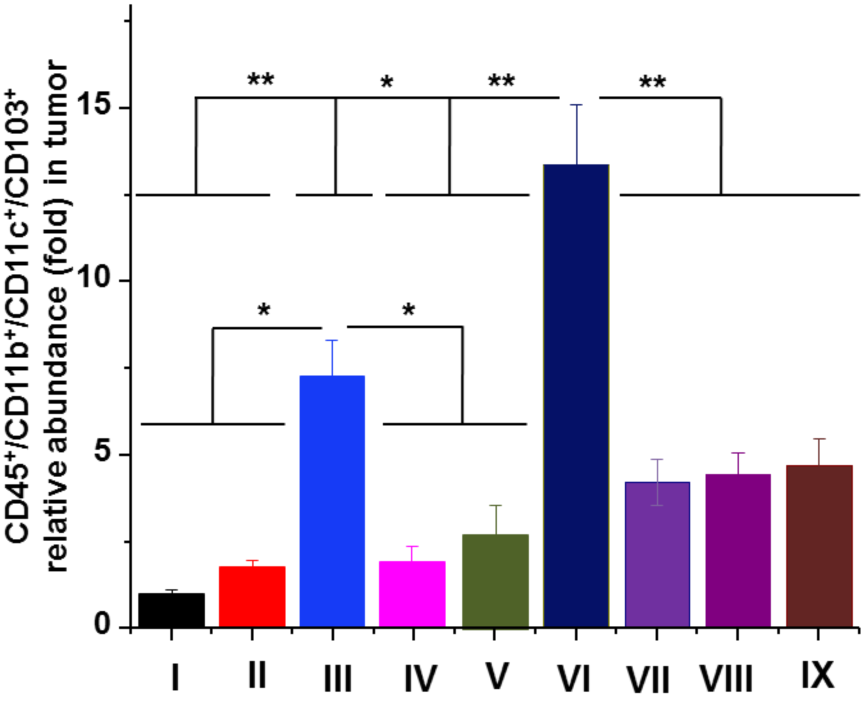

CD45⁺/CD11b⁺/CD11c⁺/CD103⁺ relative abundance (fold) in tumor
0
5
10
15
**
*
**
**
*
*
I
II
III
IV
V
VI
VII
VIII
IX

Supplementary Figure 12 Demonstration of the impact of IV injection of antibodies to CD8 and TLR-4, or an injectable pool of siRNAs targeting CD91 in the systemic biodistribution experiment described in **Fig. 6**. (**a**) Representative IVIS tumor imaging on days 10, 18, 27, and 36 from mice receiving OX/IND-MSNP (5 mg/kg OX and 50 mg/kg IND) with or without treatment, anti-CD8, anti-TLR4 or CD91 siRNA knockdown (n =7). The normalized tumor burden, as reflected by the luminescence intensity in the ROI, was plotted and displayed in the right side panel. (**b**) Representative autopsy results and *ex vivo* bioluminescence imaging intensity is depicted to show the impact of interference in the immune response on tumor growth and metastases. (**c**) Animal survival rate in the same experiment. (**d**) Impact on the CD8/Tregs ratio in the same experiment. Collectively, these data show that interference in both innate (CD91, TLR40) and adaptive (CD8) immunity could significantly reverse the antitumor efficacy of OX/IND-MSNP. *$p < 0.05$; **$p < 0.01$, (ANOVA) compared to saline.

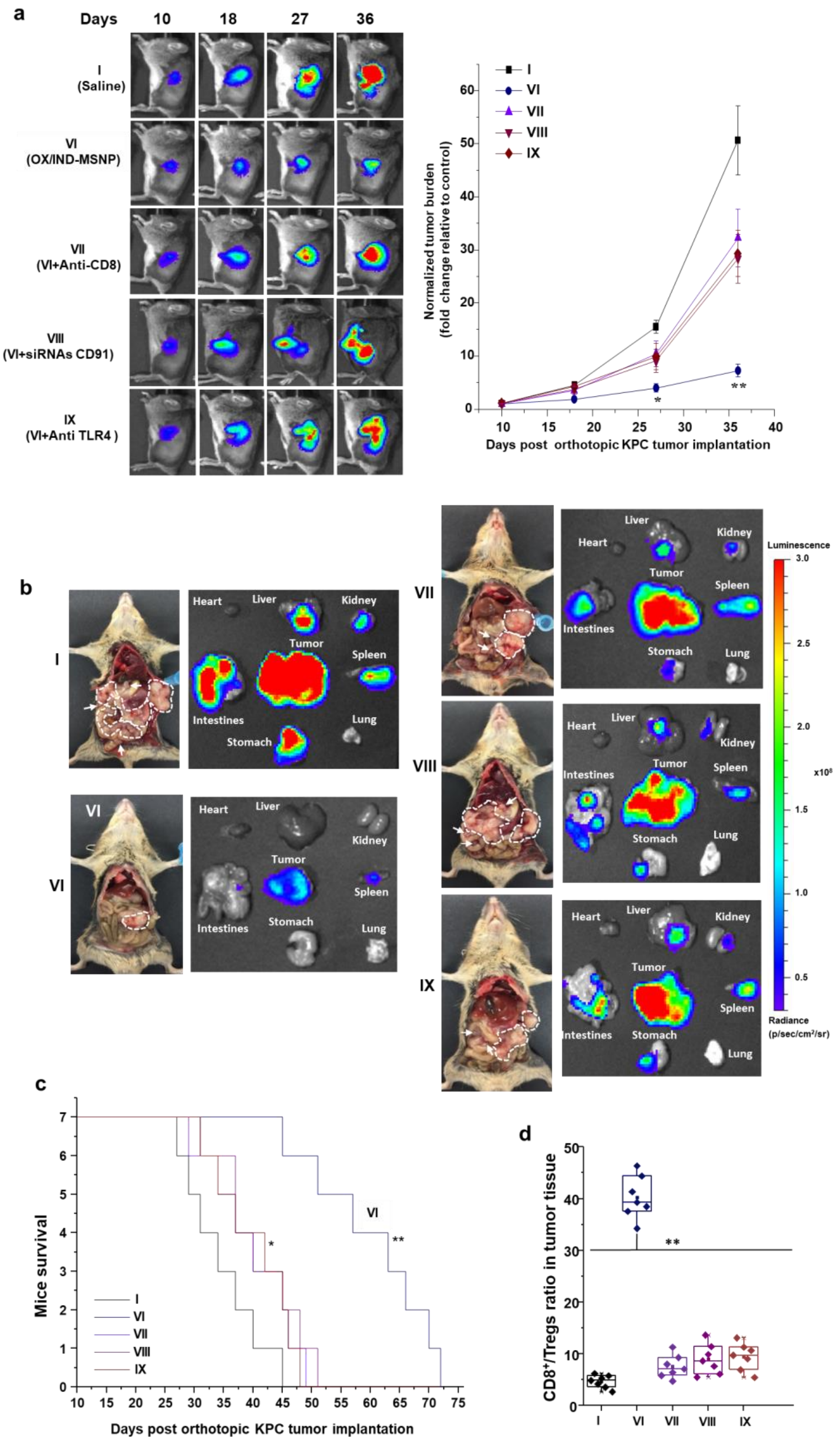

Supplementary Figure 13. (A) Monitoring of animal weight during treatment with the dual delivery carrier in **Fig. 6.** (B) Monitoring of liver enzymes (ALT, AST, and ALP) in the orthotopic tumor model IV described in **Figs. 5** and **S11**. OX/IND-MSNP dual delivery particles did not exert toxicity during the experiment. Instead it helped to protect against liver toxicity from IV injected free OX. Similarly, there was no biochemical evidence of toxicity in the kidney or heart (not shown). ***p* < 0.01, (ANOVA) compared to Saline.

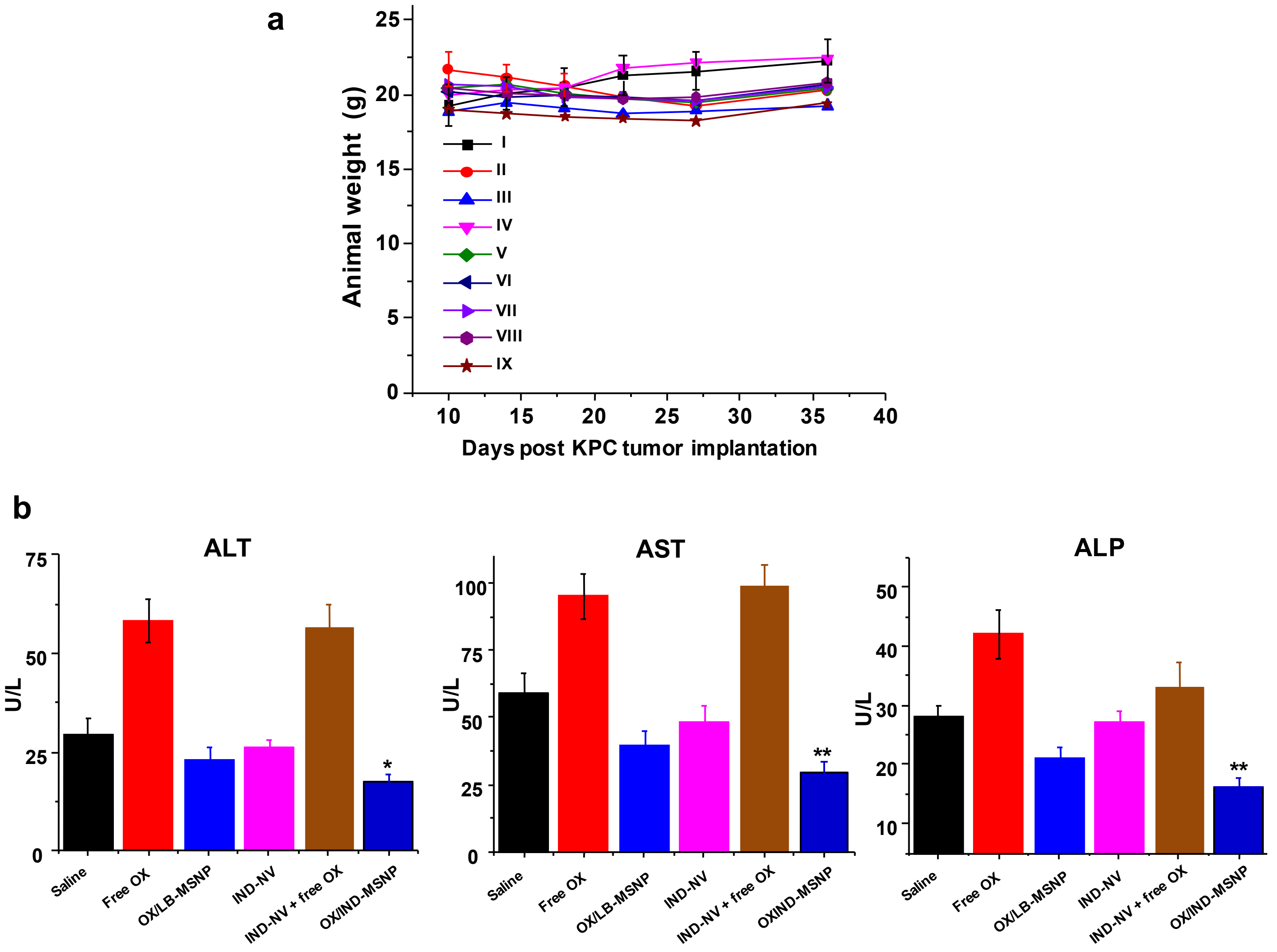

Supplementary Figure 14. Normalized area of interest (ROI) scanning data to show the radio label portioning to the spleen and tumor draining lymph nodes (TDLN) in mice treated by saline, OX/LB-MSNP, and OX/IND-MSNP followed by IV immuno-PET particle (89Zr-malDFO-169 cDb) injection, as described in **Fig. 7.** *p < 0.05; **p < 0.01, (ANOVA) compared to Saline.

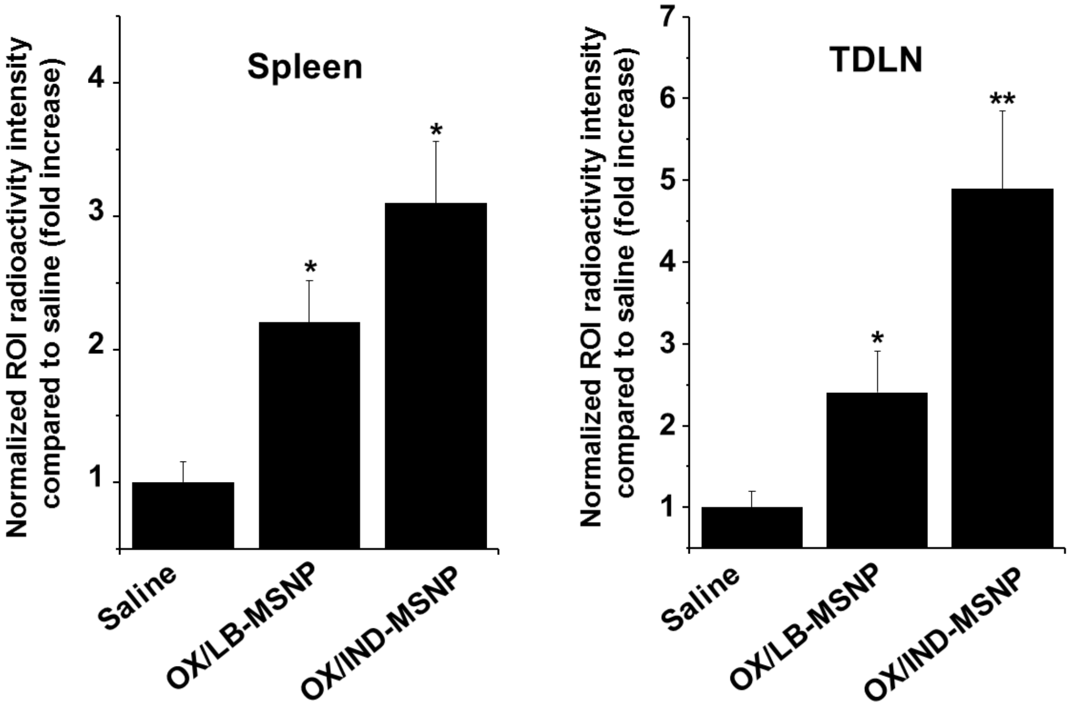

Supplementary References

1. Hingorani SR*, et al.* Trp53R172H and KrasG12D cooperate to promote chromosomal instability and widely metastatic pancreatic ductal adenocarcinoma in mice. *Cancer cell* **7**, 469-483 (2005).

2. Liu X*, et al.* Irinotecan Delivery by Lipid-Coated Mesoporous Silica Nanoparticles Shows Improved Efficacy and Safety over Liposomes for Pancreatic Cancer. *ACS nano* **10**, 2702-2715 (2016).

3. Hou DY*, et al.* Inhibition of indoleamine 2,3-dioxygenase in dendritic cells by stereoisomers of 1-methyl-tryptophan correlates with antitumor responses. *Cancer research* **67**, 792-801 (2007).

4. Metz R*, et al.* IDO inhibits a tryptophan sufficiency signal that stimulates mTOR: A novel IDO effector pathway targeted by D-1-methyl-tryptophan. *Oncoimmunology* **1**, 1460-1468 (2012).

5. Tesniere A*, et al.* Immunogenic death of colon cancer cells treated with oxaliplatin. *Oncogene* **29**, 482-491 (2010).

6. Hrkach J*, et al.* Preclinical development and clinical translation of a PSMA-targeted docetaxel nanoparticle with a differentiated pharmacological profile. *Science translational medicine* **4**, 128ra139 (2012).

7. Lee RE*, et al.* Spectinamides: a new class of semisynthetic antituberculosis agents that overcome native drug efflux. *Nature medicine* **20**, 152-158 (2014).

8. Sparreboom A*, et al.* Comparative preclinical and clinical pharmacokinetics of a cremophor-free, nanoparticle albumin-bound paclitaxel (ABI-007) and paclitaxel formulated in Cremophor (Taxol). *Clinical cancer research : an official journal of the American Association for Cancer Research* **11**, 4136-4143 (2005).

9. Tavare R*, et al.* An Effective Immuno-PET Imaging Method to Monitor CD8-Dependent Responses to Immunotherapy. *Cancer research* **76**, 73-82 (2016).

10. Tavare R*, et al.* Immuno-PET of Murine T Cell Reconstitution Postadoptive Stem Cell Transplantation Using Anti-CD4 and Anti-CD8 Cys-Diabodies. *Journal of nuclear medicine : official publication, Society of Nuclear Medicine* **56**, 1258-1264 (2015).

11. Litzenburger UM*, et al.* Constitutive IDO expression in human cancer is sustained by an autocrine signaling loop involving IL-6, STAT3 and the AHR. *Oncotarget* **5**, 1038-1051 (2014).
